# Estrogen Receptor β2 Oversees Germ Cell Maintenance and Gonadal Sex Differentiation in Medaka, *Oryzias latipes*

**DOI:** 10.1016/j.stemcr.2019.07.013

**Published:** 2019-08-13

**Authors:** Tapas Chakraborty, Sipra Mohapatra, Lin Yan Zhou, Kohei Ohta, Takahiro Matsubara, Taisen Iguchi, Yoshitaka Nagahama

**Affiliations:** 1 South Ehime Fisheries Research Center, Ehime University, Ainan 798-4206, Japan; 2 Laboratory of Reproductive Biology, National Institute for Basic Biology, Okazaki 444-8585, Japan; 3 Key Laboratory of Aquatic Science of Chongqing, School of Life Science, Southwest University, Chongqing 400715, China; 4 Laboratory of Marine Biology, Kyushu University, Fukuoka 812-8581, Japan; 5 Laboratory of Molecular Environmental Endocrinology, Okazaki Institute for Integrative Bioscience, National Institute of Natural Sciences, Okazaki 444-8787, Japan; 6 Nanobioscience, Yokohama City University, Yokohama 236-0027, Japan

**Keywords:** *ERβ2*, germ cell migration, knockdown, sex differentiation, sex reversal, primordial germ cell cell death, autophagy, estrogen, medaka

## Abstract

In vertebrates, estrogen receptors are essential for estrogen-associated early gonadal sex development. Our previous studies revealed sexual dimorphic expression of estrogen receptor β2 (ERβ2) during embryogenesis of medaka, and here we investigated the functional importance of ERβ2 in female gonad development and maintenance using a transgenerational ERβ2-knockdown (ERβ2-KD) line and ERβ2-null mutants. We found that *ERβ2* reduction favored male-biased gene transcription, suppressed female-responsive gene expression, and affected *SDF1a* and *CXCR4b* co-assisted chemotactic primordial germ cell (PGC) migration. Co-overexpression of *SDF1a* and *CXXR4b* restored the ERβ2-KD/KO associated PGC mismigration. Further analysis confirmed that curtailment of ERβ2 increased intracellular Ca^2+^ concentration, disrupted intra- and extracellular calcium homeostasis, and instigated autophagic germ cell degradation and germ cell loss, which in some cases ultimately affected the XX female sexual development. This study is expected improve our understanding of germ cell maintenance and sex spectrum, and hence open new avenues for reproductive disorder management.

## Highlights

• *ERβ2* has a multifaceted role in early gonadal sex differentiation
• *ERβ2* directly influences *SDF1a*/*CXCR4b* PGC chemotaxis and germ cell migration
• ERβ2-KD impairs intra- and extracellular calcium homeostasis and triggers cell death
• In some cases, ERβ2-KD and KO alters sexual development in female gonad

## Teaser

In this article, Chakraborty et al. find that estrogen receptor β2 (ERβ2) plays multifaceted crucial roles in medaka gonadal development and sexuality. ERβ2 predominantly expresses in germ cells, influences the SDF1/CXCR4 chemotaxis, PGC migration, and death, and regulates germ cell proliferation and various sex-biased gene transcriptions that are essential for gonadal sex assignment and maintenance.

## Introduction

Sex steroids and their receptors harmoniously maintain the reproductive physiology, and any disruption in sexual development leads to a huge impact on individual or species physiology (Windsor et al., 2018). The action of the female hormone, estrogen, is mostly mediated by estrogen receptors (ERs), whose action on sterility, infertility, or subfertility and sexual behavior have been thoroughly investigated using selective ER modulators, *ERα-* and *ERβ*-knockout (KO) mice (Bondesson et al., 2015, Chen et al., 2009, Dupont et al., 2000), and *ERα-*, *ERβ1-*, and *ERβ2*-KO zebrafish (Lu et al., 2017). Although *ERβ*s have recently gained popularity in vertebrate folliculogenesis and infertility studies (Antal et al., 2012, Khattri et al., 2009, Lu et al., 2017), their involvement in early gonadal sex differentiation and maintenance requires further investigation.

Germ and somatic cells are two essential variables in gonadal differentiation and sexual identity in mouse, chicken, and fish (DeFalco and Capel, 2009). In vertebrates, primordial germ cells (PGCs) arise at a distant site, divide, migrate through the gut mesentery and bloodstream, and arrive at and colonize at the gonadal primordium during the bipotential stage (DeFalco and Capel, 2009). During early embryonic development, PGCs migrate to the newly formed gonadal anlagen with the help of germ-soma chemotaxis. Notably, cellular chemotaxis is affected by external steroids (Bondesson et al., 2015), ERs (Gamba et al., 2010), and calcium homeostasis (Wu et al., 2009). More or less at the same time, the somatic cells undergo a series of genetic sex- or environmental-dependent modifications and, along with germ cell, decide the gonadal sexuality (DeFalco and Capel, 2009). Furthermore, migrational disruption and depletion of PGC number causes suboptimal PGC settlement in the gonadal primordium and, in turn, blocks female fate and sometimes triggers male development (Tzung et al., 2015). In medaka, an important model species for gonadal sex determination and differentiation studies (Matsuda et al., 2002), the early gonadal settlement of PGCs is regulated by SDF1/CXCR4-mediated chemotactic migration (Herpin et al., 2008, Kurokawa et al., 2007). Later, during medaka sex differentiation, both germ and somatic cells co-operatively regulate the gonadal development in an estrogen-dependent manner (Kurokawa et al., 2007) and help the PGCs to undergo a proliferative mitosis and meiosis in females, while restricting proliferation in males.

Estrogen is known to affect the transcriptional profiles of several major sex-related genes (e.g., *DMRT1*, *GSDF*, Aromatase, *RSPO1*), germ cell proliferation characteristics, and sexual identity in medaka (Chakraborty et al., 2011, Chakraborty et al., 2016, Okubo et al., 2011, Shibata et al., 2010, Zhou et al., 2016). Earlier we found that, during early sex differentiation, *ERβ2* predominantly expresses in the germ cells of embryonic female gonad, while estrogen treatment specifically induces *ERβ2* expression in XY male fish, and thereby speculated the *ERβ2* association in germ cell maintenance (Chakraborty et al., 2011).

This study was conducted to determine the specific roles of *ERβ2* in early germ cell development and its consequences on sexual identity. In brief, we have developed transgenic *ERβ2* knockdown (ERβ2-KD) and knockout (ERβ2-KO) medaka lines and assessed the *ERβ2*-mediated effects on sex differentiation/maintenance and sex reversal. Furthermore, we have ascertained the direct involvement of *ERβ2* in SDF1/CXCR4-mediated chemotactic PGC migration, and calcium homeostasis-related germ cell survival and death, which, in some cases, eventually affects the seeding population of germ cells in the gonadal anlagen and disrupts normal sexual development.

## Results

### Germ Cell Proliferation Is Associated with *ERβ*s

To ascertain the role of *ERβ*s in gonadal development *in vivo*, we treated fertilized embryos with an ERβ-specific agonist (WAY20070, 1 nM), antagonist (cyclofenil, 10 nM), or ethanol (vehicle control) for a period of 18 days (until 10 days after hatching [dah]). Histological analysis depicted a significant reduction in gonad size and germ cell numbers in both agonist- and antagonist-treated fish (Figure S1). Although meiotic cell numbers were slightly higher in agonist-treated fish, the mitotic proliferation reduced drastically in the ERβ agonist-treated than in the ERβ antagonist-treated XX fish. Previously, based on E_2_-dependent *ERβ1* and *ERβ2* expression profiles in medaka embryos, we hypothesized that both these ERβ subtypes work, respectively, on “cessation of male germ cell proliferation” and “mitotic burst in female.” Thus, the present agonist and antagonist (both *in vivo* and *in vitro*; Figure S1) treatments further corroborate that *ERβ1* and *ERβ2* has an antagonistic role in medaka (Chakraborty et al., 2011). Even though histologically no significant phenotypic changes were observed, ERβ agonist selectively reduced the male-dominated genes, i.e., *SOX9a2* and *GSDF*, in the XY fish, while the female-dominated genes (*FOXL2* and *CYP19a1*) were found to decrease in XX females upon antagonist treatment (Figure S1). Interestingly, the correlation (p < 0.05) between histological and transcriptional changes was much higher for ERβ2 (correlation coefficient [CR] = 0.78, n = 15) than ERβ1 (CR = 0.27, n = 15), thus highlighting the importance of *ERβ2* in early medaka gonadogenesis (Chakraborty et al., 2011). This finding is supported by our previous report wherein *ERβ2* showed female-dominated expression in the early sex determination period (Chakraborty et al., 2011).

### ERβ2-KD Restricts Germ Cell Proliferation in Embryonic Medaka Gonad

**Figure 1:**
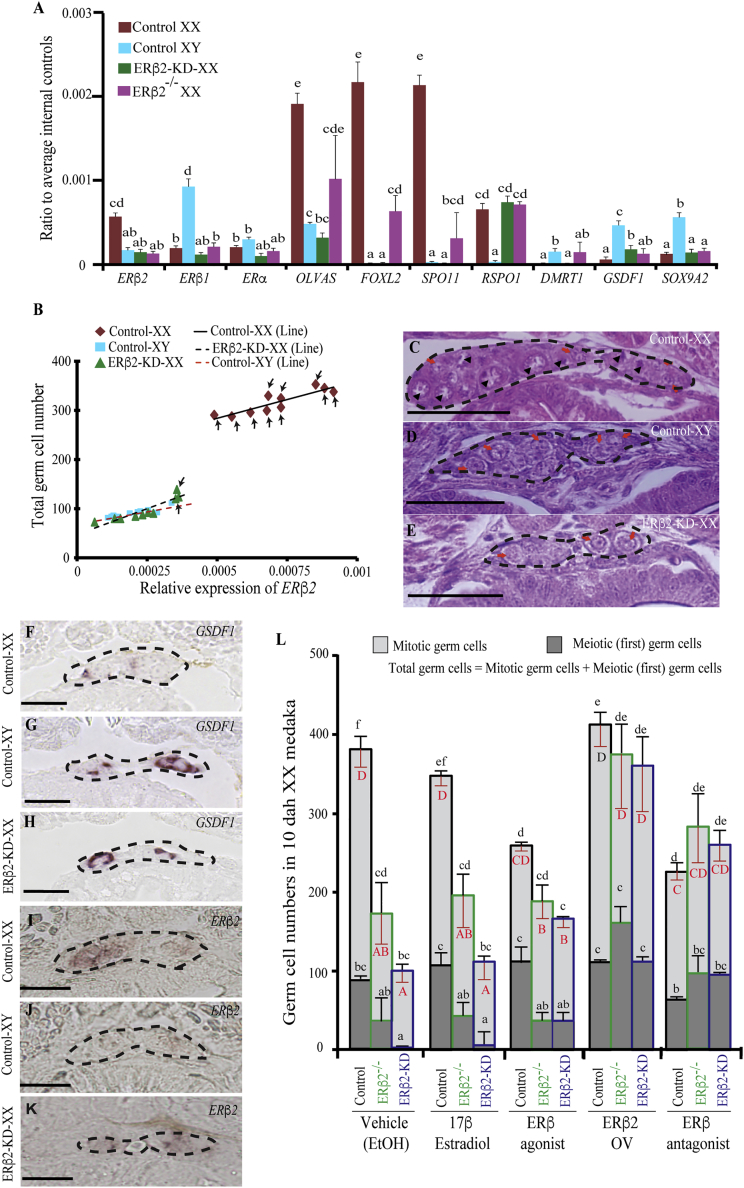
Effects of ERβ2 Reduction on Gonadal Sex Differentiation of XX Medaka at 10 Days after Hatching (A) qPCR (n = 10 pooled samples/group; each pool contains 10 randomly collected individuals) analysis of several sex-specific genes depicted a male-biased transcriptional profile of ERβ2-KD and -KO fish. (B) Germ cell numbers showed a strong correlation with *ERβ2* expression. Chronologically, gonadal germ cell population was determined by confocal imaging, *ERβ2* concentration was measured by qPCR in the same OLVAS-eGFP-ERβ2-KD embryos, and later general linear modeling was used for statistical analysis (n = 10 individual samples/group). The individual gonads that housed the meiotic cells are marked with black arrows. (C–E) Proliferative mitosis and meiosis was evident in control-XX fish (C), while control-XY (D) and ERβ2-KD-XX (E) fish demonstrated male-type gonadal development, characterized by mitotic and meiotic blockage. (F–L) *In situ* hybridization analysis using *GSDF* (F–H) and *ERβ2* (I–K) confirmed the gonadal masculinity. (L) Furthermore, different embryonic treatments, i.e., 17β-estradiol (E_2_, 1 ng/L), *ERβ* agonist (WAY20070, 1 nM), *ERβ* antagonist (Cyclofenil, 10 nM), and *ERβ2* overexpression, were performed using ERβ2-KD-XX and control-XX embryos to rescue the masculine effect of ERβ2-KD. Ethanol (EtOH)-treated samples were used as vehicle control. In graphs, data are plotted as means ± SEM; different letters denote significant differences at p < 0.01. In (L), letters in lower case (a–f) and upper case (A–D), respectively, indicate significant differences among mitotic and meiotic cell population (black, continuous error bars) and total cell population (red, continuous inverted error bars) at p < 0.01. Red arrows indicate candidate mitotic cells while black arrowheads denote the cells undergoing first meiosis. Black dotted lines mark the gonadal boundary. n = 10 fish used for each experiment per group. Scale bars, 100 μm. See also Figures S1–S3 and Table S2.

To determine the importance of *ERβ2* in estrogen-dependent sex differentiation of medaka, we knocked down the *ERβ2* expression (Figure S2) in 1- to -2 cell stage embryo, using a pre-established transgenerational knockdown technology (Chakraborty et al., 2016). We simultaneously scouted the medaka tilling mutant library and generated ERβ2-KO line (Figures S2 and S3). *ERβ2* reduction resulted in an average decrease of *ERβ2* transcript of 67% (66%–80% in females [Figure 1A] and 41%–69% in males). Interestingly, the germ cell number and corresponding *OLVAS* (vasa homolog and medaka germ cell marker) expression (Figures 1B and S3) showed a remarkable direct correlation with *ERβ2* mRNA expression (CR XX = 0.89, CR XY = 0.91, and CR ERβ2-KD-XX = 0.9; p < 0.01). Live confocal imaging and subsequent qPCR analysis of OLVAS-eGFP-ERβ2-KD and control OLVAS-eGFP embryos (Figures 1C–1E), and *in situ* hybridization (ISH) (Figures 1F–1K) demonstrated that ERβ2 reduction not only increased the GSDF abundance in gonadal primordium but also significantly suppressed the mitotic and meiotic germ cell count. Notably, at 10 dah, in control female gonad germ cells undergo rapid mitotic and meiotic proliferation (Figure 1C), while males possess mitotically quiescent sporadically distributed germ cells in the gonad (Figure 1D), resulting in differences among germ cell numbers of both sexes. The ERβ2-KD-XX and ERβ2^−/−^XX fish gonads showed more similarity toward males than females (Figure 1E) and also harbored fewer germ cells in the gonad (Figure 1L). Several authors have suggested that *GSDF* negatively regulates the initiation of meiosis in medaka (Gautier et al., 2011, Shibata et al., 2010), probably by influencing the germ-somatic cell interaction. *In silico* analysis depicted several half-ERE in the *GSDF* promoter sequence, making it a potential target for ERs, and *in vitro* analysis confirmed the *ERβ2*-responsive GSDF promoter activity (Figure S4). qPCR analysis demonstrated a significant increase in *DMRT1* and *GSDF* expression, and simultaneous reduction in several ovarian responsive genes, i.e., *SPO11* and *FOXL2* (Figure 1A) in ERβ2-KD-XX fish. Additionally, we observed sex-biased, but relatively ubiquitous, *ERβ2* expression in germ and various somatic cells during PGC migration and gonadogenesis (Figure S3), which suggests that the action of *ERβ2* might be associated with *GSDF* during gonadal sex differentiation. However, interaction with other ovary-responsive GSDF-linked genes cannot be ruled out.

Our recent data highlight that *RSPO1*, an estrogen-responsive gene, regulates the *GSDF* expression (Chakraborty et al., 2016, Zhou et al., 2016). The unchanged *RSPO1* expression in the ERβ2-KD XX embryos suggests some unknown intricate connections between *RSPO1*, *ERβ2*, and *GSDF* in medaka gonad. Although we observed a male-specific gene expression pattern in ERβ2-KD-XX and ERβ2^−/−^-XX fish, similar alteration in *DMRT1*, *GSDF*, *SPO11*, and *FOXL2* expressions were also noticed upon androgen/aromatase inhibitor (AI) treatment (de Waal et al., 2009). This indicates that *ERβ2* action is associated with either direct blockage of estrogen action or indirect induction of androgen activity/production (de Waal et al., 2009). To confirm this, we treated the control and ERβ2-KD-XX fish with estrogen and *ERβ* agonist (WAY20070). Although estrogen failed, WAY20070 helped to regain both mitotic and meiotic germ cell proliferation of ERβ2-KD fish to some extent (Figure S2). This insinuates that in a receptor-reduced situation, estrogen fails to form receptor-ligand complex and further fails to regulate the ERE-responsive transcription of downstream genes. The mild rescuing effect associated with WAY20070 might be related to simultaneous triggering of both *ERβ1-* and *ERβ2-*mediated pathways (as discussed above), and/or indirect activation of certain mitosis- and meiosis-related genes, which could further influence germ cell proliferation. Thus, to verify the specificity of *ERβ2* knockdown, we injected synthetic (gene sequence was modified to avoid knockdown) ERβ2-eGFP mRNA into 1- to 2-cell stages of ERβ2-KD-XX F_4_ or control embryos. eGFP mRNA was injected into ERβ2-KD-XX embryos, which served as control. Interestingly, we observed nearly 100% rescuing effect in *ERβ2*-overexpressed ERβ2-KD-XX embryos, while no significant influence was noticed in the gonadal development of control groups (Figures 1L and S2). The reoccurrence of gonadal femininity after *ERβ2* overexpression was further confirmed by ISH (Figure S2).

### *ERβ2* Is Critical for Early Gonadal Development

**Figure 2:**
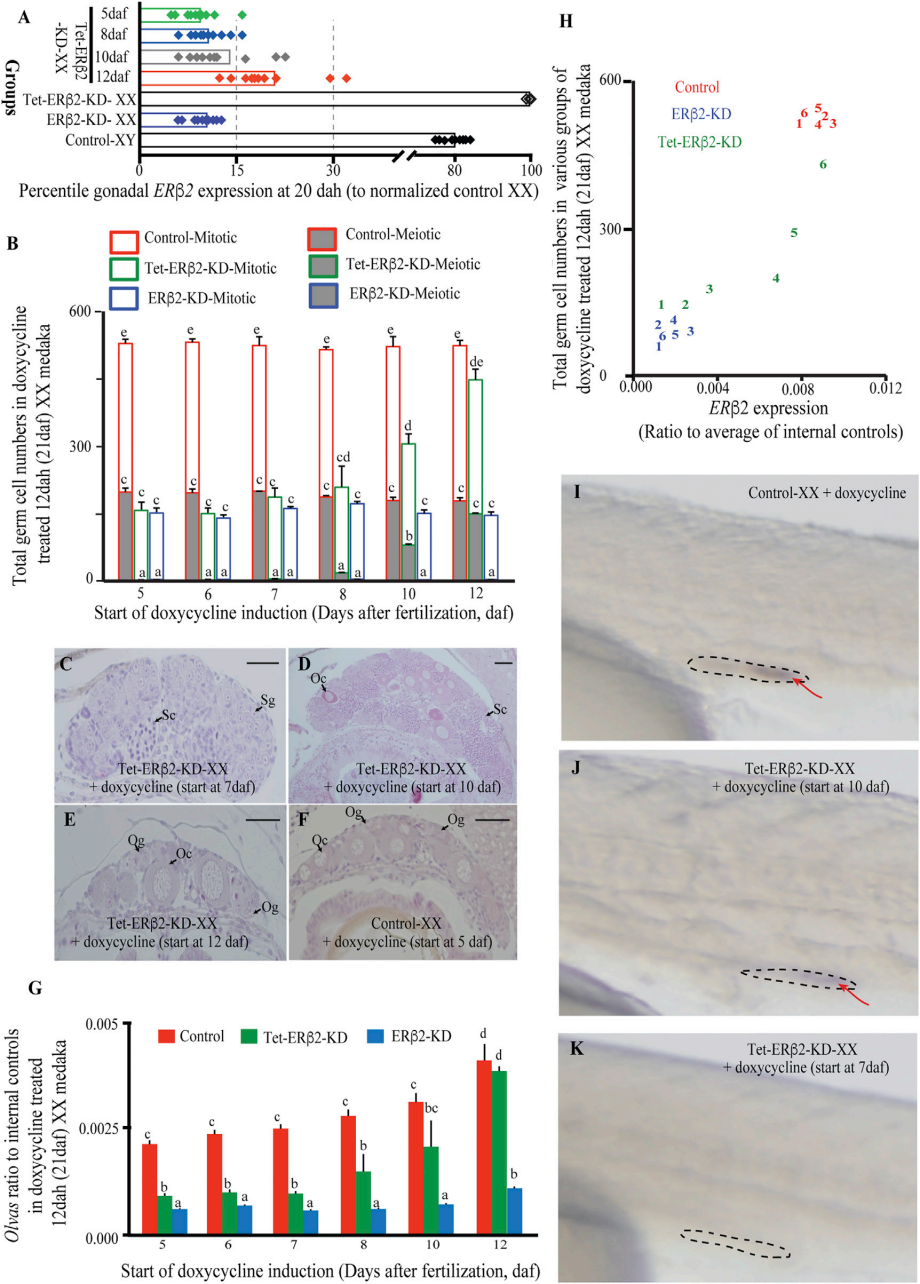
Determination of *ERβ2*-Responsive Critical Window Period of Gonadal Development in Medaka Using Tet-On Knockdown System (A) ERβ2-KD tet-on plasmid-injected embryos were treated with doxycycline from either 5, 6, 7, 8, 10, or 12 daf. Tetracycline knockdown effects at representative time points were evaluated by qPCR analysis of *ERβ2* gene at 20 dah and the data were plotted as percentile reduction against control-XX medaka. (B) At 12 dah, the total germ cell numbers of XX fish were counted and average numbers (n = 18) were plotted against starting days of doxycycline induction to postulate the gonadal sex. (C–G) Histologically, restricted germ cell proliferation was evident in 7-daf groups (C) but germ cell proliferation in 10-daf (D) and 12-daf (E) groups depicted a control-XX-like pattern (F). qPCR analysis (n = 6) of *OLVAS* gene (G) substantiated the histological observations and suggested XX male development. Representative oogonia (Og), oocyte (Oc), spermatogonia (Sg), and spermatocytes (Sc) are marked with black arrows. (H–K) At 12 dah, *ERβ2* expression was measured using qPCR (n = 6) and plotted against average total germ cell numbers of respective treatment groups. In (H), the various treatment groups are marked as 1 (5 dah), 2 (6 dah), 3 (7 dah), 4 (8 dah), 5 (10 dah), and 6 (12 dah). WISH analysis using *ERβ2* at 0 dah further corroborated the sex reversal (I–K). WISH gonadal positions and representative *ERβ2* signal are, respectively, marked with black dotted boundary and red arrows. qPCR analysis was performed using pooled samples, n = 10 individuals/pool. In graphs, data are plotted as means ± SEM; different letters denote significant differences at p < 0.01. Scale bars, 50 μm.

Earlier, we reported that *ERβ2* expression peaks at 7 days after fertilization (daf) in the germ cells of XX medaka (Chakraborty et al., 2011). This implies that *ERβ2* is likely to play a critical role in germ cell maintenance and gonadal development of medaka. To test the hypothesis, we conducted tetracycline (tet)-responsive conditional knockdown of *ERβ2*, induced at different time points during embryogenesis (knockdown efficiency: 65%–92%, depending on stages), and observed a clear decrease in both mitotic and meiotic proliferation (Figures 2A and 2B) as well as *OLVAS* expression in 0–7 daf, which gradually reduced in later stage groups of tet-ERβ2-KD-XX embryos and became non-significant from control female by 10 daf (Figures 2C–2G). Meanwhile, ethanol-treated tet-ERβ2-KD-XX embryos had similar germ cell numbers as the control ethanol-/doxycycline-treated embryos. These data were further confirmed by both qPCR and whole-mount ISH (WISH) of *ERβ2* at 12 dah and 0 dah, respectively (Figures 2H–2K). Notably, *ERβ2* and aromatase co-localizes in female medaka brain to regulate estrogen synthesis (Okubo et al., 2011, Hiraki et al., 2012), thereby validating the fact that *ERβ2* is critical for embryonic gonadal development. Although slightly different, it was observed that zebrafish *ERα* is essential for embryonic PGC mismigration, and the governance of PGC migration is transferred to *ERβ2* in enhanced estrogen condition (Hu et al., 2014). Additionally, recent investigation using zebrafish CRISPR knockout showed that ERβ2 null mutants have relatively slower pace of female gonadal development and male-biased sex ratio than their control counterparts (Lu et al., 2017). All these data highlight that a delicate ER synergy, which might be species specific in some instances or related to intricate organismal-sexual development, is in place to control gonadal development.

### Role of *ERβ2* in Early PGC Migration and Maintenance

**Figure 3:**
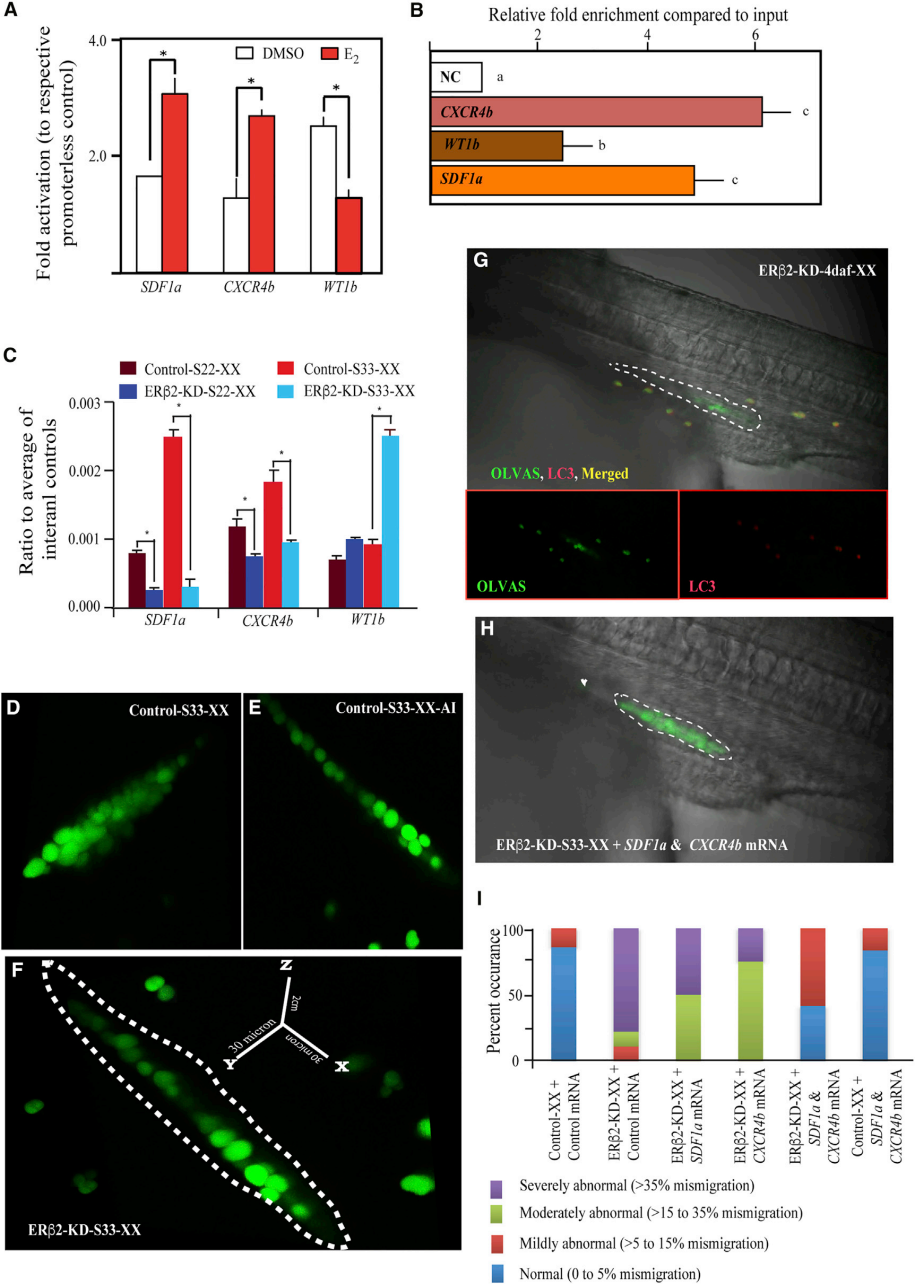
Effect of *ERβ2* on Primordial Germ Cell Fate in Medaka (A) *In vitro* promoter analysis of several primordial germ cell (PGC) migration and maintenance related genes (using HEK-293 cells) showed estrogen/*ERβ2*-dependent modulations (n = 6). E_2_ concentration, 1 ng/mL. (B and C) ChIP assay demonstrated direct relation between *SDF1a*, *CXCR4b*, *WT1b*, and *ERβ2* (C) (n = 3). Comparative qPCR analysis of stage 22 (migrating PGC) and stage 33 (PGC settled in the gonadal primordium) of ERβ2-KD-XX depicted significant downregulation of *SDF1a* and *CXCR4b* at both stages while *WT1b* showed upregulation at stage 33 (B) (n = 10). (D–F) The estrogen/ERβ2-responsive mismigration of PGCs to the gonadal primordium was also confirmed by live confocal z-stage imaging of OLVAS-eGFP-control-XX (D), OLVAS-eGFP-XX-AI (E), and OLVAS-eGFP-ERβ2-KD-XX (F) embryos (generated by crossing ERβ2-KD-XX F_3_ males with OLVAS-GFP-XX females at stage 33). (G) Programmed cell death marker (LC3) confirmed the fate of mismigrated cells at 4 daf. (H and I) Co-injection of *SDF1*a and *CXCR4b* mRNAs (H), but not a singular injection of either *SDF1a* or *CXCR4b* (I), rescued the PGC mismigration. The percentile ratio between total and mismigrating germ cells were subgrouped into normal (0%–5%), mildly abnormal (>5%–15%), moderately abnormal (>15%–35%), and severely abnormal (>35%), and plotted as percent occurrence at group level, to ascertain the rescue effect. qPCR analyses were performed using pooled samples, n = 10 individuals/pool. In graphs, data are plotted as means ± SEM; different letters and asterisks (^∗^) denote significant differences at p < 0.05. The probable gonadal primordia and mismigrating cells are, respectively, marked with white dotted lines and white arrowheads. See also Figure S5 and Table S1.

Our previous data, along with results from tet-ERβ2-KD and ERβ2 expression analysis (Figures 2 and S3) experiments, suggest that the action of *ERβ2* is predominant during 0–7 daf (Chakraborty et al., 2011). This window represents two different major gonadal development-related phenomena, i.e., proliferative mitosis and initiation of meiosis, which in turn determine the phenotypic sex of hatchlings. Total germ cell number in a developing gonad decides the sexual identity (Kurokawa et al., 2007, Tzung et al., 2015). Reports also confirm that the abnormal cell migration is closely related to estrogen and ERs (Gamba et al., 2010, Oviedo et al., 2011). Our present data suggest that *ERβ2* is essential for germ cell population maintenance, and we found severely mismigrated PGCs in the ERβ2-KD embryos (discussed later). To prove the importance of *ERβ2* in PGC migration, firstly, we performed a comprehensive microarray analysis using stage-18 (before the onset of PGC migration) and stage-22 (just after the initiation of PGC migration) control and ERβ2-KD-XX embryos and identified several germ cell migration- and survival-related candidate genes (*SDF1a*, *CXCR4b*, and *WT1b*). Later, we checked the *ERβ2*-responsive and E_2_-dependent promoter activity of these candidates by measuring the luciferase activity, using the HEK-293 cell line. We observed an increased *SDF1a* and *CXCR4b* activity, but reduced *WT1b* expression, upon E_2_ addition (Figure 3A), highlighting the importance of these genes in estrogenic medaka germ cell maintenance (Kurokawa et al., 2007).

Chromatin immunoprecipitation (ChIP) analysis further confirmed that *ERβ2* directly influences the transcription of *SDF1*, *CXCR4*, and *WT1b* (Figure 3B), and thus illuminated the relevance of *E*_*2*_*/ERβ2* in germ cell migration. Our qPCR and WISH (Figures 3C and S5) results showed a significant decrease in *SDF1a* and *CXCR4b*, and elevation in *WT1b* expression in the ERβ2-KD group compared with their respective controls. The transcriptional difference among control and ERβ2-KD-XX fish became wider at stage 33 than stage 22 (Figure 3C). Similarly, morpholino knockdown of *CXCR4b* induces PGC mismigration and slowly causes germ cell reduction (Kurokawa et al., 2007), suggesting that a continuous *ERβ2*-dependent regulation is in place to strictly regulate the germ cell settlement and maintenance. Given the fact that *SDF1* and *CXCR4* expresses in somatic and germ cells, respectively, and play significant roles in PGC maintenance in fish (Herpin et al., 2008, Kurokawa et al., 2007), it is probably likely that *ERβ2* simultaneously manipulates both somatic and germ cells and controls PGC maintenance.

Control-XX fish possessed clustered germ cells, while ERβ2-KD-, ERβ2^−/−^-, and AI-treated XX embryos demonstrated disorganized settlement of germ cells in gonadal primordium (Figures 3D–3F and S5). These observations emphasize that the *ERβ2*-deprived situation created by knockdown mimics the estrogen-reduced situation and becomes critical for maintaining embryonic estrogen action and associated sexual development. In this regard, zygotic estrogen synthesis and actions were found to be critical for embryonic gonadal sexuality (Zhou et al., 2016). Contrastingly, CYP19a1-null mutant medaka did not produce primary sex reversal (sex reversal from early stages), or any germ cell reduction. This highlights that, probably, brain type CYP19b isoform, which also expresses in the gonad during early development, is critical for early estrogen synthesis in medaka. Moreover, our finding suggests that CYP19b actions are impaired in ERβ2-KD embryos (Figure S4), further illuminating the importance of estrogen in early gonadal development. Interestingly, the total *OLVAS* expressions remained unaffected at stages 22 and 33 of ERβ2-KD-XX embryos and significant suppression was noticed at 4 daf, at which point the mislocalized cells became autophagic (Figure 3G). This autophagy induction might be associated with the disturbance in *E*_*2*_*/ER* associated ion homeostasis (Chakraborty et al., 2017).

To interrogate the *ERβ2* involvement in PGC migration, we transplanted the NANOS-dsRED-ERβ2-KD-positive PGCs to the OLVAS-eGFP host and found that only dsRed-positive cells were prone to mismigration and degradation (Figure S5). Germ cell mismigration is associated with the imbalance of *SDF1* and associated *CXCR4* machinery, and both overexpression and knockdown of *SDF1* or knockdown of *CXCR4* triggers PGC mismigration in fish and frog (Doitsidou et al., 2002, Knaut et al., 2003, Herpin et al., 2008, Takeuchi et al., 2010). Similarly, in the present study, co-injection of SDF1a-Cyan and CXCR4b-mCherry mRNAs in 1-cell-stage control and OLVAS-eGFP-ERβ2-KD cross hybrids helped to resume proper migration (Figures 3H and 3I) and further increased germ cell population in the gonad, which could not be achieved with singular injection of either mRNAs (Figures 3I and S5; Table S1). These results, along with the ChIP data, clearly indicate that *ERβ2* is essential for *SDF1a*- and *CXCR4b*-responsive chemotactic migration (Kurokawa et al., 2007) of PGCs and their settlement in gonadal anlagen. However, the germ cell population in the co-injected fish was half that of the normal gonad at 10 dah and also devoid of proper meiotic initiation, thus implying that PGC migration is independent of PGC maintenance and meiosis. The increased *WT1b* activity in ERβ2-KD embryonic gonads at later stages might be a possible factor in the germ cell survivability (Chakraborty et al., 2016).

### ERβ2 Knockdown Disrupts Cellular Calcium Balance and Induces Cell Death

**Figure 4:**
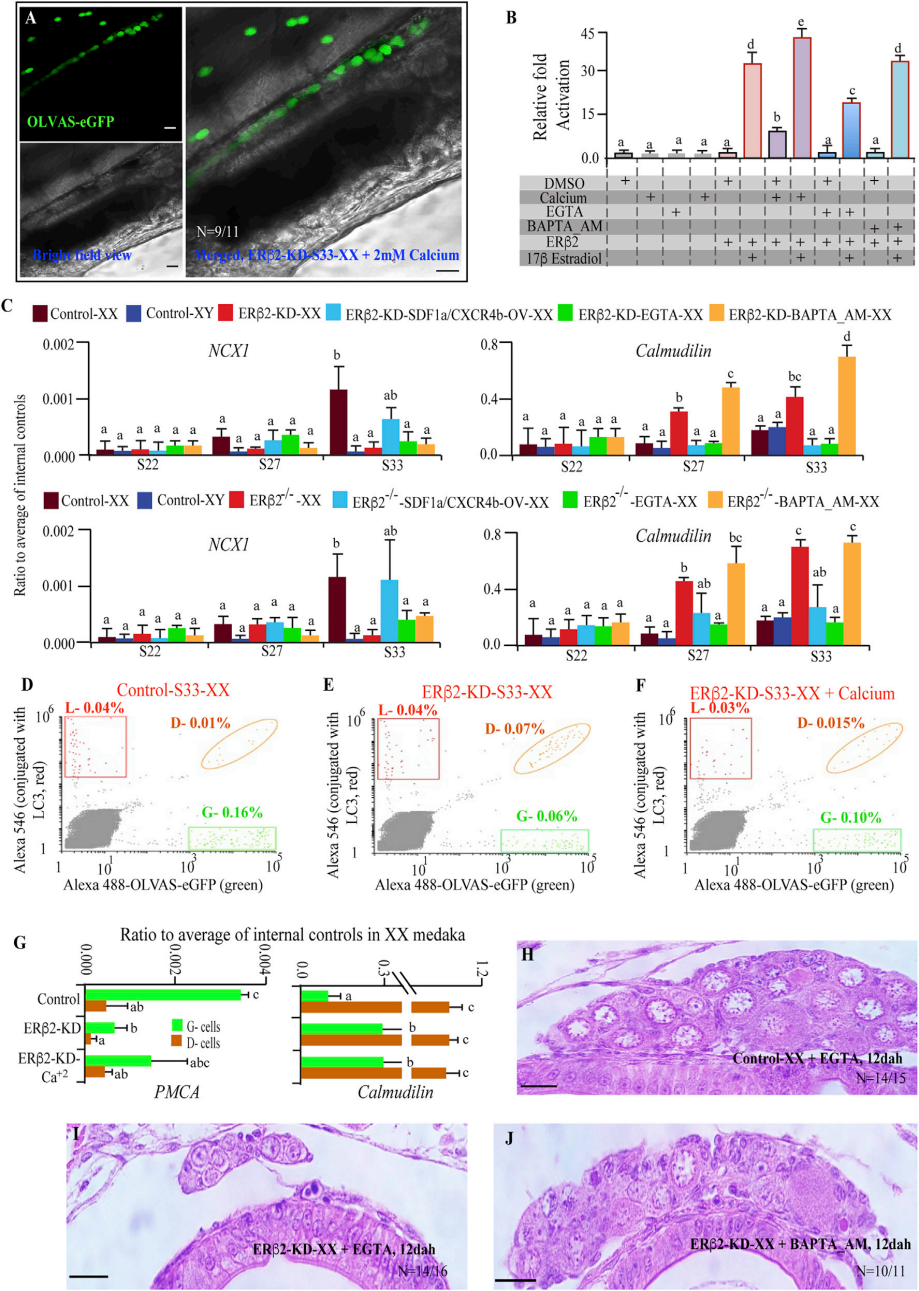
Effect of *ERβ2* Calcium Homeostasis and Germ Cell Death (A) Severe PGC mismigration was recorded in CaCl_2_ (2 mM)-treated ERβ2-KD-XX S33 embryos (n = 11). (B) *In vitro* ERE-dependent ERβ2 transcriptional analysis (using HEK-293 cells, and various combination of 2 mM CaCl_2_, 5 pM EGTA, 10 pM BAPTA-AM, 1 ng/mL 17β-estradiol, and 100 ng of ERβ2 overexpression plasmid) demonstrated that cellular calcium concentration significantly alters the E_2_-responsive *ERβ2* transcription. (C) qPCR analysis (n = 10 individuals/group) of several calcium transport-related genes showed significant alteration in both ERβ2-KD (upper panel) and ERβ2^−/−^ (lower panel) fish than their control, SDF1a/CXCR4b-overexpressed, or intra-/extracellular Ca^2+^-reduced counterparts. (D–F) Control-XX-treated (D), ERβ2-KD-XX-treated (E), and CaCl_2_-treated (F) ERβ2-KD-XX embryonic (stage 33) single-cell suspensions were sorted, and three groups of cells (L, D, and G) were collected (n = 4). (G) The mRNA transcription profile of PMCA1b and CaM were analyzed using qPCR of populations D and G cells to discern the difference between LC3-positive and -negative PGCs. (H–J) ERβ2-KD-XX or control-XX embryos were incubated in BAPTA_AM (10 pM) and EGTA (5 pM) solution until hatching, and gonadal histology was performed at 12 dah (n = 9) to visualize the germ cell proliferation status. PGC migration was analyzed using confocal microscopy. In graphs, data are plotted as means ± SEM; different letters denote significant differences at p < 0.05. BAPTA_AM and EGTA were used as intracellular and extracellular Ca^2+^ chelator, respectively. Control and OLVAS-eGFP-ERβ2-KD fish lines were used for the experiments. Scale bars, 20 μm.

*ER*s, *SDF1a*, and *CXCR4b* are largely associated with calcium signaling, and the latter two are known to cause cell death through the Ca^2+^-signaling pathway (Gamba et al., 2010, Teicher and Fricher, 2010). Moreover, our microarray analysis revealed that several Ca^2+^-signaling-related genes, e.g., *plasma membrane Ca*^2+^ *ATPase* (*PMCA*) *1b*, and *plasma membrane Na*^+^*/Ca*^2+^ *exchanger* (*NCX*) *1*, and *Calmodulin* (*CaM*) (Zhang et al., 2012), were significantly altered in the ERβ2-KD-XX fish. To prove our hypothesis that calcium is indispensable for ERβ2-associated cell death, we incubated the OLVAS-eGFP-ERβ2-KD embryo (from 1-cell stage) in CaCl_2_ (2 mM) solution and checked the PGC migration at stage 33. Unexpectedly, the migration remained identical to that of their ERβ2-KD control counterpart (Figure 4A). Although *NCX1* and *PMCA1b* transcription were significantly reduced in ERβ2-KD-XX, *CaM* showed a pattern completely opposite to that of both control-XX and ERβ2-KD-SDF1/CXCR4b-overexpressed (OV)-XX (at stage 33; Figure 4C). These opposite transcriptional alteration patterns are probably related to their differential role in calcium transport, i.e., Ca^2+^ extrusion (*NCX1* and *PMCA1b*; Brini and Carafoli, 2011) and influx inhibition (*CaM*; Ben-Johny and Yue, 2014, Chi et al., 2017). This, along with calcium homeostasis-related gene transcriptions in ERβ2^−/−^ XX (Figure 4C), implies that *ERβ2* reduction overloads the germ cells with Ca^2+^ by suppressing the transcriptions of Ca^2+^ outflux-related genes and triggers a secondary negative regulatory mechanism, via *CaM* (Griffith et al., 2016), to eventually control the exponential overload. In a subsequent experiment, we reduced the intracellular Ca^2+^ of ERβ2-KD fish by BAPTA_AM, and recorded a substantial decrease in *CaM* level, thus validating the germ cell Ca^2+^ overloading theory. On the contrary, extracellular Ca^2+^ chelation by EGTA did not bring about any significant changes. Further *in vitro* analysis showed that E_2_/ERE-responsive *ERβ2* transcription was accelerated by Ca^2+^, unaffected by intracellular Ca^2+^ chelation, and reduced by extracellular Ca^2+^ chelation (Figure 4B), thereby highlighting the importance of Ca^2+^ influx-outflux ratio in *ERβ2* transcription management (Figure S6). To solve the puzzle of whether addition of excessive Ca^2+^ balances the intracellular and extracellular calcium ion concentration, and further reduces the cell death in mismigrated cells, we sorted three different groups (control-, ERβ2-KD-XX-, and CaCl_2_-treated ERβ2-KD-XX) of stage-33 OLVAS-eGFP embryonic single-cell suspension, stained with Alexa 488-GFP and Alexa 546-LC3 conjugates, and collected three populations of cells, namely, L (LC3 positive), D (LC3 and eGFP positive), and G (eGFP positive). Morphologically, D- and G-cell populations were similar to PGCs, while L had more likeness with the somatic cells. Moreover, ERβ2-KD-S33-XX fish showed lowest G-cell portion and highest number of D-type cells (Figures 4D–4F). The significantly diminished D population after calcium addition (Figure 4F) further confirmed that cell death was indeed reduced by calcium. qPCR analysis of each cell population depicted a substantially elevated *CaM* level in D population than G-cell fraction, while *PMCA1b* transcription was further suppressed in LC3-positive germ cells (Figure 4G). Our data suggest that cellular-level calcium ion threshold is critical for cell survivability (Borodkina et al., 2016, Chakraborty et al., 2017). Longer incubation with BAPTA_AM, and not with EGTA, increased the ERβ2-KD-XX gonad size and germ cell numbers, further emphasizing that intracellular Ca^2+^ overload is crucial for increased PGC degeneration and further germ cell loss (Figures 4H–4J). As mentioned earlier, ERβ2-KD donor cells, upon being transplanted into control-XX embryos, showed that the majority of donor cells mismigrated and became apoptotic, while the host germ cells did not show such a phenomenon (Figure S5). Most likely, in the ERβ2-reduced situation, when Ca^2+^ imbalance was irreparable, the defective cells underwent programmed cell death. Thus, these findings validate the importance of ERβ2 in germ cell migration and maintenance.

**Figure 5:**
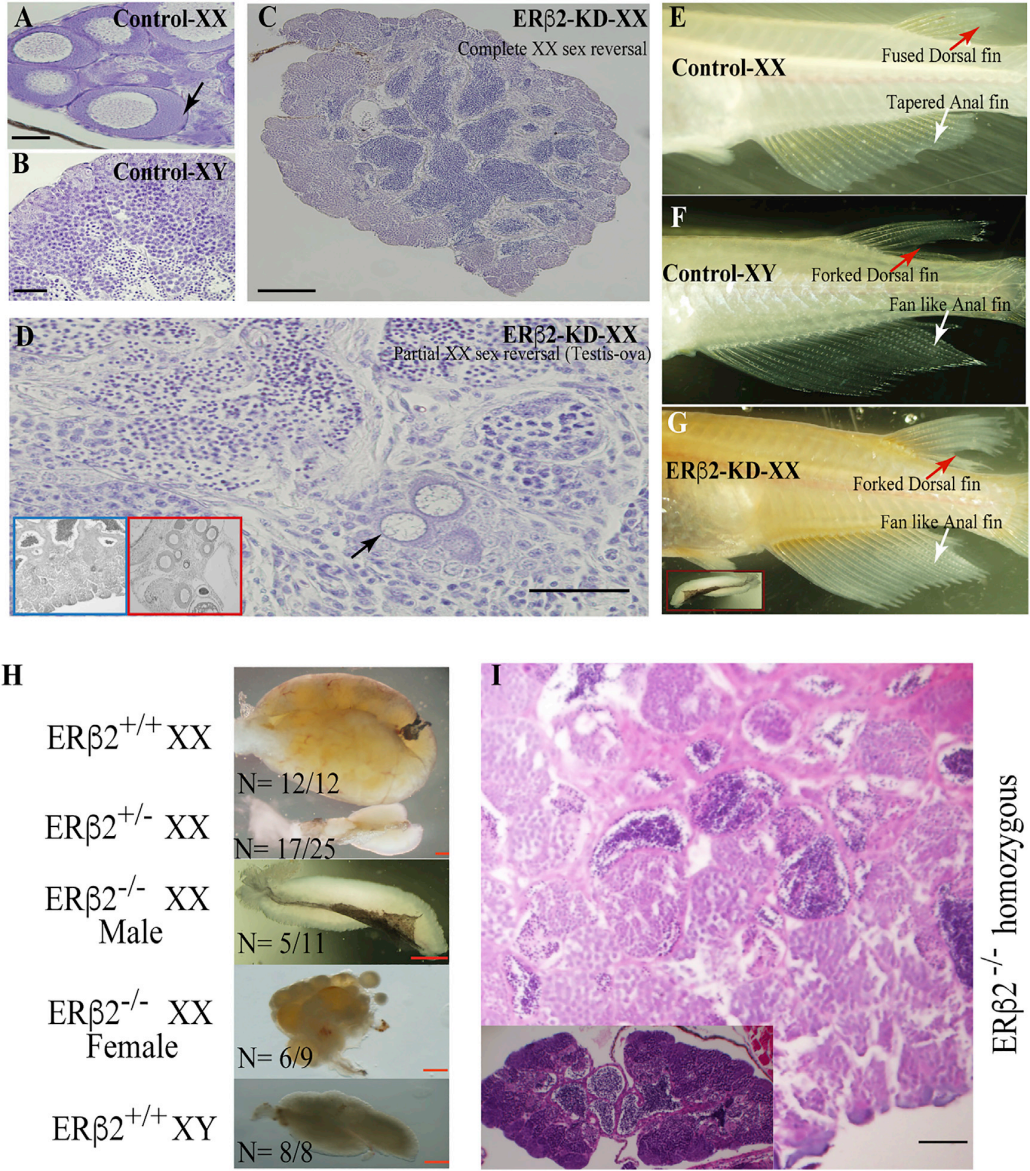
*ERβ2-KD* Affects Gonadal Development in Adulthood (A–D) Histological analysis of control-XX (A), control-XY (B), and ERβ2-KD-XX fish showed evidence that knockdown of *ERβ2* results in partial (D) to complete (C) testis formation in XX adults. Inset: representative low-magnification photomicrograph of testicular (blue boundary) and ovarian (red boundary) prevalent gonadal areas. (E and F) In adulthood, control-XX medaka possesses fused dorsal fin and tapering anal fin (E), while control-XY medaka displays forked dorsal fin and fan-like anal fin (F). (G) The secondary sexual characters of ERβ2-KD-XX fish resemble those of the control-XY fish. Inset: fully grown adult ERβ2-KD-XX testis. (H–I) Phenotypic (H) and histological (I) analysis demonstrated that ERβ2^−/−^ null mutation also results in altered gonadal development and functional testis formation in adulthood. Black, red, and white arrows indicate oocytes, dorsal fin, and anal fin, respectively. Scale bars, 50 μm. See also Figure S6 and Table S2.

Despite significant effects on PGC maintenance and initiation of primary gonadal sex reversal, when analyzed at adult stages approximately 25%–47% of fish possessed male-like secondary sexual characteristics (fan-like anal fin and forked dorsal fin; Figures 5E–5G), 14%–30% of ERβ2-KD-XX and ERβ2^−/−^-XX fish showed complete testis (Figures 5C and 5H–5I; Table 1), and another 10%–17% had testis-ova (Figure 5D and Table 1). ISH and qPCR analysis of XX testis showed an inverse expression pattern between male- and female-responsive genes, except for *RSPO1* and *β-catenin* (Figure S6), a phenomenon similar to early-stage embryos. However, only 25% (3/12) of adult ERβ2-KD F_0_ fish had integration of KD cassette and were able to produce viable progeny. The F_5_ and subsequent progenies generated from the three F_0_ phenotypic males were used for all our experiments. The XX fertile male production increased in later generations (Table S2) when the ERβ2-KD-XX males were mated with XX normal females, further cementing the significance of *ERβ2* in gonadal sex development and maintenance.

**Table 1:** Summary of Gonadal Sex Profile in Various Experimental Fish at Adulthood

Groups^a^	Full-Grown Ovary (%)	Abnormal Ovary^b^ (%)	Testis (%)	Testis-Ova (%)	Breeding Behavior^c^ (%)
Control-XX	65/66 (98.5)	1/66 (1.5)	0/66 (0)	0/66 (0)	A: 0/10 (0) B: 10/10 (100) C: 0/10 (0)
ERβ2-KD (F_10_ generation)	18/115 (15.7)	50/115 (43.5)	35/115 (30.4)	12/115 (10.4)	A: 8/12 (75) B: 0/12 (0) C: 4/12 (25)
ERβ2^+/+^ XX (F_11_ generation)	28/28 (100)	0/28 (0)	0/0 (0)	0/28 (0)	A: 0/8 (0) B: 8/8 (100) C: 0/8 (0)
ERβ2^+/−^ XX (F_11_ generation)	26/51 (51.0)	21/51 (41.1)	0/51 (0)	4/51 (7.9)	A: 2/15 (13.3) B: 10/15 (66.7) C: 3/15 (20)
ERβ2^−/−^ XX (F_11_ generation)	11/35 (31.4)	13/35 (37.1)	5/35 (14.3)	6/35 (17.1)	A: 5/14 (35.7) B: 4/14 (28.6) C: 5/14 (35.7)

a Four different populations of fish, generated from four different parents, were mixed and sampled randomly for histological analysis to ascertain the gonadal sexual status and sex reversal. All the examined fish were screened with DMY-specific genomic PCR and, whenever necessary, followed by ERβ2-mutation-specific PCR and sequencing.

b Vitellogenesis was not initiated, relatively hypotrophic (less) germ cell proliferation, etc.

c Randomly selected fish (from the aforementioned pool of screened fish) were examined for secondary sexual characteristics and tested with mature normal fish of opposite sex. A, B, and C, respectively denote male-like, female-like, and no sexual behavior.

## Discussion

In vertebrates, *ER*s and their ligands play several important and conserved physiological functions. Furthermore, maternal estrogen is known to have a critical role in early development and differentiation (Adkins-Regan et al., 1995). Numerous reports suggest that estrogen is synthesized in the mammalian fetus, especially in the brain, and helps in the smooth execution of *ER*-regulated estrogenic functions (Bondesson et al., 2015). Recently, we reported that estrogen is indeed synthesized in the developing medaka embryos (Zhou et al., 2016), interestingly at the same time as zygotic *ER* and brain type aromatase (*CYP19b*) transcriptional onset (Chakraborty et al., 2011, Okubo et al., 2011), thus emphasizing that fetal estrogenic concentration and actions are instrumental in gonadal sex differentiation in medaka (Zhou et al., 2016). In this work, we found that the ER-agonist, ER-antagonist, ER-KD, and ER-null mutation affect the estrogenic actions in medaka from very early stages of embryonic development, thus establishing the fact that E_2_/ER actions are critical for embryonic gonadal development.

Conversely, Nakamoto et al. (2018) found that null mutation of CYP19a1 gene does not affect the early gonadal development. In medaka, despite the rapid reduction in maternal estrogen from yolk stores, it is probable that traces of remaining estrogen or other estrogen by-products might be enough to drive the normal gonadal development in CYP19a1^−/−^ medaka. Moreover, chronologically, first zygotic expression of *CYP19b* (2 daf) and *ERβ2* (2 daf) precedes the *CYP19a1* expression (5–10 dah), and ERβ2 knockdown reduces the *CYP19b* expression in both gonad and brain. It is possible that during early development estrogen synthesis is mainly regulated by *CYP19b* or some other estrogen-like machinery (Nakamoto et al., 2018). Notably, it has been repeatedly shown that environmental estrogenic chemicals can mimic endogenous estrogen's action and alter sexual development in various organisms, suggesting that estrogen actions are vital for sexual differentiation (Mizoguchi and Valenzuela, 2016). Further investigations to decipher the sources of endogenous estrogenic components are necessary to completely understand the estrogenic role in early gonadal development.

In this work, we found that co-dependent actions of newly synthesized estrogen and *ERβ2* is vital for PGC chemotactic migration, gonadal settlement, maintenance, and proliferation. The abundance of *ERβ2* in both somatic and germ cells further accentuates the germ-soma interactions in sex management. Our data suggest that, during early embryonic development, *ERβ2* produced in the somatic cells directly regulates *SDF1a* transcription and simultaneously modulates *CXCR4b* actions in germ cells to control the directed migration of PGCs (Herpin et al., 2008, Kurokawa et al., 2007). In an ERβ2-reduced situation, the production of both *SDF1a* and *CXCR4* were directly hampered and affected the mismigration, as evidenced by rescuing of PGC mismigration by co-overexpression of both *SDF1a* and *CXCR4*. However, studies show that SDF1 knockdown or overexpression can induce PGC mismigration, thus emphasizing that SDF1 concentrations and associated CXCR4 receptor saturation are critical for proper PGC migration in vertebrates (Herpin et al., 2008, Takeuchi et al., 2010). Although the critical concentration of *SDF1a* in medaka PGC migration still needs confirmation, in the present investigation we observed that *SDF1a* overexpression might have restored the *SDF1a* balance in somatic cells to resume proper migration. In this regard, Herpin et al. (2008) found that 30% or more *SDF1a* knockdown can affect PGC migration in medaka. It is possible that ERβ2 assisted the reduction of both SDF1a and that CXCR4 affected other receptors (e.g., CXCR7) and further aggravated the situation (Boldajipour et al., 2008). The reduction in *ERβ2* also might have affected the *E*_*2*_-induced-*ER*-responsive AKT phosphorylation (Stabile et al., 2006) and further reduced the PGC migration into gonadal anlagen (Moe-Behrens et al., 2003).

In an exemplary study, using morpholino knockdown and cell transplantation, Tzung et al. (2015) demonstrated that the threshold number of PGC is required to retain ovarian stability in zebrafish and is instrumental for testicular differentiation. In our ERβ2-KD fish, PGC mismigration and cell death likely significantly reduce the initial PGC number in the embryonic gonad and mimic a situation similar to male-type gonad in medaka. This gonadal state is further aggravated by the reduced estrogen signaling-associated transcription of male-biased genes. However, in this regard the indirect involvement of the interactively modulated AR pathway (Wu et al., 2017) cannot be overlooked. Since *ERβ2* actions are critical throughout embryogenesis, it is possible that elimination of *ERβ2* affects several other important pathways, and in turn orchestrates the sex reversal. Despite a significant increase in male-biased gene transcription and germ cell reduction, both initiated by *ERβ2* reduction, the percentages of adult sex reversal were somewhat less than expected. This suggests that some unknown mechanism, probably variable non-genomic action or substitution effect or a threshold of gene(s)/pathway(s), are in action, which needs further investigation. In contrast to previous reports on the absence of sex-reversal phenotype in ER-KO mice (Bondesson et al., 2015, Dupont et al., 2000, Hamilton et al., 2014), it was recently found that biallelic/monoallelic mutation of *ERβ* causes sex reversal in humans (Baetens et al., 2018). This difference has been suggestively attributed to the non-genomic ER action, i.e., MAPK signaling (Baetens et al., 2018). Moreover, the intensity of sex reversal in humans is significantly related to zygosity, further supporting the idea that a threshold limit might be important for functional manifestation of *ERβ2* actions in medaka. Nevertheless, irrespective of different non-genomic interaction or fish-specific genome duplication, it seems that the estrogen/ER pathway directly controls the reproductive fitness in both human and medaka. Hence, this study will help us to rethink the estrogen/*ER* involvement in successful reproduction and reproductive disorder management.

In conclusion, our data suggest that *ERβ2* has multi-point regulation, starting from regulation of chemotactic germ cell migration, calcium homeostasis, and cell sustenance, to controlling meiotic initiation, which eventually influences sexual development. Our data also highlight the importance of *ERβ2* in estrogen transmission to gonad to maintain the gonadal sexuality. Thus, this study might be a key to comprehending the diverse estrogen-reproduction relationship in vertebrates.

## Experimental Procedures

### Ethics Statement

All treatments of animals in this study followed the guidelines of the National Institute for Basic Biology and were approved by the Institutional Animal Care and Use Committee of National Institutes of Natural Sciences and Ehime University Animal Use and Ethics Committee. All surgery was performed under Tricaine-S anesthesia, and all efforts were made to minimize suffering.

### Experiments

The plasmids were constructed using various commercially available vectors as required. ERβ2 knockdown was carried out using a previously published protocol (Chakraborty et al., 2016). *In vitro* and *in vivo* analysis was carried out using HEK-293 cells and several medaka strains, respectively. Histological, qPCR (primer details in Table S3), ChIP, germ cell transplantation, flow cytometry, and cell-sorting analysis were performed using pre-adjusted protocols. All the *in vivo* samples were first examined for genetic sex and then pooled (sexwise and/or groupwise) or individually used for RNA isolation and cDNA synthesis, and further subjected to subsequent analysis. A detailed description of procedures used in this study is provided in Supplemental Information.

## Author Contributions

T.C., S.M., and L.Y.Z. contributed equally. T.C., T.I., and Y.N. conceived the idea. T.C., S.M., and L.Y.Z. performed the experiments and statistical analysis. All authors (except L.Y.Z.) provided chemicals and materials for experiments. T.C., S.M., K.O., and Y.N. prepared the manuscript.

## Supplemental Information

Document S1. Supplemental Experimental Procedures, Figures S1–S6, and Tables S1–S3

Document S2. Article plus Supplemental Information
