# Supplementary material for: Estrogen Receptor β2 Oversees Germ Cell Maintenance and Gonadal Sex Differentiation in Medaka, Oryzias latipes
Source: Stem Cell Reports. 2019 Aug 13;13(2):419–33. doi: 10.1016/j.stemcr.2019.07.013 (PMC6700524; doi:10.1016/j.stemcr.2019.07.013)
Supplement: Document S1. Supplemental Experimental Procedures, Figures S1–S6, and Tables S1–S3 [file mmc1.pdf]

Stem Cell Reports, Volume *13*

Supplemental Information

Estrogen Receptor β2 Oversees Germ Cell Maintenance and Gonadal Sex Differentiation in Medaka, *Oryzias latipes*

Tapas Chakraborty, Sipra Mohapatra, Lin Yan Zhou, Kohei Ohta, Takahiro Matsubara, Taisen Iguchi, and Yoshitaka Nagahama

Supplemental Figures

Figure S1

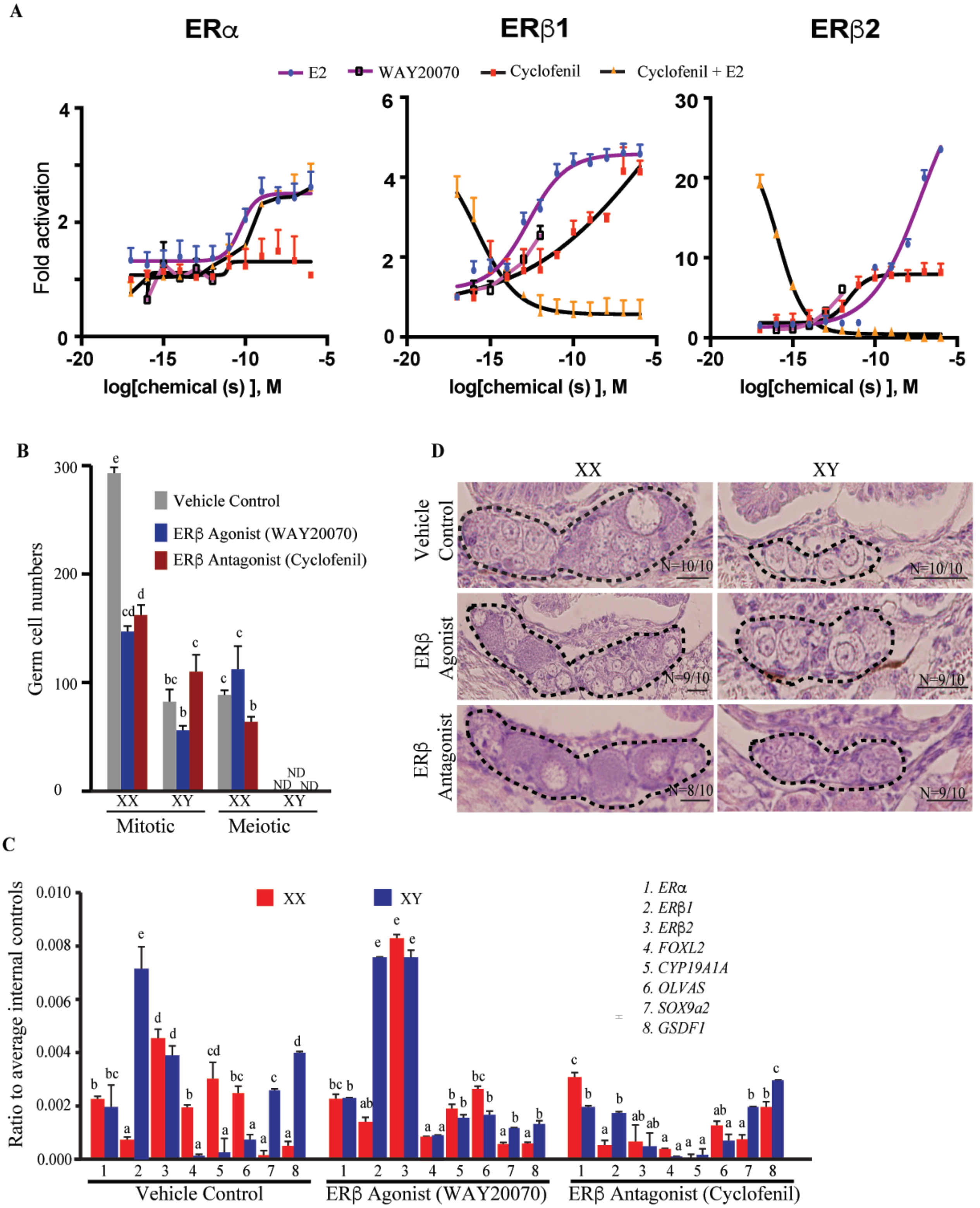

Figure S1. Effect of ERβ agonist and antagonist on medaka estrogen receptor regulation and gonad development. Related to Figure 1. A. Estrogen, ERβ agonist and antagonist regulated *in vitro* transcriptional activity of various medaka estrogen receptors. Transcriptional activity profiles of *ERα*, *ERβ1* and *ERβ2* generated using 17β-estradiol (E_2), WAY20070 (ERβ agonist), Cyclofenil (ERβ antagonist) and Cyclofenil with E_2 depicts differential activation pattern. The experiments were repeated for 6 times and the cumulative data were analysed using Graph Pad Prism software. **B.** ERβ agonist and antagonist treatment influenced the mitotic and meiotic germ cell counts in 10 dah medaka. **C.** Comparative transcriptional profiling of various sexually biased genes was performed to corroborate the changes in germ cell count. **D.** Gonadal histology of different groups depicted no remarkable difference between the experimental groups.

Note: Triplicates, each containing 10 embryos were used for QPCR analysis. 10 XX and 10 XY embryos from each treatment group were separately used for gonadal histology and germ cell counting. In graphs, data are plotted as means ± SEM; different letters denote significant differences at $p<0.01$. Scale bars, 50μm.

Figure S2

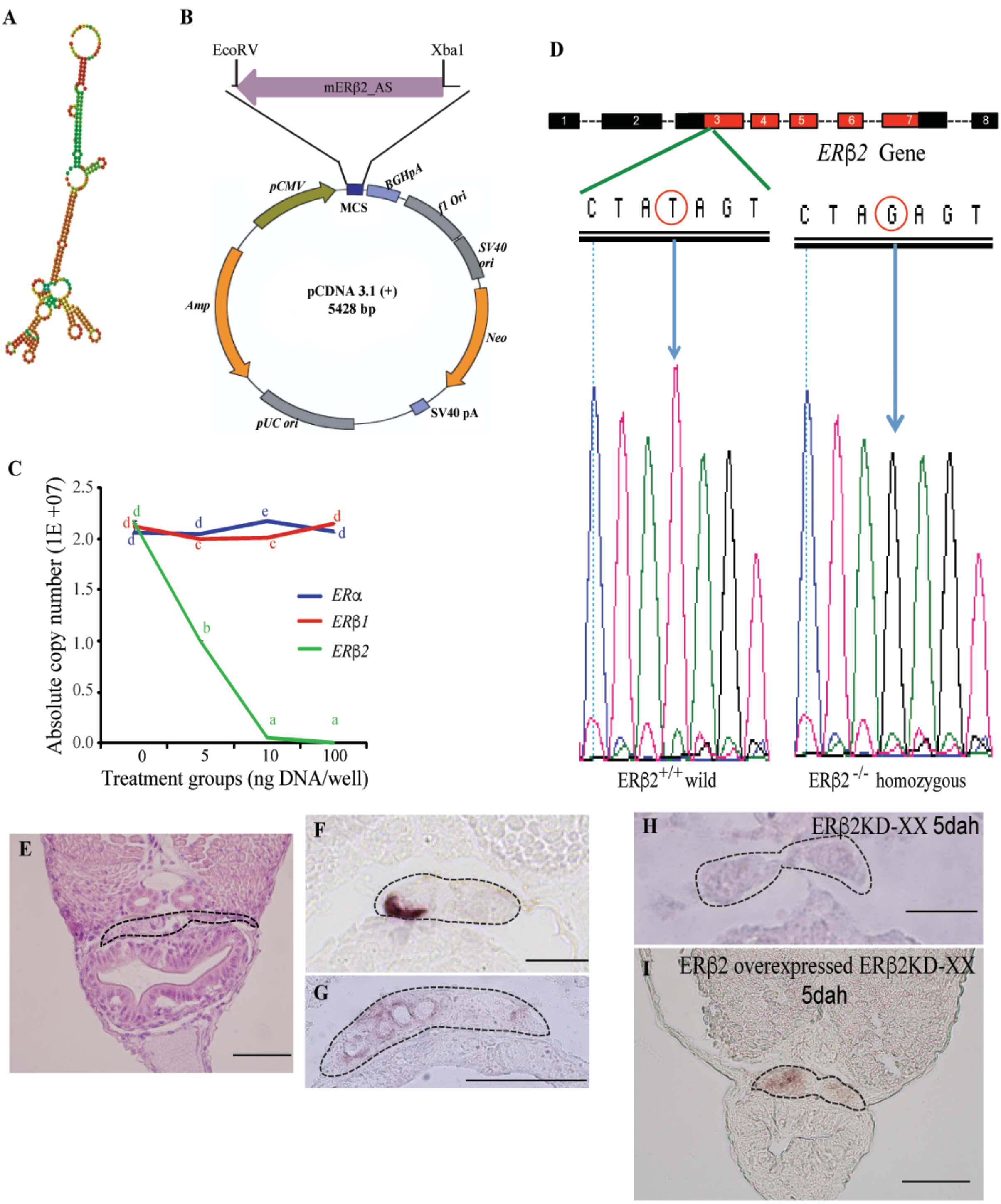

Figure S2. Construction and validation of ERβ2 knockdown/ Knockout. **A-B. Related to Figure 1.** The antisense fragment/probe, predicted to have best secondary AS-RNA structure (A), was inserted in a reverse orientation into the pcDNA 3.1 vector to generate the knockdown plasmid (B). **C.** The efficacy of knockdown construct was determined *in vitro* by co-transfection of *ERα/ERβ1/ERβ2* overexpression plasmids and different concentration of ERβ2-AS plasmid. **D.** Schematic diagram (upper panel) represents the ERβ2 exon (filled box), intron (dotted lines), untranslated regions (black box) and open reading frame (red box) assembly at chromosome 22:23,116,691-23,156,480. The representative sequence chromatograms (lower panel) showing the single nucleotide difference (A > G, marked with red circle and blue arrow) between wild and homozygous ERβ2-KO medaka. **E-G.** Histologically, at 10dah, the ERβ2$^{-/-}$ XX gonad (marked with dotted black boundary) showed fewer occurrences of germ cells and male type gonadal development (E). *In situ* hybridization **(***ISH***)** analysis using *GSDF* (F) and *ERβ2* (G) also indicated gonadal masculinity **H-I.** *ISH* analysis of ERβ2-

KD-XX (H) and *ERβ2* overexpressed ERβ2-KD-XX (I) fish using *SPO11* (meiotic marker) further confirmed the rescuing effect of gonadal feminity. Scale bars, 100μm.

Figure S3

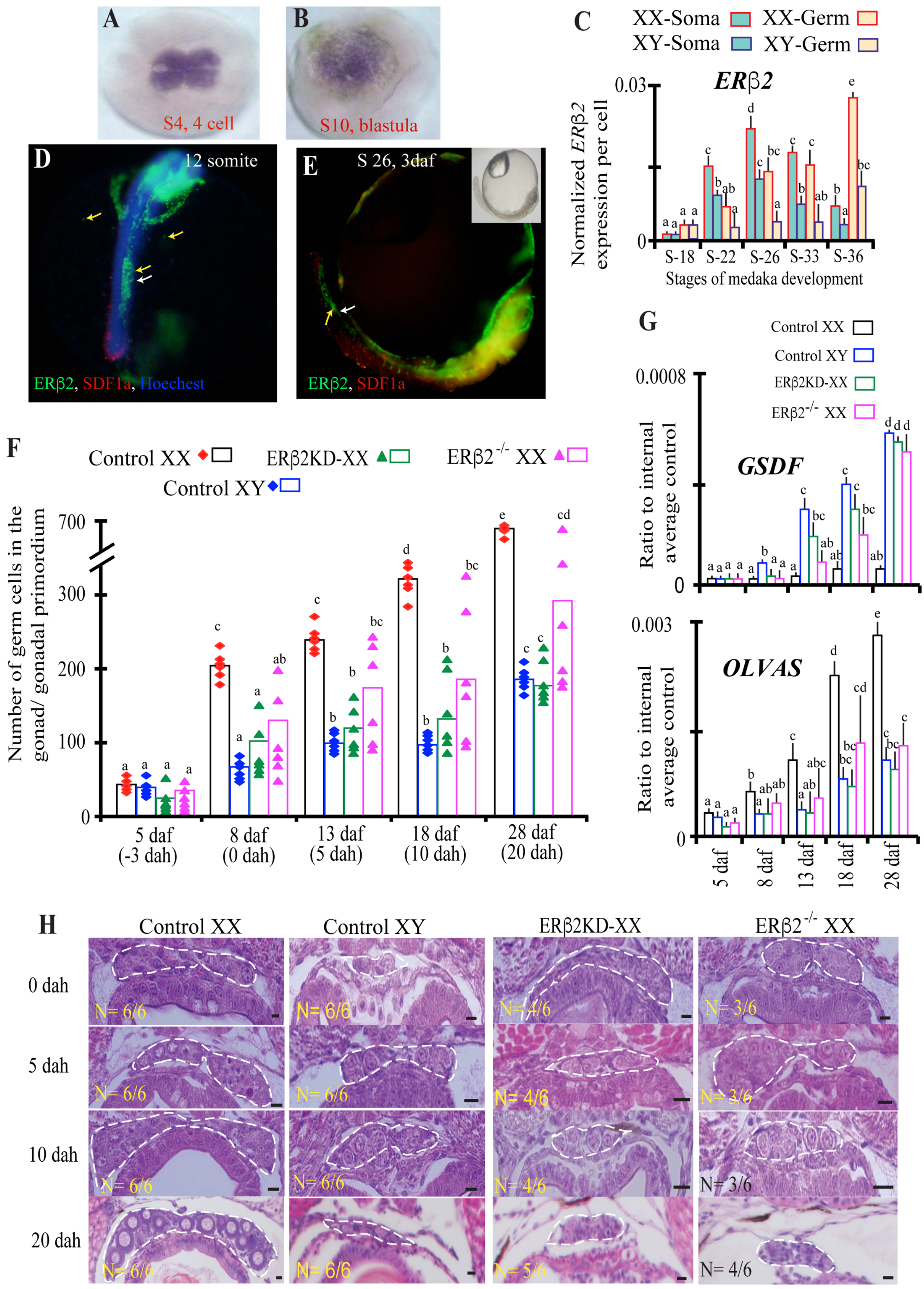

Figure S3: Effect of ERβ2 on early gonadal development and primary sex reversal in medaka. A-B. Related to Figure 1. Wholemount *ISH* (*WISH*) showing the cellular localization of *ERβ2* mRNA in 4 cell (A) and blastula (B) stage

medaka embryo. **C**. *ERβ2* mRNA profiles were analysed using germ and somatic cells sorted from DMY genotyped individual NANOS-dsRED embryos (N= 6 (XY), 8 (XX)), and plotted to determine sex biased *ERβ2* expression profile. **D-E.** Two color fluorescent *WISH* (*FWISH*) using *ERβ2* (green) and *SDF1a* (red) demonstrating their distribution in developing medaka embryos at 12 somite (D) and 3daf (E). The yellow and white arrows respectively denote the representative germ cells and putative gonadal analgen. **F-H.** Assessment of gonadal sexuality and primary sex reversal in ERβ2-KD-XX and ERβ2$^{-/-}$-XX fish. Total germ cell numbers (F) were counted using HE stained serially sectioned samples at 5, 8, 13, 18, and 28daf and individual fish data (marked with triangle, etc) were plotted to ascertain the reversal of gonadal sexuality in both ERβ2-KD and ERβ2$^{-/-}$ XX fish. Simultaneously, the *OLVAS* and *GSDF* mRNA profiles (G) were analysed (N=6 individual) using QPCR to validate the initiation of sex reversal in various experimental groups. Candidate HE stained photomicrographs (H) also demonstrates the varying degree of sex reversal (ERβ2-KD-XX (66.7-83.3%) > ERβ2$^{-/-}$-XX (50-66.7%)) initiated in ERβ2-reduced juvenile gonads before 28daf. Note: In graphs, different letters in small case (a, b, etc.) indicate significant differences at $p<0.05$; Scale bars, 10µm.

Figure S4

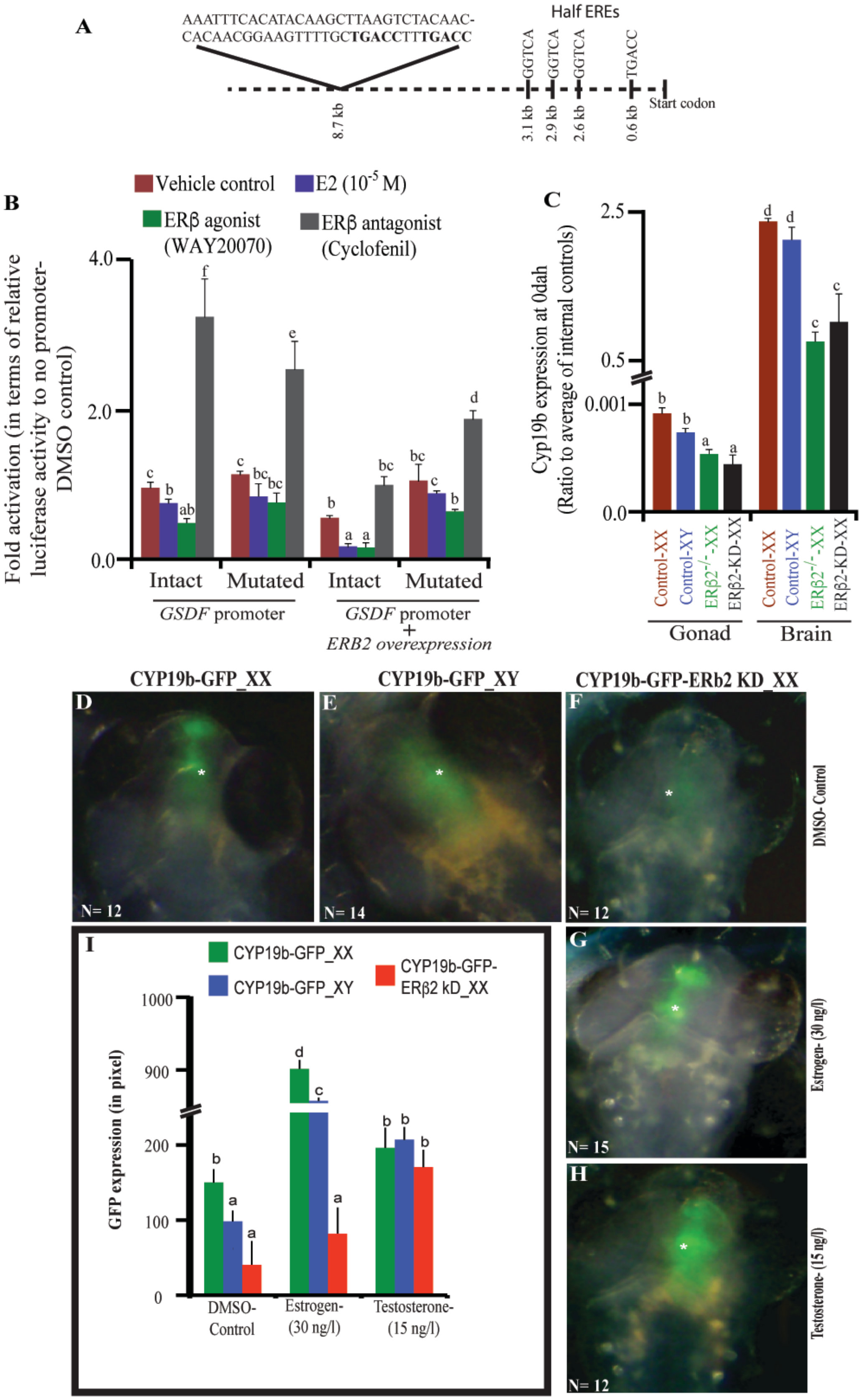

Figure S4. Evaluation of *ERβ2* association with sex biased gene expression. Related to Figure 1 and 3. A-B. 10kb upstream genomic sequences of GSDF start codon (obtained from Ensemble 95, https://asia.ensembl.org/Oryzias_latipes/Transcript/Exons?db=core;g=ENSORLG00000022492;r=12:10454505-10458561;t=ENSORLT00000030736) houses several potential half-EREs (A). Further *in vitro* dual luciferase analysis using both intact and mutated (deleted 60 bp sequence, marked in Figure S4A) and ER agonist, antagonist (with or without ERβ2 overexpression) confirmed ERβ2 associated GSDF promoter suppression. The experiments were repeated for 7

times. **C**. QPCR analysis demonstrating the variable *CYP19b* transcription profiles in brain and gonads of control-XX, control-XY, ERβ2-KD-XX and ERβ2-/- -XX embryos at 0dah. Triplicates, each containing 10 embryonic gonad and brain were used for QPCR analysis. **D-I.** The crossbreeds of ERβ2-KD-CYP19b-GFP-XX females and either of CYP19b-GFP-XY males or ERβ2-KD-XX males were treated with DMSO (vehicle control, C-E), Estrogen (F) and testosterone (G) for 3 hours. The fluorescence intensity of each GFP positive whole brain were captured using Z stack function of confocal microscope (LSM 710, Zeiss, Germany) and subsequently analysed with IMAGE J64 software. The GFP expression (N=11) was plotted on Y-axis and different treatments on X-axis (H). Note: In graphs, data are plotted as means ± SEM; different letters denote significant differences at $p<0.05$.

Figure S5

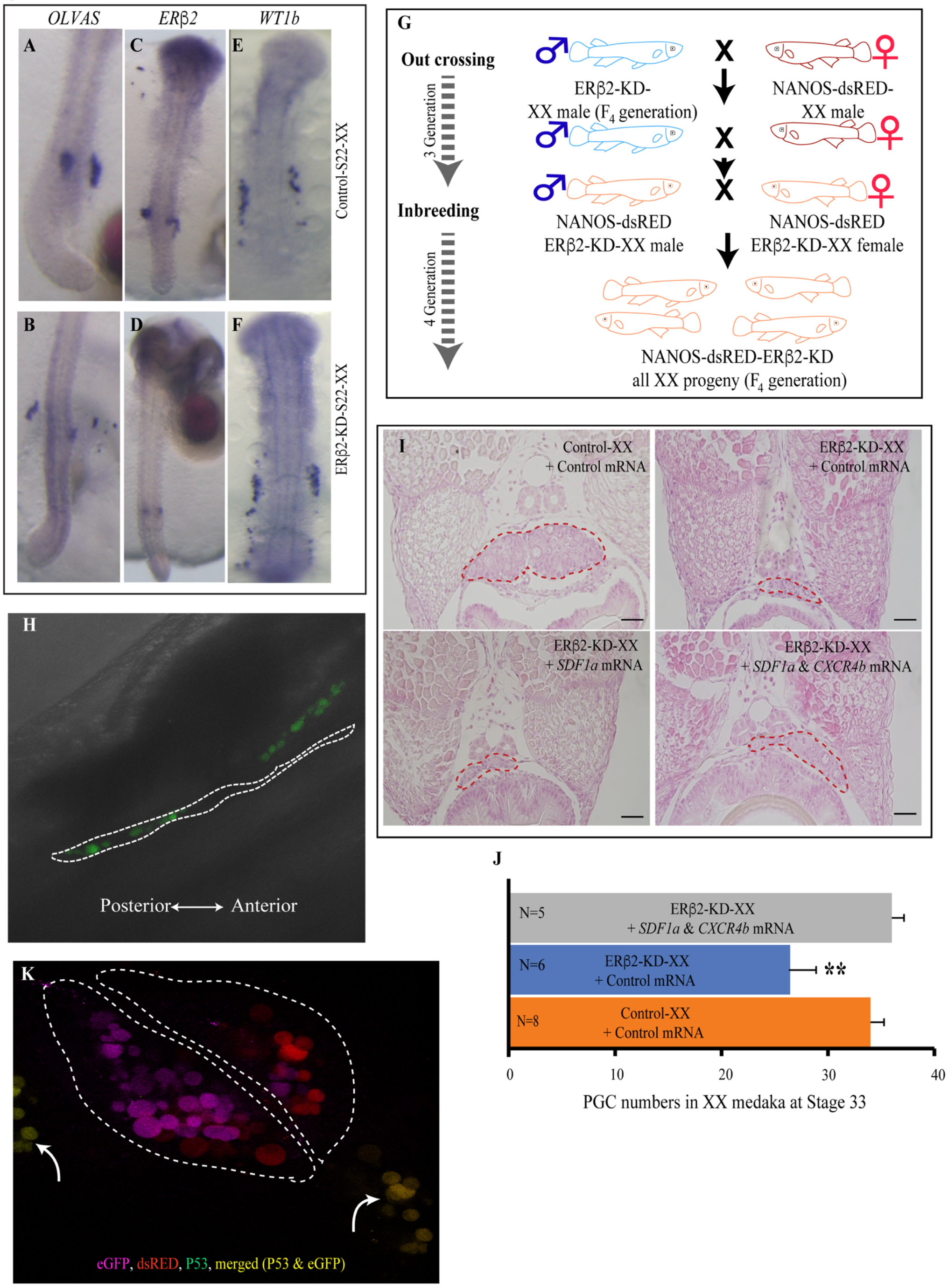

Figure S5. Characteristics of PGCs in ERβ2-KD-XX and ERβ2$^{-/-}$ XX fish. Related to Figure 3. A-F. *WISH* analysis showed abnormal migration of germ cells in ERβ2-KD-XX stage22 medaka embryos (B, D and F) compared to their respective controls (A, C and E). *OLVAS* acted as the germ cell marker, while *WT1b* was used as somatic cells marker in the gonadal primordium. **G.** NANOS-dsRED-ERβ2-KD lines were generated by series of out crossing and inbreeding techniques. Similar methodology was employed to develop OLVAS-eGFP-ERβ2-KD and OLVAS-eGFP-ERβ2-KO (first ERβ2$^{+/-}$ and then ERβ2$^{-/-}$) line. **H.** Similar to ERβ2-KD medaka (see also Figure 3, Table S1), germ cells in homozygous ERβ2-KO medaka failed to localize in the gonadal anlagen (marked with dotted white boundary) at stage22. So, for subsequent analysis we focused on ERβ2-KD medaka. **I-J.** Althogh singular overexpression of either SDF1a or CXCR4b failed to rescue the PGC mismigration, the co-overexpression of *SDF1a* and *CXCR4b* resulted in significantly increased PGC numbers in comparison to the ERβ2-KD-XX fish [See also, Fig. 3G-I and Table S1]. The representative gonadal structures of different overexpressed groups clearly showed that, despite alterations in mitotic germ cell numbers (J), the gonadal size and germ cell count of ERβ2-KD-SDF1a/CXCR4b-overexpressed-XX fish were still less than the Control-XX counterparts (I). Note: In graph, asterisk "*" indicates significant difference at p<0.01. Red dotted line indicates the gonadal boundary. Scale bar, 50μm. **K.** NANOS-dsRED-ERβ2-KD PGCs were transplanted into OLVAS-eGFP host embryos to analyse the *ERβ2* responsive PGC mismigration and death at 10dah (N=10). Note: White dotted line indicates the gonadal boundary and white arrows points out the mismigrated and dying germ cell population.

Figure S6

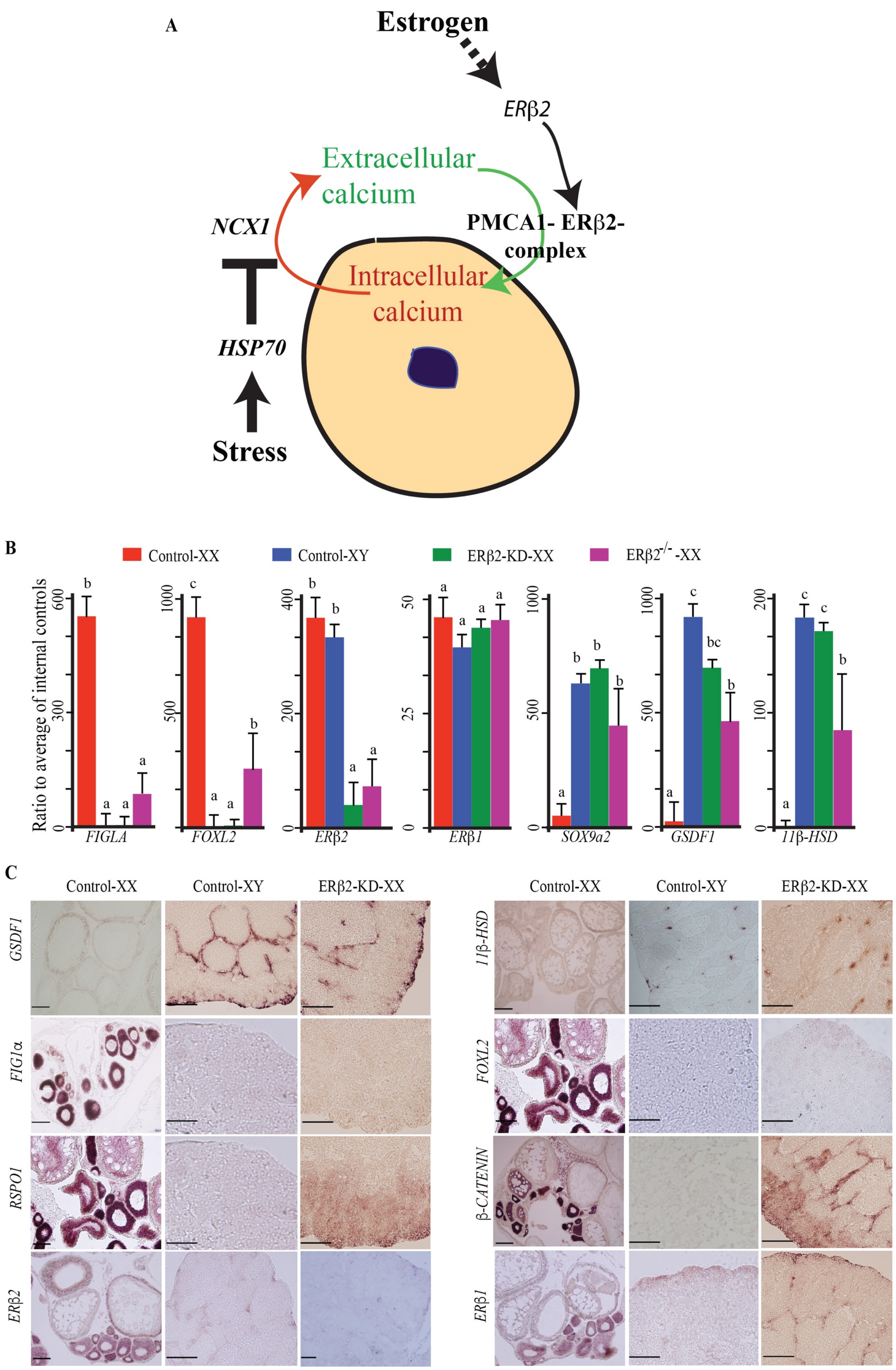

Figure S6. Effect of ERβ2 on calcium ion balance and functional sex reversal. Related to Figure 4 and Figure 5. A. Schematic diagram representing the ERβ2 and calcium ion balance. Estrogen, via ERβ2, regulates the PMCA genes, and

advertently affects the calcium ion transport from the germ cells extracellular to the intracellular region. This change in Ca^{+2} balance in germ cell probably influences the degeneration process of the cell and thereby fuelled functional sex reversal. **B-C.** The transcriptional changes (B) and cellular localization (C) of various sex-biased genes were assessed in the adult Control-XX, Control-XY and ERβ2-KD-XX, and $ER\beta2^{-/-}$ -XX fish to determine the gonadal sexuality and the extent of sex reversal. Note: QPCR analysis were performed using 10 individual samples from each group. Phenotypically male like individuals of ERβ2-KD-XX, and $ER\beta2^{-/-}$ -XX groups were only included in the QPCR and *ISH* analysis. Scale bar, 50μm.

Supplemental Tables

Table S1. Summary of rescuing of PGC migration in ERβ2-KD XX and ERβ2$^{-/-}$-XX medaka. Related to Figure 3.

**Fish group**	**Control-XX-control mRNA over expression**	**ERβ2-KD-XX-control mRNA over expression**	**ERβ2$^{-/-}$-XX-control mRNA over expression**	**ERβ2-KD-XX-*SDF1a* mRNA-over expression**	**ERβ2-KD-XX-*CXCR4b* mRNA-over expression**	**ERβ2-KD-XX-*SDF1a* & *CXCR4b* mRNA-co-over expression**	**ERβ2$^{-/-}$-XX-*SDF1a* & *CXCR4b* mRNA-co-over expression**	**Conrol-XX-*SDF1a* & *CXCR4b* mRNA-co-over expression**
Type of mRNA used	Capped mCherry/Cyan	Capped mCherry/Cyan	Capped mCherry/Cyan	Capped *SDF1a* 5\`utr-orf-mCherry-3\`utr	Capped *CXCR4b* 5\`utr-orf-mCherry-3\`utr	Capped *SDF1a* 5\`utr-orf-mCherry-3\`utr and Capped *CXCR4b* 5\`utr-orf-mCherry-3\`utr	Capped *SDF1a* 5\`utr- orf-mCherry-3\`utr and Capped *CXCR4b* 5\`utr-orf-mCherry-3\`utr	Capped *SDF1a* 5\`utr- orf-mCherry-3\`utr and Capped *CXCR4b* 5\`utr-orf-mCherry-3\`utr
Amount of mRNA used	5 ng each	5 ng each	5 ng each	5 ng each	5 ng each	5 ng each	5 ng each	5 ng each
Number of fish injected	15*	10	7	10	10	15	15	15*
Number of XX fish examined for live germ cell counting at Stage (S)22	7	9	6	10	8	12	11	6
Number of fish examined for live germ cell counting at S33	7	9	6	10	7	12	10	6
Total number of eGFP positive germ cells at S22 (± Standard deviation)	19.7 ± 1.6	18.4 ± 1.4	18.3 ± 1.1	18.0 ± 2.1	18.5 ± 1.5	18.6 ± 1.4	18.9 ± 1.1	19.3 ± 1.4
Average number of germ cell mismigrated at S22 (± Standard deviation)	0.3 ± 0.6	7.3 ± 2.2	3.3 ± 6.2	6.5 ± 1.2	5.4 ± 0.7	1.1 ± 0.5	0.5 ± 0.7	0.3 ± 0.8
Average number of germ cell reached at gonadal primordium at	19.4 ± 1.7	6.7 ± 5.6	9.2 ± 9.8	7.7 ± 5.8	7.0 ± 6.7	14.1 ± 7.1	15.1 ± 7.4	19.0 ± 1.2

S22 (± Standard deviation)								
Total number of eGFP positive germ cells at S33 (± Standard deviation)	38.9 ± 1.7	25.1 ± 1.9	32.1 ± 7.5	30.0 ± 1.3	31.6 ± 1.0	39.7 ± 1.3	39.6 ± 1.2	39.5 ± 1.4
Average number of germ cell mismigrated at S33 (± Standard deviation)	0.7 ± 0.7	11.0 ± 4.2	6.6 ± 6.8	9.0 ± 1.6	9.6 ± 1.4	1.7 ± 1.1	0.9 ± 0.8	0.8 ± 0.6
Average number of germ cell reached at gonadal primordium at S33 (± Standard deviation)	38.1 ± 1.7	8.5 + 8.1	19.8 + 18.2	14.0 ± 10.0	10.3 ± 11.0	30.4 ± 15.3	34.1 ± 11.4	38.7 ± 1.1
Number of fish with meiotic cells in gonad at 10dah (days after hatching)	4 (N=4)	0 (N=6)	2 (N=6)	0 (N=7)	0 (N=4)	8 (N=8)	9 (N=9)	3 (N=3)
Average number of germ cells at 10dah (± Standard deviation)	470.5 ± 17.6 (N=4)	111.5 ± 5.0 (N=6)	179.8 ± 120.5 (N=6)	142.9 ± 8.6 (N=7)	146.8 ± 6.7 (N=4)	252.8 ± 22.2 (N=8)	362.8 ± 20.1 (N=8)	482.5 ± 7.3 (N=3)
ERα mRNA content (N=3) (± Standard deviation)	8.1E-06 ± 3.3E-06	4.2E-06 ± 5.0E-06	4.1E-06 ± 2.5E-06	6.0E-06 ± 8.6E-06	4.0E-06 ± 1.2E-06	7.1E-06 ± 2.5E-06	7.2E-06 ± 1.3E-06	7.1E-06 ± 1.7E-06
ERβ1 mRNA content (N=3) (± Standard deviation)	2.9E-05 ± 3.2E-05	2.4E-05 ± 1.6E-05	2.3E-05 ± 1.8E-05	3.2E-05 ± 9.5E-05	3.2E-05 ± 6.0E-05	2.5E-05 ± 9.1E-06	2.5E-05 ± 4.1E-06	2.5E-05 ± 6.3E-05
ERβ2 mRNA content (N=3) (± Standard deviation)	2.1E-05 ± 5.0E-05	7.6E-06 ± 2.3E-06	7.3E-06 ± 2.1E-06	1.2E-06 ± 6.8E-06	2.4E-06 ± 1.5E-06	8.6E-06 ± 9.0E-06	8.4E-06 ± 3.4E-06	4.7E-05 ± 2.9E -05

Note: Asterisk (*) indicates that control fish were later segregated based on genetic sex.

Table S2. Summary of ERβ2 knockdown in medaka. Related to Figure 1 and Figure 5.

Stage of Sampling (dah; days after hatching)	Analysis method	Number of sample (N)	Number of fish with changes[9]
	**F_0 generation**		
**0dah**	QPCR[1]	10	9
	Histology[2]	30	10
**10dah**	QPCR[1]	10	10
	Histology[2]	35	11
	ISH[3]	10	4
**50dah**	Histology[2]	10	3
**Adult**	Secondary sexual character[4]	12	5
	Breeding behaviour[5]	5	5
	Gene integration[6]	12	3
	**F_1 generation**		
**10dah**	Histology[2]		
	ISH[3]		
**Adult**	Secondary Sexual Characters[4]	50	16
	Genome integration[6]	14	14
	Breeding behaviour[5]	5	3
	**F_2 generation**		
**10dah**	Histology[2]	50	23
	ISH[3]	10	7
**Adult**	Secondary sexual character[4]	80	21
	Breeding behaviour[5]	5	3
	**F_3 generation**		
**2daf**	Germ cell migration[7]	9	9
**10dah**	Histology[2]		
	Rescue with E_2[1,2,3]	20	0
	Rescue with *ERβ* agonist[1,2,3]	20	2
	Rescue with *ERβ2* overexpression[1,2,3]	10	10
	Rescue with Flutamide[1,2,8]	20	20
**Adult**	Secondary sexual character[4]	42	41
	Breeding behaviour[5]	5	4

Note: Asterisk indicates the biological end points analysed using the samples. 1 - Change in gene expression; 2 - Change in germ cell number and occurrence of meiosis; 3 - Changes in cellular localization of different gene; 4 - Occurrence of Fan like anal fin and Forked Dorsal fin; 5 - Mating characteristics, i.e., chasing, dancing, coiling and pressing; 6 - Positive PCR amplification of antisense probe using a vector and gene specific primer pair; 7 - PGC migration and settlement in gonadal primordium; 8 - Changes in eGFP expression; 9 - Deviation from average (± standard deviation, if applicable) control samples, i.e., reduction in gene expression for QPCR, gonad size and germ cell number for histology, intensity (visual observation) and localization of *ISH* staining, variation from normal XX secondary sexual, changes in breeding behaviour, external gene integration, mis-migration (percentile ratio of total germ cell and mis migrated cells >5%) of germ cells, etc.

Table S3. List of major primers used in this study. Related to Supplemental Experimental Procedure

Primer name	Sequence	Peak Standard disscociation (^0C)	Peak amplicon dissociation (^0C)	Geometric amplification efficiency (%)	Purpose
GSDF Realtime F	GGGCTGGACACTATTCGAGA	82.5	82.5	99.11	Measuring Gene expression by QPCR, ORF amplification, *in situ* probe preparation, synthetic RNA preparation, ORF amplification, *in situ* probe preparation
GSDF Realtime R	CATGACACAGAGGAGCTGGA				
SF1 Realtime F	AGCTGCTACTCTGGAAACGA	81.5	81.5	100	
SF1 Realtime R	ACTGGCAATCTTCTTGGCAGC				
GFP Realtime F	CGACAACCACTACCTGAGCA	81.5	81.5	99.23	
GFP Realtime R	GAACTCCAGCAGGACCATGT				
Vasa Realtime F	CCCAAAGTGACCTACATC	81.0	81.0	99.76	
Vasa Realtime R	AAGTTGATGCCCATCTTG				
Foxl2 Realtime F	AAACCTGCTACTCTGGACGC	82.0	82.0	99.45	
Foxl2 Realtime R	AGTCAAATCTTCTTGATTC				
Fig1a Realtime F	TGTACTGCTGCATCGAGAAGTA	82.5	82.5	99.74	
Fig1a Realtime R	ATGCTGCAACACCAGTCTAGT				
Spo11 Realtime F	TCGATTCTGGTGCCGTCTTCT	82.0	82.0	98.96	
Spo11 Realtime R	ATGCTGAAGGTTTCTCGCAGG				
RSPO 1 Realtime F	TGCAACACCAGTCTAATG	81.5	81.5	99.35	
RSPO 1 Realtime R	TTCTGGTGCCGTCTTCTAGG				
Cyp19a1 RT F	AGCTTATTTTTGCCCAAGGCC				
Cyp19a1RT R	TTGAGCAGCAGGAGCATGAAA				
GSDF ORF F	ATGTCTTTGGCACTCATT				
GSDF ORF R	CTACTTTTTGCAGGGCTGCT				
SF1 ORF F	AGCAAGGGTGTGAGGAG				
SF1 ORF R	TTTTTGCAGGGCTGCT				
DMY ORF F	CCGCGGGAGCTCATGAGCAAG GAGAAGCAGTGC				
DMY ORF R	GGATCCGAATTCTGGAGTTGGC CGGGAAGACG				
SPO11 F	GATGCAAGGAGAGAGTT				
SPO11 R	ATACTCAGCTGTTTGGGTCACA				
FOXL2 F	TGCACCTGACACCAGTCT				
FOXl2 R	TAGACAACACCGAGTCTG				
Vasa F	AAGAGCTCCCAGCAAGGC				
Vasa R	TCGGAGCTCATGAGCAAGG				
RSPO F	TGCAACACCAGTCTAATG				
RSPO R	TAGACTGCCCGTCATG				
Fig1a F	ATGAAGGTGCCAGAGGCGGAA T				
Fig1a R	TTAATCCCTCGAAGCTTGATCG				
ERβ2 as Xba1-F1	TCTAGAAGACGAAGGCCAGAT CCT				Amplification of *ERβ2* antisense region1, integration check Colony PCR, Sequencing, Genomic PCR for sorting of sex
ERβ2 as Eco RV R1	GATATCACCCCTGTAAGGTTGT T				
M13 F	GATATCCGAGCATCTCCAGTAG GAGG				
M13 R	TCTAGATCTACAGCATGAAGTG CAA				
T7	GATATCGACCCTCCATACTGAA GGA				
T3	TCTAGACTCTTTGTTCTGGCAA AGCC				
Sp6	GATATCGGTTGCAGGGCAGAT GTAGT				

Bgh R	TCTAGAATGGGAACCACTTTGGACTC
pcDNA 3.1 807 F	GATATCAGCTGAAGATGGTTGGGTTG
PCDNA 3.1 1127 R	ATGAAGGTGCCAGAGGCGGAAT
DMY genomic F	TTAATCCCTCGAAGCTTGATCG
DMY genomic R	TCTAGAAGGAGGAGCTTGGGATTTGT

Supplemental Experimental Procedures

Plasmid constructs

The anti-sense RNA (AS-RNA) expression constructs were designed and evaluated using the online tool E-RNAi (Chakraborty et al., 2016). The sequence was also analyzed for secondary structures *in silico*, using RNA*fold* online software (http://rna.tbi.univie.ac.at/cgi-bin/RNAfold.). A selected primer pair carrying *Xba*I and *EcoR*V sites (Table Supplemental experimental procedure) was used to amplify a 220-bp region of the medaka (m) *ERβ2* gene (Figure S2) from a plasmid containing the complete *ERβ2* ORF (Chakraborty et al., 2011). The amplicon was cloned under the control of the CMV early promoter in pcDNA3.1 (+) (Invitrogen, USA) vector in the antisense orientation. The constructs were transformed into *E. coli*-XL1 blue and the positive clones were confirmed by sequencing (hereafter named as pmERβ2-AS). Similarly, the tet-on vector (Clontech, USA) was used to prepare the conditional ERβ2 knockdown (ERβ2-KD tet-on) construct. Plasmid DNA, used for downstream experiments, was purified using the plasmid purification kit (Qiagen, Germany).

Expression plasmids were constructed using pcDNA3.1(+) vector backbone using complete ORFs of required genes, if not specifically mentioned. pGEMt-easy plasmid of different genes was used for *in situ hybridization* (*ISH*) probe synthesis and QPCR standard preparation, whenever necessary. 4-6 kb long promoter fragment of related genes were isolated using specific primer set, and cloned in pGL3 basic vector (Promega, USA using Infusion-cloning kit (Clonetech, USA). *ERβ2*, *CXCR4b* and *SDF1a* fragments (containing 5'UTR and stop codon less ORFs) were in-framed with mCherry (source: pmCherryN1 vector) and Cyan (source: pamCyanN1 vector) ORF followed by 3` UTR of respective genes and cloned into a pCS2 vector, using Infusion cloning kit (Clontech, USA).

Gene knockdown in HEK-293 cells

Knockdown of *ERβ2* expression by the pmERβ2-AS construct was tested in HEK-293 cells with no endogenous expression of fish *ER*s. HEK-293 cells were seeded in 24-well plates at 5 X 10^5 cells/well in Dulbecco's modified Eagle's Medium (Sigma, USA) supplemented with 10% charcoal/dextran treated fetal bovine serum (Hyclone, USA). The cells were incubated for 24h at 37^0C with 5% CO_2 and co-transfected with either pCMV-mERα/ mERβ1/mERβ2 plasmid (100ng) and 5, 10 and 100ng of the pmERβ2-AS construct using Fugene-6 transfection reagent (Roche Diagnostics, Switzerland) following the manufacturer's instructions. Cells transfected with only pCMV-mER(s) served as positive controls, while the negative controls were treated with only the transfection reagent. The cells were grown for 96h after which total RNA was isolated and target transcripts were quantified by QPCR, as detailed later. The transfections were performed in triplicate at one time point and then repeated twice later. The transcript copy numbers are shown as the mean ± SEM and the dose-response data were computed using Statview v. 5 (SAS institute Inc, Cary NC).

Experimental animals

The QurtE strain of medaka was used for this study, if otherwise not mentioned. This strain expresses a male-specific leucophore, which allows easy sexing of fish. Other strains, such as OLVAS-eGFP, NANOS-dsRED, transgenic medaka were also used. These strains carry the *eGFP/dsRED* gene under *OLVAS* promoter or *NANOS 3'* utr and were reported to express respective fluorescence in germ cells of both males and females (Tanaka et al., 2001; Kurokawa et al., 2006). All the fish were maintained at 26 ± 2^0C under a 14h light and 10h dark cycle. Eggs were collected within 30 min of fertilization and incubated in distilled water (milli-Q), containing antifungal solution (Methylene blue, 0.0001%) at 26 ± 2^0C. Brooders and juveniles were fed with fresh artemia, while larvae were given artificial food. All *in vivo* experiments and fish samplings were conducted following protocols and procedures approved by Institutional Animal care and use committee at the National Institute of Basic Biology, Japan, and Ehime University Animal Use and Ethics Committee,

Japan. Additionally, genetic sex of each individual was examined from fin clips, tail bud, etc., using DMY-genomic PCR (Matsuda et al., 2002), either before pooling samples for subsequent analysis or after live analysis, as per convenience.

Chemicals/steroid treatment

One/two cell staged fertilized medaka embryos (100 embroys/50ml water) were treated till 18 days after fertilization (daf) (if not otherwise specifically mentioned) with Vehicle DMSO/Ethanol (Nacalai Tesque, Japan), ERβ agonist (WAY20070, Tocris bioscience, USA), ERβ antagonist (cyclofenil, Tocris bioscience, USA), 17β-estradiol (E_2) (Sigma, USA), Calcium chloride ($CaCl_2$) (Nacalai Tesque, Japan), extracellular calcium chelator (EGTA, Sigma, USA), intracellular calcium chelator (BAPTA_AM, Sigma, USA), and Doxycycline (Gibco, USA). The embryos were reared as mentioned above with daily water exchange.

Histology, *in situ hybridization* (*ISH*), wholemount *ISH* (*WISH*), fluorescent *WISH* (*FWISH*), and fluorescent immunohistochemistry (FIHC)

Bouin fixed, paraffin embedded samples were used for standard Hematoxylin & Eosin (HE) staining, while, 4% paraformaldehyde fixed samples were used for *ISH, WISH,* and *FWISH.* All the histological analyses were performed using 5µm sections. For *ISH*, sense and anti-sense digoxigenin-labelled RNA probes were transcribed *in vitro*, using RNA labelling kit (Roche Diagnostics GmbH, Germany), from plasmid DNA containing the respective genes. Sections were deparaffinised, hydrated, treated with proteinase K at 10µg/ml (Roche), and hybridized with the sense or anti-sense labelled RNA probes at 58^0C for 18-24 h. The hybridization signals were detected following previously published protocols (Chakraborty et al., 2011). *WISH* was performed using previously published protocols (Mohapatra et al., 2015). The FIHC procedures are same as previously described (Inoue and Wittbrodt, 2011). Briefly, PFA fixed samples were treated for antigen retrieval, peroxidase activity and blocked in 5% FBS-PBS, incubated with primary (1:10000 dilution) and Alexa tagged secondary (1:20000 dilution) antibody, and micro graphed using confocal microscope (LSM 710, Zeiss, Germany). LC3 (GeneTex, GTX127375) and OLVAS (Genetex, GTX128306) antibodies were used in our study.

Quantification of changes in gene expression by QPCR

Changes in gene expression were quantified using the ABI Prism 7000 sequence detection system (Applied Biosystems, USA). Total RNA was isolated from cells, embryos or gonads using RNeasy Mini kit (Qiagen, Germany). cDNA synthesis was carried out using a Quantitect RT PCR kit (Qiagen) from 100 ng of total RNA. The first strand cDNAs were diluted to 100µl for subsequent use. Gene-specific QPCR was performed using SYBR green master mix (Applied Biosystem) and 5 ng of cDNA, according to the manufacturer's instructions. The PCR conditions included an initial denaturation at 94^0C (2 min) followed by 40 cycles at 94^0C (30 s) and 60^0C (1 min). *Ef1α* and *rps18* were used as the internal controls. The absolute transcript copy number of each gene was determined with the help of appropriate standard curves and normalized with the average of *ef1α* and *rps18* copy numbers in each sample. The reported values are averaged from experimental triplicates, if not otherwise mentioned. The specificity of primer sets, throughout this range of detection, was confirmed by the observation of a single amplification product of the expected size, melting curve, Tm (melting temperature) and sequences. All assays were quantified, with standard curves (mean Ct vs. log cDNA dilution) having slopes between -2.99 and -3.34, a linear correlation (R^2) between the mean Ct and the logarithm of cDNA dilution of >0.985 in each case. All test cDNAs were run in duplicates for each gene. The primers were designed according to unmutated (wild) DNA sequences, if not otherwise mentioned.

Promoter analysis

4-6 kb promoters of *WT1b*, *SDF1a*, and *CXCR4b* were isolated from medaka genomic DNA and directionally cloned in pGL3 promoterless luciferase vector. The promoter analysis was performed using previously described protocol (Chakraborty et al., 2011). Briefly, HEK-293 cells were seeded in 24-well plates at 5 X 10^5 cells/well in Dulbecco's modified Eagle's Medium (Sigma, USA). After 24h, the cells were transfected with pGL3-promoter-luciferase plasmid and pcDNA3.1-ERβ2 plasmid at different concentration (0-200ng/well) in triplicates. The luciferase assay was performed after 48 hours of transfection. The experiment was repeated thrice for reproducibility.

Knockdown of ERβ2 expression in medaka embryos

One-two cell embryos of the QurtE strain of medaka were electroporated with the pmERβ2-AS plasmid construct (1µg/ml) in 1X HBS buffer, pH 7.53 (Chakraborty et al., 2016). Electroporation was carried out using 4mm gap cuvette (BTX, USA) in a Cuy21 edit type electroporator (Bex, Tokyo) at 24V, with a 9 millisecond (ms) pulse duration, a 900 ms pulse interval and 3 pulses. Embryos electroporated with only HEPES buffered saline (HBS) served as control. No significant difference in lethality was observed between pmERβ2-AS (7.1±3.5/200 fertilized egg), empty vector (6.8±3.6/200 fertilized egg) electroporated and non-electroporated (7.2±3.1/200 fertilized egg) control. DNA was extracted from 10 randomly collected individuals and plasmid DNA was PCR amplified and sequenced to confirm the electroporation. Importantly, 9/10 pmERβ2-AS and 10/10 empty vector electroporated embryonic DNA showed PCR amplification, suggesting higher incorporation rate.

Electroporated female embryos of the QurtE strain of medaka were sampled at 0, 10, 50 and 120 (adult) days after hatching (dah) and to check the morphological changes in gonad and confirm gene knockdown at the genetic and phenotypic level. At least, 10 fish were sampled at each time point. The genetic sex of all fish was confirmed by dmy-genomic PCR (Matsuda et al., 2002).

Assessment of the transgenerational knockdown effect

The QurtE embryos injected with pmERβ2-AS were grown to adulthood. Based on integration PCR, genetic sexing, secondary sexual characters and the absence of leucophores, genetic females (sex reversed to males) were identified and outcrossed with normal females to assess their breeding behavior and mating performance (Table S2). Successfully mated XX males were used to produce F_1 progeny. Caudal fin clips from XX males of the F_0 and F_1 generations were used for genomic DNA isolation (Matsuda et al., 2002). The genome integration of the pmERβ2-AS construct was tested using a primer pair that amplified a portion of the vector and *ERβ2* antisense sequence (Table S1), followed by sequencing. Aliquots of the same genomic DNA samples were analyzed by QPCR to estimate the number of integrated copies. The breeding experiment was similarly performed for F_1 and F_2 fish and the offsprings were histologically examined at 10 and 20dah (Table S2). All other experiments were conducted using F_4 or later generation fish.

Rescue of PGC migration

To rescue the *ERβ2* knockdown effect on PGC migration, the F_4 generation fish developed from OLVAS-eGFP-ERβ2-KD-XX line were used. Briefly, *sDF1a*-Cyan and *cxcr4b*-mCherry mRNA were synthesized with mMESSAGE mMECHINE SP6 kit (Ambion), following polyA addition with Poly-A tailing kit (Ambion). The purified RNA(s) were injected in one/two cell stage embryos of OLVAS-eGFP-ERβ2-KD-XX fish @ 1ng/µl. Each embryos were monitored at stage 15, 22, 27 and 32, using confocal microscope (LSM 710, Zeiss, Germany). The Z-stage confocal sections of each live embryo were further analysed to ascertain the PGC numbers at each stage. The embryos showing reporter gene expression were then grown separately until 10dah on a 24 well dish @ 1 embryo/well and fixed/preserved for histological/QPCR analysis.

Chromatin immunoprecipitation (ChIP)

Atleast 25Kb upstream sequence (from the ORF start site) were obtained from ensemble (http://asia.ensembl.org/index.html), and the promoter regions were identified using promoter 2.0 prediction server (http://www.cbs.dtu.dk/services/Promoter/). 10Kb genomic region, including the promoter region, was further analyzed with Dragon ERE finder version 3.0 (http://datam.i2r.a-star.edu.sg/ereV3/), and two most potential ERE sites were predicted. For *in vivo* ChIP analysis, the ChIP expression-shearing kit (Active motif) was used according to the manufacturer's instructions, using 20mg of XX or XY embryonic samples (10 fish each), and ERβ antibody (3mg, Active motif, 39767). Preliminary experiments were conducted with 20mg of embryonic samples from ERβ2-eGFP mRNA injected fish and eGFP monoclonal antibody (3mg, AbCAM, ab184601), to validate the ER specificity. The ERβ2-eGFP plasmids was constructed by sequentially fusing *ERβ2*-5`UTR, *ERβ2*-ORF (stop codon less), *eGFP* and *ERβ2*-3`UTR into a pCS2 vector, using infusing cloning kit (Clonetech). RNA was synthesized with mMESSAGE mMECHINE SP6 kit (Ambion, USA) following polyA addition using Poly A tailing kit (Ambion). After tissue disaggregation and cell re-suspension, DNA was sheared, according to the manufacturer's protocols. The ChIP procedure using ERβ2-eGFP mRNA was validated as described earlier (Chakraborty et al., 2016).

Germ cell transplantation

NANOS-dsRED-ERβ2-KD-XX stage 22 embryos were dissociated using Ringer's solution containing 1% citric acid trisodium and 0.1% trypsin (Defco) for 30 minutes and the single cell suspensions were filtered using 40mm cell strainer. The single cell suspensions were transferred onto a 120 mm glass dish filled with Ringer solution, supplemented with 5% FBS, 0.01% penicillin and 0.01% streptomycin. dsRED-PGCs were aspirated into a glass microneedle under a fluorescence stereomicroscope and 5-6 PGCs were transplanted into the marginal region of the blastodisc of Stage 10 dechorionated OLVAS-eGFP embryo. Single embryos were incubated in 96 well flat bottom culture dish with daily water exchange and hatched embryos were individually cultured till 10dah. Thereafter, the embryos were fixed in 4% paraformaldehyde and multicolor IHC was performed, using eGFP, dsRED and P53 antibodies.

Flow cytometry and cell sorting

Each embryo (from different groups), following sexing, were incubated in L15 media containing 0.25% Trypsin for atleast 30 min, with periodical shaking, to prepare a single cell suspension. The enzymatic digestion was stopped using 2% FBS and the cell suspension was immediately fixed with 2% formaldehyde-PBS for 15 min at 4^0C, washed 3 times with PBS and re suspended in blocking solution (PBS containing 2% FBS and 0.001% Triton X). One hour after incubation the cells were pelleted, incubated with Alexa-tagged antibodies for 1h, washed several times with PBST (PBS + 0.01% tween 20), and subjected to cell sorting using Cell Sorter SH800 (Sony, Japan), following pre-adjusted protocols of germ cell sorting. The required group of sorted cells (cells from atleast 1000 embryos) were collected (if not otherwise mentioned), and RNA was isolated using RNeasy FFPE kit (Qiagen, Japan), following manufacturer's protocol (if not specifically mentioned otherwise). The cDNA synthesis and QPCR was performed as described above. The GFP (MBL, D153-A48) and LC3 (GeneTex, GTX127375) antibodies used in our experiments.

Data analysis

All experiments were conducted for a minimum of three times (biological replicates) and statistical differences were assessed based on biological replicates, if not otherwise mentioned. Statistical differences in relative mRNA expression between various experimental groups were assessed by One or two-way ANOVA of normalized data, followed by

Tukey's test, or Student's t-test. All statistical analyses were performed using SPSS, version 22. All experimental data are shown as mean ± SEM. Differences were considered statistically significant at $p<0.05$, if not otherwise mentioned. The correlations were calculated using Pearson correlation coefficient method.

Supplemental References

Chakraborty, T., Shibata, Y., Zhou, L.Y., Katsu, Y., Iguchi, T., and Nagahama, Y. (2011). Differential expression of three estrogen receptor subtype mRNAs in gonads and liver from embryos to adults of the medaka, *Oryzias latipes*. Mol. Cell. Endocrinol. *333*, 47-54.

Chakraborty, T., Zhou, L.Y., Chaudhari, A., Iguchi, T., and Nagahama, Y. (2016). *Dmy* initiates masculinity by altering *Gsdf/Sox9a2/Rspo1* expression in medaka (*Oryzias latipes*). Sci. Rep. *6*, 19480.

Inoue, D., and Wittbrodt, J. (2011). One for all-a highly efficient and versatile method for fluorescent immunostaining in fish embryos. PLoS One 6:e19713.

Kurokawa, H., Aoki, Y., Nakamura, S., Ebe, Y., Kobayahi, D., and Tanaka, M. (2006). Time-lapse analysis reveals different modes of primordial germ cell migration in the medaka *Oryzias latipes.* Dev. Growth Differ. *48*, 209- 221.

Matsuda, M., Nagahama, Y., Shinomiya, A., Sato, T., Matsuda, C., Kobayashi, T., Morrey, C.E., Shibata, N., Asakawa, S., Shimizu, N., et al. (2002). DMY is a Y-specific DM-domain gene required for male development in the medaka fish. Nature *417*, 559-563.

Mohapatra, S., Chakraborty, T., Miyagawa, S., Zhou, L.Y., Ohta, K., Iguchi, T., and Nagahama, Y. (2015). Steroid responsive regulation of IFNg alternative splicing and its possible role in germ cell proliferation in medaka. Mol. Cell. Endocrinol. 400, 61-70.

Tanaka, M., Kinoshita, M., Kobayashi, D., and Nagahama, Y., (2001). Establishment of medaka (*Oryzias latipes*) transgenic lines with the expression of green fluorescent protein fluorescence exclusively in germ cells: A useful model to monitor germ cells in a live vertebrate. Proc. Natl. Acad. Sci. USA *98*, 2544-2549.
