# Supplementary material for: Estrogen Receptor β2 Oversees Germ Cell Maintenance and Gonadal Sex Differentiation in Medaka, Oryzias latipes
Source: Stem Cell Reports. 2019 Aug 13;13(2):419–33. doi: 10.1016/j.stemcr.2019.07.013 (PMC6700524; doi:10.1016/j.stemcr.2019.07.013)
Supplement: Document S2. Article plus Supplemental Information [file mmc2.pdf]

Stem Cell Reports
Article

ISSCR

OPEN ACCESS

Estrogen Receptor β2 Oversees Germ Cell Maintenance and Gonadal Sex Differentiation in Medaka, *Oryzias latipes*

Tapas Chakraborty,[1,2,5,*] Sipra Mohapatra,[1] Lin Yan Zhou,[2,3] Kohei Ohta,[2,4] Takahiro Matsubara,[1] Taisen Iguchi,[5,6] and Yoshitaka Nagahama[1,2]
[1]South Ehime Fisheries Research Center, Ehime University, Ainan 798-4206, Japan
[2]Laboratory of Reproductive Biology, National Institute for Basic Biology, Okazaki 444-8585, Japan
[3]Key Laboratory of Aquatic Science of Chongqing, School of Life Science, Southwest University, Chongqing 400715, China
[4]Laboratory of Marine Biology, Kyushu University, Fukuoka 812-8581, Japan
[5]Laboratory of Molecular Environmental Endocrinology, Okazaki Institute for Integrative Bioscience, National Institute of Natural Sciences, Okazaki 444-8787, Japan
[6]Nanobioscience, Yokohama City University, Yokohama 236-0027, Japan
*Correspondence: tchakraborty83@gmail.com
https://doi.org/10.1016/j.stemcr.2019.07.013

SUMMARY

In vertebrates, estrogen receptors are essential for estrogen-associated early gonadal sex development. Our previous studies revealed sexual dimorphic expression of estrogen receptor β2 (ERβ2) during embryogenesis of medaka, and here we investigated the functional importance of ERβ2 in female gonad development and maintenance using a transgenerational ERβ2-knockdown (ERβ2-KD) line and ERβ2-null mutants. We found that *ERβ2* reduction favored male-biased gene transcription, suppressed female-responsive gene expression, and affected *SDF1a* and *CXCR4b* co-assisted chemotactic primordial germ cell (PGC) migration. Co-overexpression of *SDF1a* and *CXXR4b* restored the ERβ2-KD/KO associated PGC mismigration. Further analysis confirmed that curtailment of ERβ2 increased intracellular Ca^{2+} concentration, disrupted intra- and extracellular calcium homeostasis, and instigated autophagic germ cell degradation and germ cell loss, which in some cases ultimately affected the XX female sexual development. This study is expected improve our understanding of germ cell maintenance and sex spectrum, and hence open new avenues for reproductive disorder management.

INTRODUCTION

Sex steroids and their receptors harmoniously maintain the reproductive physiology, and any disruption in sexual development leads to a huge impact on individual or species physiology (Windsor et al., 2018). The action of the female hormone, estrogen, is mostly mediated by estrogen receptors (ERs), whose action on sterility, infertility, or subfertility and sexual behavior have been thoroughly investigated using selective ER modulators, *ERα*- and *ERβ*-knockout (KO) mice (Bondesson et al., 2015; Chen et al., 2009; Dupont et al., 2000), and *ERα*-, *ERβ1*-, and *ERβ2*-KO zebrafish (Lu et al., 2017). Although *ERβ*s have recently gained popularity in vertebrate folliculogenesis and infertility studies (Antal et al., 2012; Khattri et al., 2009; Lu et al., 2017), their involvement in early gonadal sex differentiation and maintenance requires further investigation.

Germ and somatic cells are two essential variables in gonadal differentiation and sexual identity in mouse, chicken, and fish (DeFalco and Capel, 2009). In vertebrates, primordial germ cells (PGCs) arise at a distant site, divide, migrate through the gut mesentery and bloodstream, and arrive at and colonize at the gonadal primordium during the bipotential stage (DeFalco and Capel, 2009). During early embryonic development, PGCs migrate to the newly formed gonadal anlagen with the help of germ-soma chemotaxis. Notably, cellular chemotaxis is affected by external steroids (Bondesson et al., 2015), ERs (Gamba et al., 2010), and calcium homeostasis (Wu et al., 2009). More or less at the same time, the somatic cells undergo a series of genetic sex- or environmental-dependent modifications and, along with germ cell, decide the gonadal sexuality (DeFalco and Capel, 2009). Furthermore, migrational disruption and depletion of PGC number causes suboptimal PGC settlement in the gonadal primordium and, in turn, blocks female fate and sometimes triggers male development (Tzung et al., 2015). In medaka, an important model species for gonadal sex determination and differentiation studies (Matsuda et al., 2002), the early gonadal settlement of PGCs is regulated by SDF1/CXCR4-mediated chemotactic migration (Herpin et al., 2008; Kurokawa et al., 2007). Later, during medaka sex differentiation, both germ and somatic cells co-operatively regulate the gonadal development in an estrogen-dependent manner (Kurokawa et al., 2007) and help the PGCs to undergo a proliferative mitosis and meiosis in females, while restricting proliferation in males.

Estrogen is known to affect the transcriptional profiles of several major sex-related genes (e.g., *DMRT1*, *GSDF*, Aromatase, *RSPO1*), germ cell proliferation characteristics, and sexual identity in medaka (Chakraborty et al., 2011, 2016; Okubo et al., 2011; Shibata et al., 2010; Zhou et al., 2016). Earlier we found that, during early sex differentiation, *ERβ2* predominantly expresses in the germ cells of

This is an open access article under the CC BY license (http://creativecommons.org/licenses/by/4.0/).

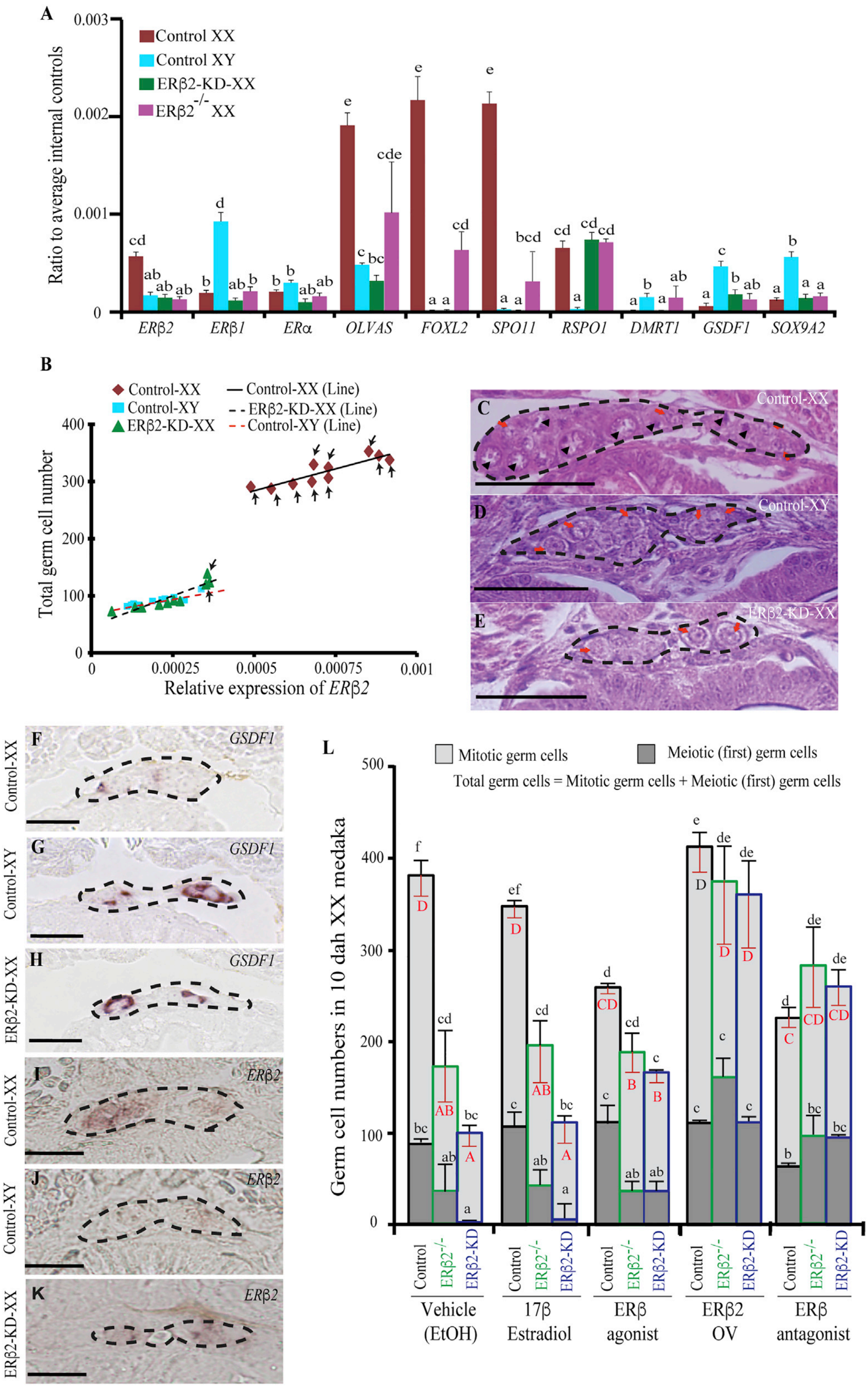

(legend on next page)

embryonic female gonad, while estrogen treatment specifically induces *ERβ2* expression in XY male fish, and thereby speculated the *ERβ2* association in germ cell maintenance (Chakraborty et al., 2011).

This study was conducted to determine the specific roles of *ERβ2* in early germ cell development and its consequences on sexual identity. In brief, we have developed transgenic *ERβ2* knockdown (ERβ2-KD) and knockout (ERβ2-KO) medaka lines and assessed the *ERβ2*-mediated effects on sex differentiation/maintenance and sex reversal. Furthermore, we have ascertained the direct involvement of *ERβ2* in SDF1/CXCR4-mediated chemotactic PGC migration, and calcium homeostasis-related germ cell survival and death, which, in some cases, eventually affects the seeding population of germ cells in the gonadal anlagen and disrupts normal sexual development.

RESULTS

Germ Cell Proliferation Is Associated with *ERβs*

To ascertain the role of *ERβ*s in gonadal development *in vivo*, we treated fertilized embryos with an ERβ-specific agonist (WAY20070, 1 nM), antagonist (cyclofenil, 10 nM), or ethanol (vehicle control) for a period of 18 days (until 10 days after hatching [dah]). Histological analysis depicted a significant reduction in gonad size and germ cell numbers in both agonist- and antagonist-treated fish (Figure S1). Although meiotic cell numbers were slightly higher in agonist-treated fish, the mitotic proliferation reduced drastically in the ERβ agonist-treated than in the ERβ antagonist-treated XX fish. Previously, based on E_2-dependent *ERβ1* and *ERβ2* expression profiles in medaka embryos, we hypothesized that both these ERβ subtypes work, respectively, on "cessation of male germ cell proliferation" and "mitotic burst in female." Thus, the present agonist and antagonist (both *in vivo* and *in vitro*; Figure S1) treatments further corroborate that *ERβ1* and *ERβ2* has an antagonistic role in medaka (Chakraborty et al., 2011). Even though histologically no significant phenotypic changes were observed, ERβ agonist selectively reduced the male-dominated genes, i.e., *SOX9a2* and *GSDF*, in the XY fish, while the female-dominated genes (*FOXL2* and *CYP19a1*) were found to decrease in XX females upon antagonist treatment (Figure S1). Interestingly, the correlation ($p < 0.05$) between histological and transcriptional changes was much higher for ERβ2 (correlation coefficient [CR] = 0.78, n = 15) than ERβ1 (CR = 0.27, n = 15), thus highlighting the importance of *ERβ2* in early medaka gonadogenesis (Chakraborty et al., 2011). This finding is supported by our previous report wherein *ERβ2* showed female-dominated expression in the early sex determination period (Chakraborty et al., 2011).

ERβ2-KD Restricts Germ Cell Proliferation in Embryonic Medaka Gonad

To determine the importance of *ERβ2* in estrogen-dependent sex differentiation of medaka, we knocked down the *ERβ2* expression (Figure S2) in 1- to -2 cell stage embryo, using a pre-established transgenerational knockdown technology (Chakraborty et al., 2016). We simultaneously scouted the medaka tilling mutant library and generated ERβ2-KO line (Figures S2 and S3). *ERβ2* reduction resulted in an average decrease of *ERβ2* transcript of 67% (66%–80% in females [Figure 1A] and 41%–69% in males). Interestingly, the germ cell number and corresponding *OLVAS* (vasa homolog and medaka germ cell marker) expression (Figures 1B and S3) showed a remarkable direct

Figure 1. Effects of ERβ2 Reduction on Gonadal Sex Differentiation of XX Medaka at 10 Days after Hatching

(A) qPCR (n = 10 pooled samples/group; each pool contains 10 randomly collected individuals) analysis of several sex-specific genes depicted a male-biased transcriptional profile of ERβ2-KD and -KO fish.

(B) Germ cell numbers showed a strong correlation with *ERβ2* expression. Chronologically, gonadal germ cell population was determined by confocal imaging, *ERβ2* concentration was measured by qPCR in the same OLVAS-eGFP-ERβ2-KD embryos, and later general linear modeling was used for statistical analysis (n = 10 individual samples/group). The individual gonads that housed the meiotic cells are marked with black arrows.

(C–E) Proliferative mitosis and meiosis was evident in control-XX fish (C), while control-XY (D) and ERβ2-KD-XX (E) fish demonstrated male-type gonadal development, characterized by mitotic and meiotic blockage.

(F–L) *In situ* hybridization analysis using *GSDF* (F–H) and *ERβ2* (I–K) confirmed the gonadal masculinity. (L) Furthermore, different embryonic treatments, i.e., 17β-estradiol (E_2, 1 ng/L), *ERβ* agonist (WAY20070, 1 nM), *ERβ* antagonist (Cyclofenil, 10 nM), and *ERβ2* overexpression, were performed using ERβ2-KD-XX and control-XX embryos to rescue the masculine effect of ERβ2-KD. Ethanol (EtOH)-treated samples were used as vehicle control. In graphs, data are plotted as means ± SEM; different letters denote significant differences at $p < 0.01$. In (L), letters in lower case (a–f) and upper case (A–D), respectively, indicate significant differences among mitotic and meiotic cell population (black, continuous error bars) and total cell population (red, continuous inverted error bars) at $p < 0.01$. Red arrows indicate candidate mitotic cells while black arrowheads denote the cells undergoing first meiosis. Black dotted lines mark the gonadal boundary. n = 10 fish used for each experiment per group.

Scale bars, 100 μm. See also Figures S1–S3 and Table S2.

correlation with *ERβ2* mRNA expression (CR XX = 0.89, CR XY = 0.91, and CR ERβ2-KD-XX = 0.9; $p < 0.01$). Live confocal imaging and subsequent qPCR analysis of OLVAS-eGFP-ERβ2-KD and control OLVAS-eGFP embryos (Figures 1C–1E), and *in situ* hybridization (ISH) (Figures 1F–1K) demonstrated that ERβ2 reduction not only increased the GSDF abundance in gonadal primordium but also significantly suppressed the mitotic and meiotic germ cell count. Notably, at 10 dah, in control female gonad germ cells undergo rapid mitotic and meiotic proliferation (Figure 1C), while males possess mitotically quiescent sporadically distributed germ cells in the gonad (Figure 1D), resulting in differences among germ cell numbers of both sexes. The ERβ2-KD-XX and ERβ2$^{-/-}$XX fish gonads showed more similarity toward males than females (Figure 1E) and also harbored fewer germ cells in the gonad (Figure 1L). Several authors have suggested that *GSDF* negatively regulates the initiation of meiosis in medaka (Gautier et al., 2011; Shibata et al., 2010), probably by influencing the germ-somatic cell interaction. *In silico* analysis depicted several half-ERE in the *GSDF* promoter sequence, making it a potential target for ERs, and *in vitro* analysis confirmed the *ERβ2*-responsive GSDF promoter activity (Figure S4). qPCR analysis demonstrated a significant increase in *DMRT1* and *GSDF* expression, and simultaneous reduction in several ovarian responsive genes, i.e., *SPO11* and *FOXL2* (Figure 1A) in ERβ2-KD-XX fish. Additionally, we observed sex-biased, but relatively ubiquitous, *ERβ2* expression in germ and various somatic cells during PGC migration and gonadogenesis (Figure S3), which suggests that the action of *ERβ2* might be associated with *GSDF* during gonadal sex differentiation. However, interaction with other ovary-responsive GSDF-linked genes cannot be ruled out.

Our recent data highlight that *RSPO1*, an estrogen-responsive gene, regulates the *GSDF* expression (Chakraborty et al., 2016; Zhou et al., 2016). The unchanged *RSPO1* expression in the ERβ2-KD XX embryos suggests some unknown intricate connections between *RSPO1*, *ERβ2*, and *GSDF* in medaka gonad. Although we observed a male-specific gene expression pattern in ERβ2-KD-XX and ERβ2$^{-/-}$-XX fish, similar alteration in *DMRT1*, *GSDF*, *SPO11*, and *FOXL2* expressions were also noticed upon androgen/aromatase inhibitor (AI) treatment (de Waal et al., 2009). This indicates that *ERβ2* action is associated with either direct blockage of estrogen action or indirect induction of androgen activity/production (de Waal et al., 2009). To confirm this, we treated the control and ERβ2-KD-XX fish with estrogen and *ERβ* agonist (WAY20070). Although estrogen failed, WAY20070 helped to regain both mitotic and meiotic germ cell proliferation of ERβ2-KD fish to some extent (Figure S2). This insinuates that in a receptor-reduced situation, estrogen fails to form receptor-ligand complex and further fails to regulate the ERE-responsive transcription of downstream genes. The mild rescuing effect associated with WAY20070 might be related to simultaneous triggering of both *ERβ1*- and *ERβ2*-mediated pathways (as discussed above), and/or indirect activation of certain mitosis- and meiosis-related genes, which could further influence germ cell proliferation. Thus, to verify the specificity of *ERβ2* knockdown, we injected synthetic (gene sequence was modified to avoid knockdown) ERβ2-eGFP mRNA into 1- to 2-cell stages of ERβ2-KD-XX F_4 or control embryos. eGFP mRNA was injected into ERβ2-KD-XX embryos, which served as control. Interestingly, we observed nearly 100% rescuing effect in *ERβ2*-overexpressed ERβ2-KD-XX embryos, while no significant influence was noticed in the gonadal development of control groups (Figures 1L and S2). The reoccurrence of gonadal femininity after *ERβ2* overexpression was further confirmed by ISH (Figure S2).

ERβ2 Is Critical for Early Gonadal Development

Earlier, we reported that *ERβ2* expression peaks at 7 days after fertilization (daf) in the germ cells of XX medaka (Chakraborty et al., 2011). This implies that *ERβ2* is likely to play a critical role in germ cell maintenance and gonadal development of medaka. To test the hypothesis, we conducted tetracycline (tet)-responsive conditional knockdown of *ERβ2*, induced at different time points during embryogenesis (knockdown efficiency: 65%–92%, depending on stages), and observed a clear decrease in both mitotic and meiotic proliferation (Figures 2A and 2B) as well as *OLVAS* expression in 0–7 daf, which gradually reduced in later stage groups of tet-ERβ2-KD-XX embryos and became non-significant from control female by 10 daf (Figures 2C–2G). Meanwhile, ethanol-treated tet-ERβ2-KD-XX embryos had similar germ cell numbers as the control ethanol-/doxycycline-treated embryos. These data were further confirmed by both qPCR and whole-mount ISH (WISH) of *ERβ2* at 12 dah and 0 dah, respectively (Figures 2H–2K). Notably, *ERβ2* and aromatase co-localizes in female medaka brain to regulate estrogen synthesis (Okubo et al., 2011; Hiraki et al., 2012), thereby validating the fact that *ERβ2* is critical for embryonic gonadal development. Although slightly different, it was observed that zebrafish *ERα* is essential for embryonic PGC mismigration, and the governance of PGC migration is transferred to *ERβ2* in enhanced estrogen condition (Hu et al., 2014). Additionally, recent investigation using zebrafish CRISPR knockout showed that ERβ2 null mutants have relatively slower pace of female gonadal development and male-biased sex ratio than their control counterparts (Lu et al., 2017). All these data highlight that a delicate ER synergy, which might be species specific in some instances or related to intricate organismal-sexual development, is in place to control gonadal development.

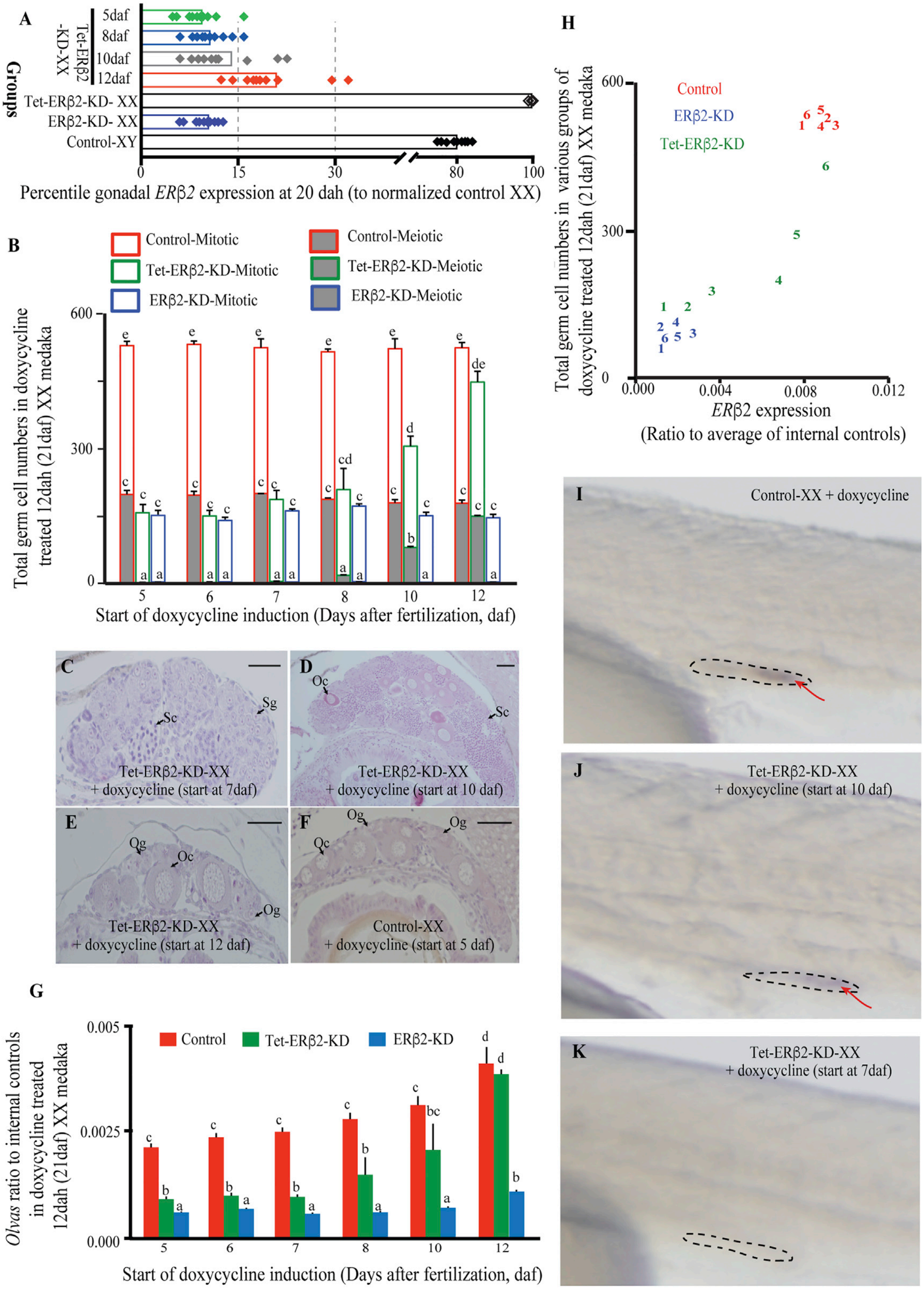

(legend on next page)

Role of *ERβ2* in Early PGC Migration and Maintenance

Our previous data, along with results from tet-ERβ2-KD and ERβ2 expression analysis (Figures 2 and S3) experiments, suggest that the action of *ERβ2* is predominant during 0–7 daf (Chakraborty et al., 2011). This window represents two different major gonadal development-related phenomena, i.e., proliferative mitosis and initiation of meiosis, which in turn determine the phenotypic sex of hatchlings. Total germ cell number in a developing gonad decides the sexual identity (Kurokawa et al., 2007; Tzung et al., 2015). Reports also confirm that the abnormal cell migration is closely related to estrogen and ERs (Gamba et al., 2010; Oviedo et al., 2011). Our present data suggest that *ERβ2* is essential for germ cell population maintenance, and we found severely mismigrated PGCs in the ERβ2-KD embryos (discussed later). To prove the importance of *ERβ2* in PGC migration, firstly, we performed a comprehensive microarray analysis using stage-18 (before the onset of PGC migration) and stage-22 (just after the initiation of PGC migration) control and ERβ2-KD-XX embryos and identified several germ cell migration- and survival-related candidate genes (*SDF1a*, *CXCR4b*, and *WT1b*). Later, we checked the *ERβ2*-responsive and E_2-dependent promoter activity of these candidates by measuring the luciferase activity, using the HEK-293 cell line. We observed an increased *SDF1a* and *CXCR4b* activity, but reduced *WT1b* expression, upon E_2 addition (Figure 3A), highlighting the importance of these genes in estrogenic medaka germ cell maintenance (Kurokawa et al., 2007).

Chromatin immunoprecipitation (ChIP) analysis further confirmed that *ERβ2* directly influences the transcription of *SDF1*, *CXCR4*, and *WT1b* (Figure 3B), and thus illuminated the relevance of *E_2/ERβ2* in germ cell migration. Our qPCR and WISH (Figures 3C and S5) results showed a significant decrease in *SDF1a* and *CXCR4b*, and elevation in *WT1b* expression in the ERβ2-KD group compared with their respective controls. The transcriptional difference among control and ERβ2-KD-XX fish became wider at stage 33 than stage 22 (Figure 3C). Similarly, morpholino knockdown of *CXCR4b* induces PGC mismigration and slowly causes germ cell reduction (Kurokawa et al., 2007), suggesting that a continuous *ERβ2*-dependent regulation is in place to strictly regulate the germ cell settlement and maintenance. Given the fact that *SDF1* and *CXCR4* expresses in somatic and germ cells, respectively, and play significant roles in PGC maintenance in fish (Herpin et al., 2008; Kurokawa et al., 2007), it is probably likely that *ERβ2* simultaneously manipulates both somatic and germ cells and controls PGC maintenance.

Control-XX fish possessed clustered germ cells, while ERβ2-KD-, ERβ2$^{-/-}$-, and AI-treated XX embryos demonstrated disorganized settlement of germ cells in gonadal primordium (Figures 3D–3F and S5). These observations emphasize that the *ERβ2*-deprived situation created by knockdown mimics the estrogen-reduced situation and becomes critical for maintaining embryonic estrogen action and associated sexual development. In this regard, zygotic estrogen synthesis and actions were found to be critical for embryonic gonadal sexuality (Zhou et al., 2016). Contrastingly, CYP19a1-null mutant medaka did not produce primary sex reversal (sex reversal from early stages), or any germ cell reduction. This highlights that, probably, brain type CYP19b isoform, which also expresses in the gonad during early development, is critical for early estrogen synthesis in medaka. Moreover, our finding suggests that CYP19b actions are impaired in ERβ2-KD embryos (Figure S4), further illuminating the importance of estrogen in early gonadal development. Interestingly, the total *OLVAS* expressions remained unaffected at stages 22 and 33 of ERβ2-KD-XX embryos and significant suppression was noticed at 4 daf, at which point the mislocalized cells became autophagic (Figure 3G). This autophagy induction

Figure 2. Determination of *ERβ2*-Responsive Critical Window Period of Gonadal Development in Medaka Using Tet-On Knockdown System

(A) ERβ2-KD tet-on plasmid-injected embryos were treated with doxycycline from either 5, 6, 7, 8, 10, or 12 daf. Tetracycline knockdown effects at representative time points were evaluated by qPCR analysis of *ERβ2* gene at 20 dah and the data were plotted as percentile reduction against control-XX medaka.

(B) At 12 dah, the total germ cell numbers of XX fish were counted and average numbers (n = 18) were plotted against starting days of doxycycline induction to postulate the gonadal sex.

(C–G) Histologically, restricted germ cell proliferation was evident in 7-daf groups (C) but germ cell proliferation in 10-daf (D) and 12-daf (E) groups depicted a control-XX-like pattern (F). qPCR analysis (n = 6) of *OLVAS* gene (G) substantiated the histological observations and suggested XX male development. Representative oogonia (Og), oocyte (Oc), spermatogonia (Sg), and spermatocytes (Sc) are marked with black arrows.

(H–K) At 12 dah, *ERβ2* expression was measured using qPCR (n = 6) and plotted against average total germ cell numbers of respective treatment groups. In (H), the various treatment groups are marked as 1 (5 dah), 2 (6 dah), 3 (7 dah), 4 (8 dah), 5 (10 dah), and 6 (12 dah). WISH analysis using *ERβ2* at 0 dah further corroborated the sex reversal (I–K). WISH gonadal positions and representative *ERβ2* signal are, respectively, marked with black dotted boundary and red arrows.

qPCR analysis was performed using pooled samples, n = 10 individuals/pool. In graphs, data are plotted as means ± SEM; different letters denote significant differences at $p < 0.01$. Scale bars, 50 μm.

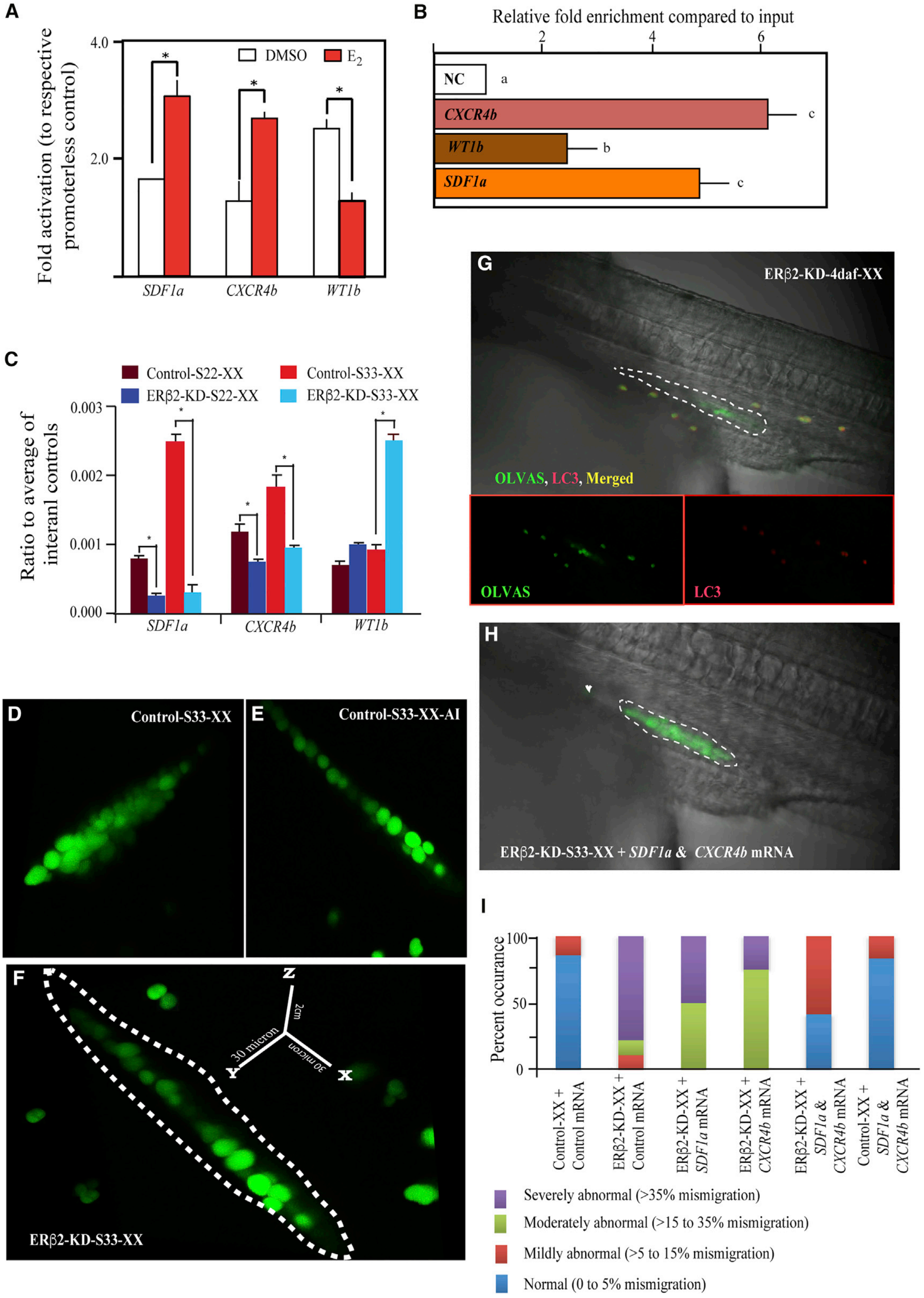

Figure 3. Effect of *ERβ2* on Primordial Germ Cell Fate in Medaka

(A) *In vitro* promoter analysis of several primordial germ cell (PGC) migration and maintenance related genes (using HEK-293 cells) showed estrogen/*ERβ2*-dependent modulations (n = 6). E_2 concentration, 1 ng/mL.

(legend continued on next page)

might be associated with the disturbance in *E_2/ER* associated ion homeostasis (Chakraborty et al., 2017).

To interrogate the *ERβ2* involvement in PGC migration, we transplanted the NANOS-dsRED-ERβ2-KD-positive PGCs to the OLVAS-eGFP host and found that only dsRed-positive cells were prone to mismigration and degradation (Figure S5). Germ cell mismigration is associated with the imbalance of *SDF1* and associated *CXCR4* machinery, and both overexpression and knockdown of *SDF1* or knockdown of *CXCR4* triggers PGC mismigration in fish and frog (Doitsidou et al., 2002; Knaut et al., 2003; Herpin et al., 2008; Takeuchi et al., 2010). Similarly, in the present study, co-injection of SDF1a-Cyan and CXCR4b-mCherry mRNAs in 1-cell-stage control and OLVAS-eGFP-ERβ2-KD cross hybrids helped to resume proper migration (Figures 3H and 3I) and further increased germ cell population in the gonad, which could not be achieved with singular injection of either mRNAs (Figures 3I and S5; Table S1). These results, along with the ChIP data, clearly indicate that *ERβ2* is essential for *SDF1a*- and *CXCR4b*-responsive chemotactic migration (Kurokawa et al., 2007) of PGCs and their settlement in gonadal anlagen. However, the germ cell population in the co-injected fish was half that of the normal gonad at 10 dah and also devoid of proper meiotic initiation, thus implying that PGC migration is independent of PGC maintenance and meiosis. The increased *WT1b* activity in ERβ2-KD embryonic gonads at later stages might be a possible factor in the germ cell survivability (Chakraborty et al., 2016).

ERβ2 Knockdown Disrupts Cellular Calcium Balance and Induces Cell Death

ERs, *SDF1a*, and *CXCR4b* are largely associated with calcium signaling, and the latter two are known to cause cell death through the Ca^{2+}-signaling pathway (Gamba et al., 2010; Teicher and Fricher, 2010). Moreover, our microarray analysis revealed that several Ca^{2+}-signaling-related genes, e.g., *plasma membrane* Ca^{2+} *ATPase* (*PMCA*) *1b*, and *plasma membrane* Na^{+}/Ca^{2+} *exchanger* (*NCX*) *1*, and *Calmodulin* (*CaM*) (Zhang et al., 2012), were significantly altered in the ERβ2-KD-XX fish. To prove our hypothesis that calcium is indispensable for ERβ2-associated cell death, we incubated the OLVAS-eGFP-ERβ2-KD embryo (from 1-cell stage) in $CaCl_2$ (2 mM) solution and checked the PGC migration at stage 33. Unexpectedly, the migration remained identical to that of their ERβ2-KD control counterpart (Figure 4A). Although *NCX1* and *PMCA1b* transcription were significantly reduced in ERβ2-KD-XX, *CaM* showed a pattern completely opposite to that of both control-XX and ERβ2-KD-SDF1/CXCR4b-overexpressed (OV)-XX (at stage 33; Figure 4C). These opposite transcriptional alteration patterns are probably related to their differential role in calcium transport, i.e., Ca^{2+} extrusion (*NCX1* and *PMCA1b*; Brini and Carafoli, 2011) and influx inhibition (*CaM*; Ben-Johny and Yue, 2014; Chi et al., 2017). This, along with calcium homeostasis-related gene transcriptions in ERβ$2^{-/-}$ XX (Figure 4C), implies that *ERβ2* reduction overloads the germ cells with Ca^{2+} by suppressing the transcriptions of Ca^{2+} outflux-related genes and triggers a secondary negative regulatory mechanism, via *CaM* (Griffith et al., 2016), to eventually control the exponential overload. In a subsequent experiment, we reduced the intracellular Ca^{2+} of ERβ2-KD fish by BAPTA_AM, and recorded a substantial decrease in *CaM* level, thus validating the germ cell Ca^{2+} overloading theory. On the contrary, extracellular Ca^{2+} chelation by EGTA did not bring about any significant changes. Further *in vitro* analysis showed that E_2/ERE-responsive *ERβ2* transcription was accelerated by Ca^{2+}, unaffected by intracellular Ca^{2+} chelation, and reduced by extracellular Ca^{2+} chelation (Figure 4B), thereby highlighting the importance of Ca^{2+} influx-outflux ratio in *ERβ2* transcription management (Figure S6). To solve the puzzle of whether addition of excessive Ca^{2+} balances the intracellular and extracellular calcium ion concentration, and further reduces the cell death in mismigrated cells, we sorted three different groups (control-, ERβ2-KD-XX-, and $CaCl_2$-treated ERβ2-KD-XX) of stage-33 OLVAS-eGFP embryonic single-cell suspension, stained

(B and C) ChIP assay demonstrated direct relation between *SDF1a*, *CXCR4b*, *WT1b*, and *ERβ2* (C) (n = 3). Comparative qPCR analysis of stage 22 (migrating PGC) and stage 33 (PGC settled in the gonadal primordium) of ERβ2-KD-XX depicted significant downregulation of *SDF1a* and *CXCR4b* at both stages while *WT1b* showed upregulation at stage 33 (B) (n = 10).
(D–F) The estrogen/ERβ2-responsive mismigration of PGCs to the gonadal primordium was also confirmed by live confocal z-stage imaging of OLVAS-eGFP-control-XX (D), OLVAS-eGFP-XX-AI (E), and OLVAS-eGFP-ERβ2-KD-XX (F) embryos (generated by crossing ERβ2-KD-XX F_3 males with OLVAS-GFP-XX females at stage 33).
(G) Programmed cell death marker (LC3) confirmed the fate of mismigrated cells at 4 daf.
(H and I) Co-injection of *SDF1*a and *CXCR4b* mRNAs (H), but not a singular injection of either *SDF1a* or *CXCR4b* (I), rescued the PGC mismigration. The percentile ratio between total and mismigrating germ cells were subgrouped into normal (0%–5%), mildly abnormal (>5%–15%), moderately abnormal (>15%–35%), and severely abnormal (>35%), and plotted as percent occurrence at group level, to ascertain the rescue effect.
qPCR analyses were performed using pooled samples, n = 10 individuals/pool. In graphs, data are plotted as means ± SEM; different letters and asterisks (*) denote significant differences at $p < 0.05$. The probable gonadal primordia and mismigrating cells are, respectively, marked with white dotted lines and white arrowheads. See also Figure S5 and Table S1.

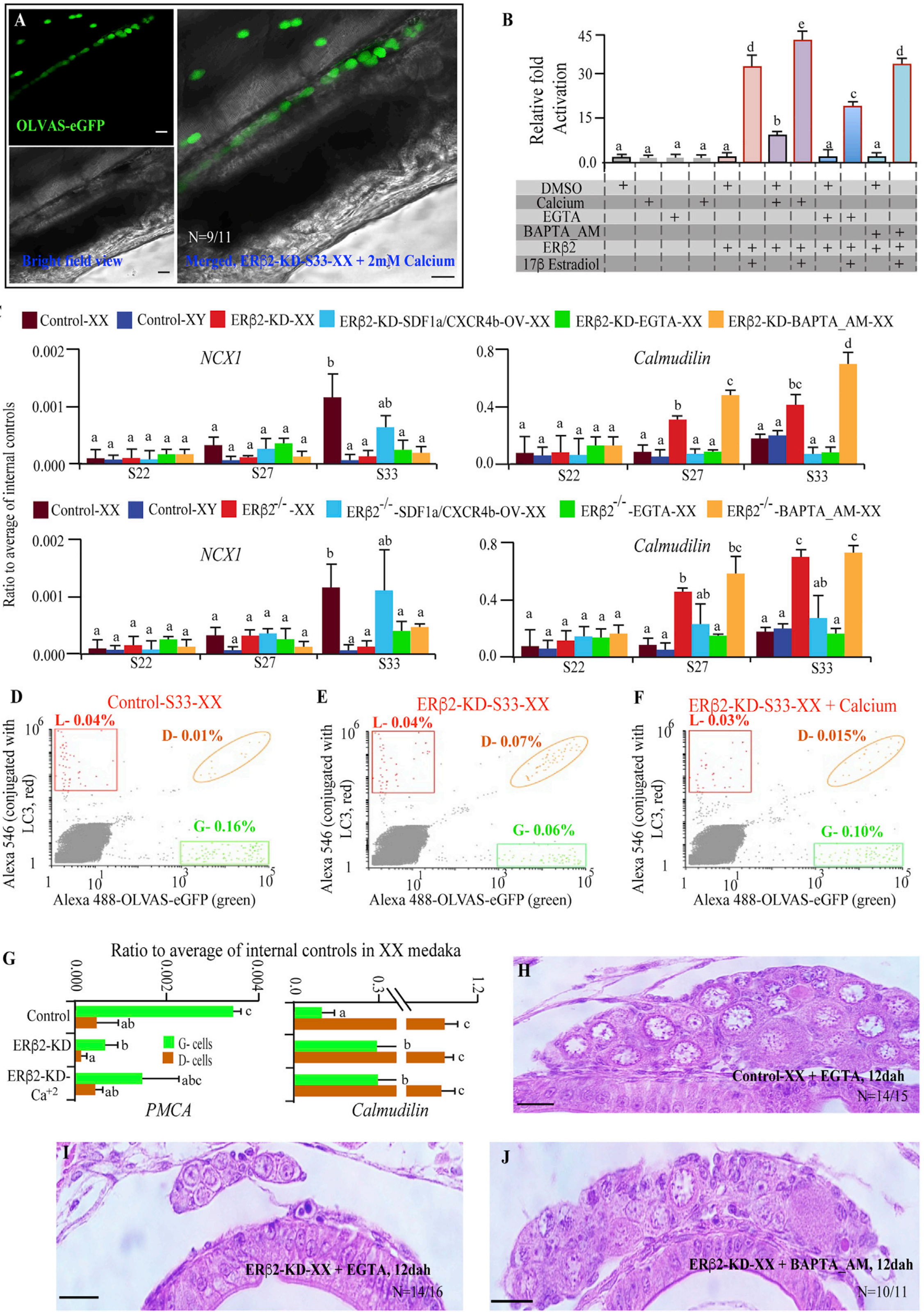

(legend on next page)

with Alexa 488-GFP and Alexa 546-LC3 conjugates, and collected three populations of cells, namely, L (LC3 positive), D (LC3 and eGFP positive), and G (eGFP positive). Morphologically, D- and G-cell populations were similar to PGCs, while L had more likeness with the somatic cells. Moreover, ERβ2-KD-S33-XX fish showed lowest G-cell portion and highest number of D-type cells (Figures 4D–4F). The significantly diminished D population after calcium addition (Figure 4F) further confirmed that cell death was indeed reduced by calcium. qPCR analysis of each cell population depicted a substantially elevated *CaM* level in D population than G-cell fraction, while *PMCA1b* transcription was further suppressed in LC3-positive germ cells (Figure 4G). Our data suggest that cellular-level calcium ion threshold is critical for cell survivability (Borodkina et al., 2016; Chakraborty et al., 2017). Longer incubation with BAPTA_AM, and not with EGTA, increased the ERβ2-KD-XX gonad size and germ cell numbers, further emphasizing that intracellular Ca^{2+} overload is crucial for increased PGC degeneration and further germ cell loss (Figures 4H–4J). As mentioned earlier, ERβ2-KD donor cells, upon being transplanted into control-XX embryos, showed that the majority of donor cells mismigrated and became apoptotic, while the host germ cells did not show such a phenomenon (Figure S5). Most likely, in the ERβ2-reduced situation, when Ca^{2+} imbalance was irreparable, the defective cells underwent programmed cell death. Thus, these findings validate the importance of ERβ2 in germ cell migration and maintenance.

Despite significant effects on PGC maintenance and initiation of primary gonadal sex reversal, when analyzed at adult stages approximately 25%–47% of fish possessed male-like secondary sexual characteristics (fan-like anal fin and forked dorsal fin; Figures 5E–5G), 14%–30% of ERβ2-KD-XX and ERβ2$^{-/-}$-XX fish showed complete testis (Figures 5C and 5H–5I; Table 1), and another 10%–17% had testis-ova (Figure 5D and Table 1). ISH and qPCR analysis of XX testis showed an inverse expression pattern between male- and female-responsive genes, except for *RSPO1* and *β-catenin* (Figure S6), a phenomenon similar to early-stage embryos. However, only 25% (3/12) of adult ERβ2-KD F_0 fish had integration of KD cassette and were able to produce viable progeny. The F_5 and subsequent progenies generated from the three F_0 phenotypic males were used for all our experiments. The XX fertile male production increased in later generations (Table S2) when the ERβ2-KD-XX males were mated with XX normal females, further cementing the significance of *ERβ2* in gonadal sex development and maintenance.

DISCUSSION

In vertebrates, *ER*s and their ligands play several important and conserved physiological functions. Furthermore, maternal estrogen is known to have a critical role in early development and differentiation (Adkins-Regan et al., 1995). Numerous reports suggest that estrogen is synthesized in the mammalian fetus, especially in the brain, and helps in the smooth execution of *ER*-regulated estrogenic functions (Bondesson et al., 2015). Recently, we reported that estrogen is indeed synthesized in the developing medaka embryos (Zhou et al., 2016), interestingly at the same time as zygotic *ER* and brain type aromatase (*CYP19b*) transcriptional onset (Chakraborty et al., 2011; Okubo et al., 2011), thus emphasizing that fetal estrogenic concentration and actions are instrumental in gonadal sex differentiation in medaka (Zhou et al., 2016). In this work, we found that the ER-agonist, ER-antagonist, ER-KD, and ER-null mutation affect the estrogenic actions in medaka from very early stages of embryonic development, thus establishing the fact that E_2/ER actions are critical for embryonic gonadal development.

Figure 4. Effect of *ERβ2* Calcium Homeostasis and Germ Cell Death

(A) Severe PGC mismigration was recorded in $CaCl_2$ (2 mM)-treated ERβ2-KD-XX S33 embryos (n = 11).

(B) *In vitro* ERE-dependent ERβ2 transcriptional analysis (using HEK-293 cells, and various combination of 2 mM $CaCl_2$, 5 pM EGTA, 10 pM BAPTA-AM, 1 ng/mL 17β-estradiol, and 100 ng of ERβ2 overexpression plasmid) demonstrated that cellular calcium concentration significantly alters the E_2-responsive *ERβ2* transcription.

(C) qPCR analysis (n = 10 individuals/group) of several calcium transport-related genes showed significant alteration in both ERβ2-KD (upper panel) and ERβ2$^{-/-}$ (lower panel) fish than their control, SDF1a/CXCR4b-overexpressed, or intra-/extracellular Ca^{2+}-reduced counterparts.

(D–F) Control-XX-treated (D), ERβ2-KD-XX-treated (E), and $CaCl_2$-treated (F) ERβ2-KD-XX embryonic (stage 33) single-cell suspensions were sorted, and three groups of cells (L, D, and G) were collected (n = 4).

(G) The mRNA transcription profile of PMCA1b and CaM were analyzed using qPCR of populations D and G cells to discern the difference between LC3-positive and -negative PGCs.

(H–J) ERβ2-KD-XX or control-XX embryos were incubated in BAPTA_AM (10 pM) and EGTA (5 pM) solution until hatching, and gonadal histology was performed at 12 dah (n = 9) to visualize the germ cell proliferation status.

PGC migration was analyzed using confocal microscopy. In graphs, data are plotted as means ± SEM; different letters denote significant differences at $p < 0.05$. BAPTA_AM and EGTA were used as intracellular and extracellular Ca^{2+} chelator, respectively. Control and OLVAS-eGFP-ERβ2-KD fish lines were used for the experiments. Scale bars, 20 μm.

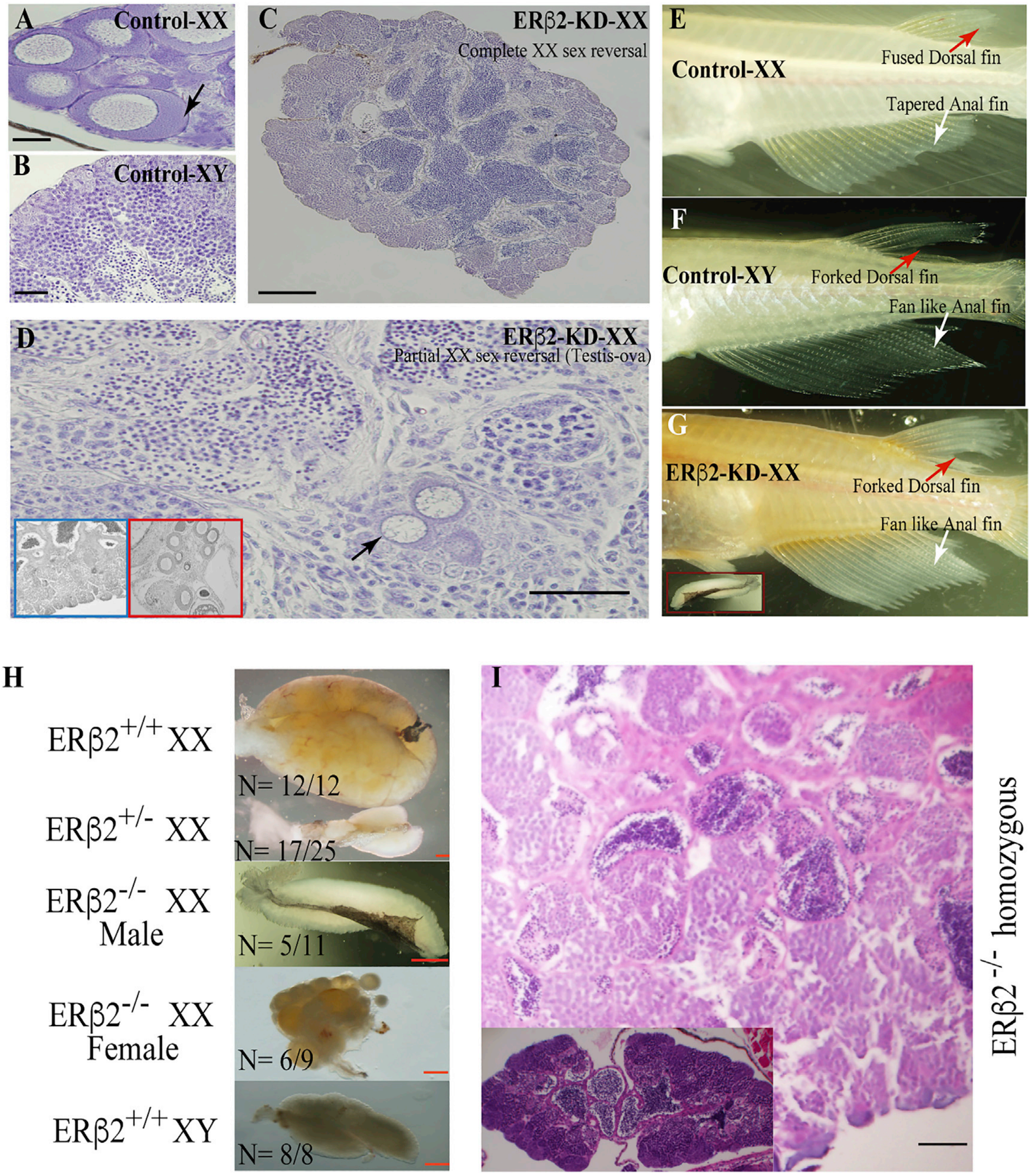

Figure 5. ***ERβ2-KD*** **Affects Gonadal Development in Adulthood**

(A–D) Histological analysis of control-XX (A), control-XY (B), and ERβ2-KD-XX fish showed evidence that knockdown of *ERβ2* results in partial (D) to complete (C) testis formation in XX adults. Inset: representative low-magnification photomicrograph of testicular (blue boundary) and ovarian (red boundary) prevalent gonadal areas.

(E and F) In adulthood, control-XX medaka possesses fused dorsal fin and tapering anal fin (E), while control-XY medaka displays forked dorsal fin and fan-like anal fin (F).

(G) The secondary sexual characters of ERβ2-KD-XX fish resemble those of the control-XY fish. Inset: fully grown adult ERβ2-KD-XX testis.

(H–I) Phenotypic (H) and histological (I) analysis demonstrated that ERβ2$^{-/-}$ null mutation also results in altered gonadal development and functional testis formation in adulthood.

Black, red, and white arrows indicate oocytes, dorsal fin, and anal fin, respectively. Scale bars, 50 μm. See also Figure S6 and Table S2.

Table 1. Summary of Gonadal Sex Profile in Various Experimental Fish at Adulthood

Groups[a]	Full-Grown Ovary (%)	Abnormal Ovary[b] (%)	Testis (%)	Testis-Ova (%)	Breeding Behavior[c] (%)
Control-XX	65/66 (98.5)	1/66 (1.5)	0/66 (0)	0/66 (0)	A: 0/10 (0)<br>B: 10/10 (100)<br>C: 0/10 (0)
ERβ2-KD (F_{10} generation)	18/115 (15.7)	50/115 (43.5)	35/115 (30.4)	12/115 (10.4)	A: 8/12 (75)<br>B: 0/12 (0)<br>C: 4/12 (25)
ERβ$2^{+/+}$ XX (F_{11} generation)	28/28 (100)	0/28 (0)	0/0 (0)	0/28 (0)	A: 0/8 (0)<br>B: 8/8 (100)<br>C: 0/8 (0)
ERβ$2^{+/-}$ XX (F_{11} generation)	26/51 (51.0)	21/51 (41.1)	0/51 (0)	4/51 (7.9)	A: 2/15 (13.3)<br>B: 10/15 (66.7)<br>C: 3/15 (20)
ERβ$2^{-/-}$ XX (F_{11} generation)	11/35 (31.4)	13/35 (37.1)	5/35 (14.3)	6/35 (17.1)	A: 5/14 (35.7)<br>B: 4/14 (28.6)<br>C: 5/14 (35.7)

[a]Four different populations of fish, generated from four different parents, were mixed and sampled randomly for histological analysis to ascertain the gonadal sexual status and sex reversal. All the examined fish were screened with DMY-specific genomic PCR and, whenever necessary, followed by ERβ2-mutation-specific PCR and sequencing.

[b]Vitellogenesis was not initiated, relatively hypotrophic (less) germ cell proliferation, etc.

[c]Randomly selected fish (from the aforementioned pool of screened fish) were examined for secondary sexual characteristics and tested with mature normal fish of opposite sex. A, B, and C, respectively denote male-like, female-like, and no sexual behavior.

Conversely, Nakamoto et al. (2018) found that null mutation of CYP19a1 gene does not affect the early gonadal development. In medaka, despite the rapid reduction in maternal estrogen from yolk stores, it is probable that traces of remaining estrogen or other estrogen by-products might be enough to drive the normal gonadal development in CYP19a$1^{-/-}$ medaka. Moreover, chronologically, first zygotic expression of *CYP19b* (2 daf) and *ERβ2* (2 daf) precedes the *CYP19a1* expression (5–10 dah), and ERβ2 knockdown reduces the *CYP19b* expression in both gonad and brain. It is possible that during early development estrogen synthesis is mainly regulated by *CYP19b* or some other estrogen-like machinery (Nakamoto et al., 2018). Notably, it has been repeatedly shown that environmental estrogenic chemicals can mimic endogenous estrogen's action and alter sexual development in various organisms, suggesting that estrogen actions are vital for sexual differentiation (Mizoguchi and Valenzuela, 2016). Further investigations to decipher the sources of endogenous estrogenic components are necessary to completely understand the estrogenic role in early gonadal development.

In this work, we found that co-dependent actions of newly synthesized estrogen and *ERβ2* is vital for PGC chemotactic migration, gonadal settlement, maintenance, and proliferation. The abundance of *ERβ2* in both somatic and germ cells further accentuates the germ-soma interactions in sex management. Our data suggest that, during early embryonic development, *ERβ2* produced in the somatic cells directly regulates *SDF1a* transcription and simultaneously modulates *CXCR4b* actions in germ cells to control the directed migration of PGCs (Herpin et al., 2008; Kurokawa et al., 2007). In an ERβ2-reduced situation, the production of both *SDF1a* and *CXCR4* were directly hampered and affected the mismigration, as evidenced by rescuing of PGC mismigration by co-overexpression of both *SDF1a* and *CXCR4*. However, studies show that SDF1 knockdown or overexpression can induce PGC mismigration, thus emphasizing that SDF1 concentrations and associated CXCR4 receptor saturation are critical for proper PGC migration in vertebrates (Herpin et al., 2008; Takeuchi et al., 2010). Although the critical concentration of *SDF1a* in medaka PGC migration still needs confirmation, in the present investigation we observed that *SDF1a* overexpression might have restored the *SDF1a* balance in somatic cells to resume proper migration. In this regard, Herpin et al. (2008) found that 30% or more *SDF1a* knockdown can affect PGC migration in medaka. It is possible that ERβ2 assisted the reduction of both SDF1a and that CXCR4 affected other receptors (e.g., CXCR7) and further aggravated the situation (Boldajipour et al., 2008). The reduction in *ERβ2* also might have affected the E_2-induced-*ER*-responsive AKT phosphorylation (Stabile et al., 2006) and further reduced the PGC migration into gonadal anlagen (Moe-Behrens et al., 2003).

In an exemplary study, using morpholino knockdown and cell transplantation, Tzung et al. (2015) demonstrated

that the threshold number of PGC is required to retain ovarian stability in zebrafish and is instrumental for testicular differentiation. In our ERβ2-KD fish, PGC mismigration and cell death likely significantly reduce the initial PGC number in the embryonic gonad and mimic a situation similar to male-type gonad in medaka. This gonadal state is further aggravated by the reduced estrogen signaling-associated transcription of male-biased genes. However, in this regard the indirect involvement of the interactively modulated AR pathway (Wu et al., 2017) cannot be overlooked. Since *ERβ2* actions are critical throughout embryogenesis, it is possible that elimination of *ERβ2* affects several other important pathways, and in turn orchestrates the sex reversal. Despite a significant increase in male-biased gene transcription and germ cell reduction, both initiated by *ERβ2* reduction, the percentages of adult sex reversal were somewhat less than expected. This suggests that some unknown mechanism, probably variable non-genomic action or substitution effect or a threshold of gene(s)/pathway(s), are in action, which needs further investigation. In contrast to previous reports on the absence of sex-reversal phenotype in ER-KO mice (Bondesson et al., 2015; Dupont et al., 2000; Hamilton et al., 2014), it was recently found that biallelic/monoallelic mutation of *ERβ* causes sex reversal in humans (Baetens et al., 2018). This difference has been suggestively attributed to the non-genomic ER action, i.e., MAPK signaling (Baetens et al., 2018). Moreover, the intensity of sex reversal in humans is significantly related to zygosity, further supporting the idea that a threshold limit might be important for functional manifestation of *ERβ2* actions in medaka. Nevertheless, irrespective of different non-genomic interaction or fish-specific genome duplication, it seems that the estrogen/ER pathway directly controls the reproductive fitness in both human and medaka. Hence, this study will help us to rethink the estrogen/*ER* involvement in successful reproduction and reproductive disorder management.

In conclusion, our data suggest that *ERβ2* has multi-point regulation, starting from regulation of chemotactic germ cell migration, calcium homeostasis, and cell sustenance, to controlling meiotic initiation, which eventually influences sexual development. Our data also highlight the importance of *ERβ2* in estrogen transmission to gonad to maintain the gonadal sexuality. Thus, this study might be a key to comprehending the diverse estrogen-reproduction relationship in vertebrates.

EXPERIMENTAL PROCEDURES

Ethics Statement

All treatments of animals in this study followed the guidelines of the National Institute for Basic Biology and were approved by the Institutional Animal Care and Use Committee of National Institutes of Natural Sciences and Ehime University Animal Use and Ethics Committee. All surgery was performed under Tricaine-S anesthesia, and all efforts were made to minimize suffering.

Experiments

The plasmids were constructed using various commercially available vectors as required. ERβ2 knockdown was carried out using a previously published protocol (Chakraborty et al., 2016). *In vitro* and *in vivo* analysis was carried out using HEK-293 cells and several medaka strains, respectively. Histological, qPCR (primer details in Table S3), ChIP, germ cell transplantation, flow cytometry, and cell-sorting analysis were performed using pre-adjusted protocols. All the *in vivo* samples were first examined for genetic sex and then pooled (sexwise and/or groupwise) or individually used for RNA isolation and cDNA synthesis, and further subjected to subsequent analysis. A detailed description of procedures used in this study is provided in Supplemental Information.

SUPPLEMENTAL INFORMATION

Supplemental Information can be found online at https://doi.org/10.1016/j.stemcr.2019.07.013.

AUTHOR CONTRIBUTIONS

T.C., S.M., and L.Y.Z. contributed equally. T.C., T.I., and Y.N. conceived the idea. T.C., S.M., and L.Y.Z. performed the experiments and statistical analysis. All authors (except L.Y.Z.) provided chemicals and materials for experiments. T.C., S.M., K.O., and Y.N. prepared the manuscript.

ACKNOWLEDGMENTS

The authors are thankful to Shinichi Miyagawa (Associate Professor, Tokyo University of Science, Japan) and Yukiko Ogino (Associate Professor, Kyushu University, Japan) for their help with the research. The authors extend their sincere gratitude to National Bio Resource Project Medaka of Ministry of Education, Culture, Sports, Science and Technology, Japan for their help with various transgenic and inbred medaka. This work was in part supported by grants from Science and Technology Agency (SORST Program) and the Ministry of Education, Culture, Sports, Science and Technology, Japanese Society for the Promotion of Science Kakenhi, grant nos. 23688022, 16H04981, 23380110, 18K14520, and 19H03049; Sumitomo grant no. 180959; and Ehime University post-doctoral research grant, Japan.

Received: August 25, 2018
Revised: July 17, 2019
Accepted: July 18, 2019
Published: August 13, 2019

REFERENCES

Adkins-Regan, M., Ottinger, M.A., and Park, J. (1995). Maternal transfer of estradiol to egg yolks alters sexual differentiation of avian offspring. J. Exp. Zool. *271*, 466–470.

Antal, M.C., Petit-Demouliere, B., Meziane, H., Chambon, P., and Krust, A. (2012). Estrogen dependent activation function of ERβ

is essential for the sexual behavior of mouse females. Proc. Natl. Acad. Sci. U S A *109*, 19822–19827.

Baetens, D., Guran, T., Mendonca, B.B., Gomes, N.L., Couwer, L.D., Peelman, F., Verdin, H., Vuylsteke, M., Linden, M.V., Stoop, H., et al. (2018). Biallelic and monoallelic ESR2 variants associated with 46, XY disorders of sex development. Genet. Med. *20*, 717–727.

Ben-Johny, M., and Yue, D.T. (2014). Calmodulin regulation (calmodulation) of voltage-gated calcium channels. J. Gen. Physiol. *143*, 679–692.

Boldajipour, B., Mahabaleshwar, H., Kardash, E., Reichman-Fried, M., Blaser, H., Minima, S., Wilson, D., Xu, Q., and Raz, E. (2008). Control of chemokine-guided cell migration by ligand sequestration. Cell *132*, 463–473.

Bondesson, M., Hao, R., Lin, C.Y., Williams, C., and Gustafsson, J.A. (2015). Estrogen receptor signaling during vertebrate development. Biochim. Biophys. Acta *1849*, 142–151.

Borodkina, A.V., Shatrova, A.N., Deryabin, P.I., Griukova, A.A., Abushik, P.A., Antonov, S.M., Nikolay, N.N., and Burova, E.B. (2016). Calcium alterations signal either to senescence or to autophagy induction in stem cells upon oxidative stress. Aging (Albany NY) *8*, 3400–3416.

Brini, M., and Carafoli, E. (2011). The plasma membrane Ca^{2+} ATPase and the plasma membrane sodium calcium exchanger cooperate in the regulation of cell calcium. Cold Spring Harb. Perspect. Biol. *3*, a004168.

Chakraborty, T., Shibata, Y., Zhou, L.Y., Katsu, Y., Iguchi, T., and Nagahama, Y. (2011). Differential expression of three estrogen receptor subtype mRNAs in gonads and liver from embryos to adults of the medaka, *Oryzias latipes*. Mol. Cell. Endocrinol. *333*, 47–54.

Chakraborty, T., Mohapatra, S., Tobayama, M., Ohta, K., Ryu, Y.W., Kazeto, Y., Ohta, K., Zhou, L.Y., Nagahama, Y., and Matsubara, T. (2017). Hatching enzymes disrupt aberrant gonadal degeneration by the autophagy/apoptosis cell fate decision. Sci. Rep. *7*, 3183.

Chakraborty, T., Zhou, L.Y., Chaudhari, A., Iguchi, T., and Nagahama, Y. (2016). *Dmy* initiates masculinity by altering *Gsdf/Sox9a2/Rspo1*expression in medaka (*Oryzias latipes*). Sci. Rep. *6*, 19480.

Chen, Y., Breen, K., and Pepling, M.E. (2009). Estrogen can signal through multiple pathways to regulate oocyte cyst breakdown and primordial follicle assembly in the neonatal mouse ovary. J. Endocrinol. *202*, 402–417.

Chi, C.-H., Tang, C.-Y., and Pan, C.-Y. (2017). Calmodulin modulates the Ca^{2+}-dependent inactivation and expression level of bovine $Ca_V2.2$ expressed in HEK293T cells. IBRO Rep. *2*, 63–71.

de Waal, P.P., Leal, M.C., Garcia-Lopez, A., Liarte, S., Jonge, H., Hinfray, N., Brion, F., Schulz, R.W., and Bogerd, J. (2009). Oestrogen-induced androgen insufficiency results in a reduction of proliferation and differentiation of spermatogonia in the zebrafish testis. J. Endocrinol. *202*, 287–297.

DeFalco, T., and Capel, B. (2009). Gonad morphogenesis in vertebrates: divergent means to a convergent end. Annu. Rev. Cell Dev. Biol. *25*, 457–482.

Doitsidou, M., Reichman-Fired, M., Stebler, J., Koprunner, M., Dorries, J., Meyer, D., Esguerra, C.V., Leung, T., and Raz, E. (2002). Guidance of primordial germ cell migration by the chemokine SDF-1. Cell *111*, 647–659.

Dupont, S., Krust, A., Gansmuller, A., Dierich, A., Chambon, P., and Mark, M. (2000). Effect of single and compound knockouts of estrogen receptors alpha (ERalpha) and beta (ERbeta) on mouse reproductive phenotypes. Development *127*, 4277–4291.

Gamba, L., Cubedo, N., Ghysen, A., Lutfalla, G., and Dambly-Chaudiere, C. (2010). Estrogen receptor ESR1 controls cell migration by repressing chemokine receptor CXCR4 in the zebrafish posterior lateral line system. Proc. Natl. Acad. Sci. U S A *107*, 6358–6363.

Gautier, A., Le Gac, F., and Lareyre, J.J. (2011). The *gsdf* gene locus harbors evolutionary conserved and clustered genes preferentially expressed in fish previtellogenic oocytes. Gene *472*, 7–17.

Griffith, T., Tsaneva-Atanasova, K., and Mellor, J.R. (2016). Control of Ca^{2+} influx and calmodulin activation by SK channels in dendritic spines. PLoS Comput. Biol. *12*, e1004949.

Hamilton, K.J., Arao, Y., and Korach, K.S. (2014). Estrogen hormone physiology: reproductive findings from estrogen receptor mutant mice. Reprod. Biol. *14*, 3–8.

Herpin, A., Fischer, P., Liedtke, D., Kluever, N., Neuner, C., Raz, E., and Schartl, M. (2008). Sequential SDF1a and b-induced mobility guides medaka PGC migration. Dev. Biol. *320*, 319–327.

Hiraki, T., Takeuchi, A., and Tsumaki, T. (2012). Female-specific target sites for both oestrogen and androgen in the teleost brain. Proc. Biol. Sci. *279*, 5014–5023.

Hu, J., Sun, S., Guo, M., and Song, H. (2014). Use of antagonists and morpholinos in loss-of-function analyses: estrogen receptor ESR2a mediates the effects of 17alpha-ethinylestradiol on primordial germ cell distribution in zebrafish. Reprod. Biol. Endocrinol. *12*, 40.

Khattri, A., Pandey, R.K., Gupta, N.J., Chakravarty, B., Deenadayal, M., Singh, L., and Thangaraj, K. (2009). Estrogen receptor beta gene mutations in Indian infertile men. Mol. Hum. Reprod. *15*, 513–520.

Knaut, H., Werz, C., Geisler, R., and Nusslein-Volhard, C. (2003). A zebrafish homologue of the chemokine receptor Cxcr4 is a germ-cell guidance receptor. Nature *421*, 279–282.

Kurokawa, H., Saito, D., Nakamura, S., Katoh-Fukui, Y., Ohta, K., Baba, T., Morohashi, K., and Tanaka, M. (2007). Germ cells are essential for sexual dimorphism in the medaka gonad. Proc. Natl. Acad. Sci. U S A *104*, 16958–16963.

Lu, H., Cui, Y., Jiang, L., and Ge, W. (2017). Functional analysis of nuclear estrogen receptors in zebrafish reproduction by genome editing approach. Endocrinology *158*, 2292–2308.

Matsuda, M., Nagahama, Y., Shinomiya, A., Sato, T., Matsuda, C., Kobayashi, T., Morrey, C.E., Shibata, N., Asakawa, S., Shimizu, N., et al. (2002). DMY is a Y-specific DM-domain gene required for male development in the medaka fish. Nature *417*, 559–563.

Mizoguchi, B.A., and Valenzuela, N. (2016). Ecotoxicological perspectives of sex determination. Sex. Dev. *10*, 45–57.

Moe-Behrens, G.H., Klinger, F.G., Eskild, W., Grotmol, T., Haugen, T.B., and De Felici, M. (2003). Akt/PTEN signaling mediates estrogen-dependent proliferation of primordial germ cells *in vitro*. Mol. Endocrinol. *17*, 2630–2638.

Nakamoto, M., Shibata, Y., Ohno, K., Usami, T., Kamei, Y., Taniguchi, Y., Todo, T., Sakamoto, T., Young, G., Swanson, P., et al. (2018). Ovarian aromatase loss-of-function mutant medaka undergo ovary degeneration and partial female-to male reversal after puberty. Mol. Cell. Endocrinol. *460*, 104–122.

Okubo, K., Takeuchi, A., Chaube, R., Paul-Prasanth, B., Kanda, S., Oka, Y., and Nagahama, Y. (2011). Sex differences in aromatase gene expression in the medaka brain. J. Neuroendocrinol. *23*, 412–423.

Oviedo, P.J., Sobrino, A., Laguna-Fernandez, A., Novella, S., Tarin, J.J., Garcia-Perez, M.A., Sanchis, J., Cano, A., and Hermenegildo, C. (2011). Estradiol induces endothelial cell migration and proliferation through estrogen receptor-enhanced RhoA/ROCK pathway. Mol. Cell. Endocrinol. *335*, 96–103.

Shibata, Y., Paul-Prasanth, B., Suzuki, A., Usami, T., Nakamoto, M., Matsuda, M., and Nagahama, Y. (2010). Expression of gonadal soma derived factor (GSDF) is spatially and temporally correlated with early testicular differentiation in medaka. Gene Expr. Patterns *10*, 283–289.

Stabile, V., Russo, M., and Chieffi, P. (2006). 17β-Estradiol induces Akt-1 through estrogen receptor-β in the frog (*Rana esculenta*) male germ cells. Reproduction *132*, 477–484.

Takeuchi, T., Tanigawa, Y., Minamide, R., Ikenishi, K., and Komiya, T. (2010). Analysis of SDF-1/CXCR4 signaling in primordial germ cell migration and survival or differentiation in Xenopus laevis. Mech. Dev. *127*, 146–158.

Teicher, B.A., and Fricher, S.P. (2010). CXCL12 (SDF-1)/CXCR4 pathway in cancer. Clin. Cancer Res. *16*, 2927–2931.

Tzung, K.-W., Goto, R., Saju, J.M., Sreenivasan, R., Saito, T., Arai, K., Yamaha, E., Hossain, M.S., Calvert, M.E., and Orban, L. (2015). Early depletion of primordial germ cells in zebrafish promotes testis formation. Stem Cell Reports *4*, 61–73.

Windsor, F.M., Ormerod, S.J., and Tyler, C. (2018). Endocrine disruption in aquatic systems: up-scaling research to address ecological consequences. Biol. Rev. *93*, 626–641.

Wu, Q., Shao, H., Darwin, E.D., Li, J., Li, J., Yang, B., Webster, K.A., and Yu, H. (2009). Extracellular calcium increases CXCR4 expression on bone marrow-derived cells and enhances pro-angiogenesis therapy. J. Cell. Mol. Med. *13*, 3764–3773.

Wu, W.F., Maneix, L., Insunza, J., Nalvarte, I., Antonson, P., Kere, J., Yu, N.Y., Tohonen, V., Katayama, S., Einarsdottir, E., et al. (2017). Estrogen receptor B, a regulator of androgen receptor signaling in the mouse ventral prostate. Proc. Natl. Acad. Sci. U S A *114*, E3816–E3822.

Zhang, Y., Li, Z., Sacks, D.B., and Ames, J.B. (2012). Structural basis for Ca^{2+}- induced activation and dimerization of estrogen receptor α by calmodulin. J. Biol. Chem. *287*, 9336–9344.

Zhou, L.Y., Chakraborty, T., Zhou, Q., Mohapatra, S., Nagahama, Y., and Zhang, Y.G. (2016). Rspo1-activated signaling molecules are sufficient to induce ovarian differentiation in XY medaka (*Oryzias latipes*). Sci. Rep. *6*, 19543.

Stem Cell Reports, Volume *13*

Supplemental Information

Estrogen Receptor β2 Oversees Germ Cell Maintenance and Gonadal Sex Differentiation in Medaka, *Oryzias latipes*

Tapas Chakraborty, Sipra Mohapatra, Lin Yan Zhou, Kohei Ohta, Takahiro Matsubara, Taisen Iguchi, and Yoshitaka Nagahama

Supplemental Figures

Figure S1

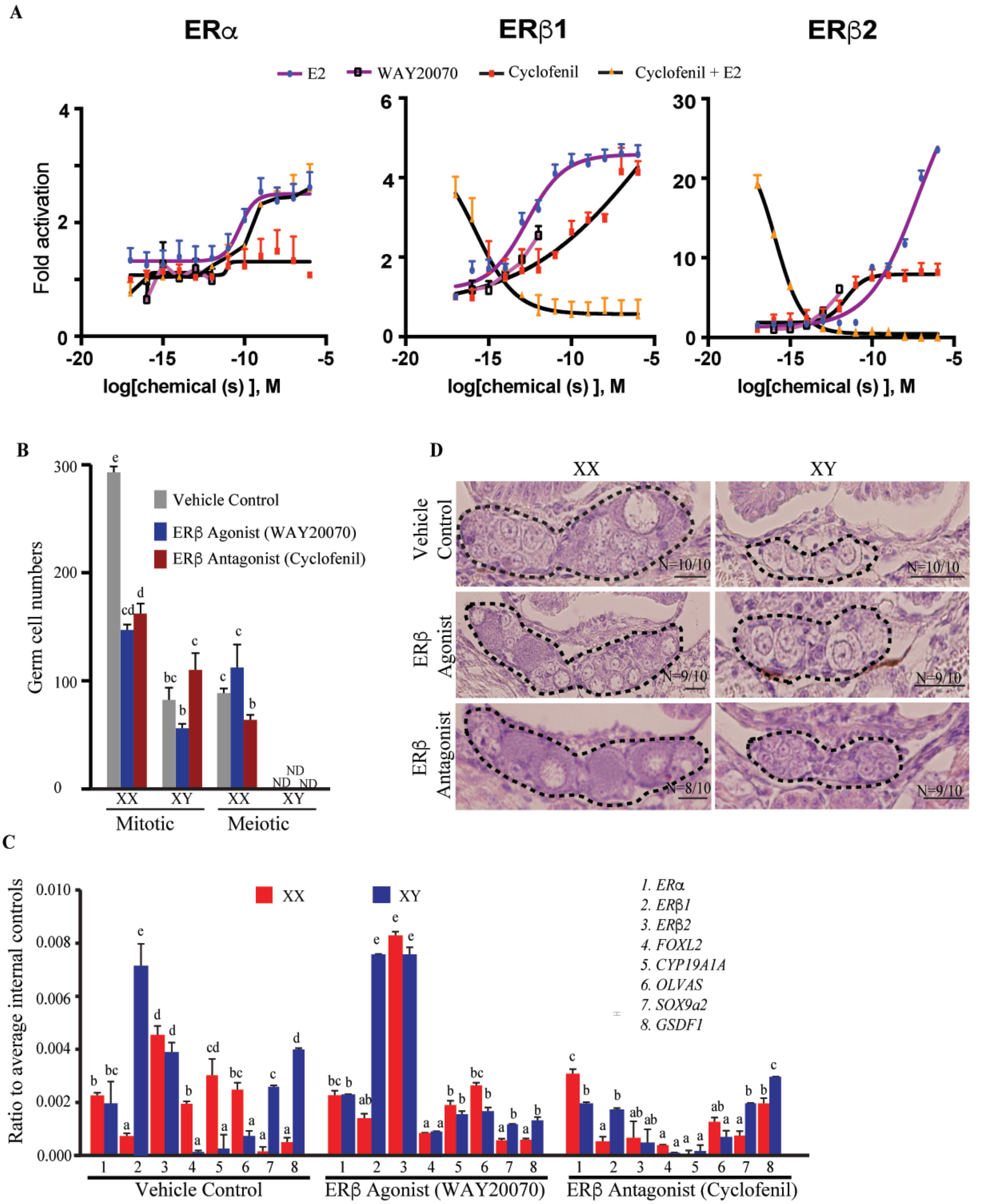

Figure S1. Effect of ERβ agonist and antagonist on medaka estrogen receptor regulation and gonad development. Related to Figure 1. A. Estrogen, ERβ agonist and antagonist regulated *in vitro* transcriptional activity of various medaka estrogen receptors. Transcriptional activity profiles of *ERα*, *ERβ1* and *ERβ2* generated using 17β-estradiol (E_2), WAY20070 (ERβ agonist), Cyclofenil (ERβ antagonist) and Cyclofenil with E_2 depicts differential activation pattern. The experiments were repeated for 6 times and the cumulative data were analysed using Graph Pad Prism software. **B.** ERβ agonist and antagonist treatment influenced the mitotic and meiotic germ cell counts in 10 dah medaka. **C.** Comparative transcriptional profiling of various sexually biased genes was performed to corroborate the changes in germ cell count. **D.** Gonadal histology of different groups depicted no remarkable difference between the experimental groups.

Note: Triplicates, each containing 10 embryos were used for QPCR analysis. 10 XX and 10 XY embryos from each treatment group were separately used for gonadal histology and germ cell counting. In graphs, data are plotted as means ± SEM; different letters denote significant differences at $p<0.01$. Scale bars, 50μm.

Figure S2

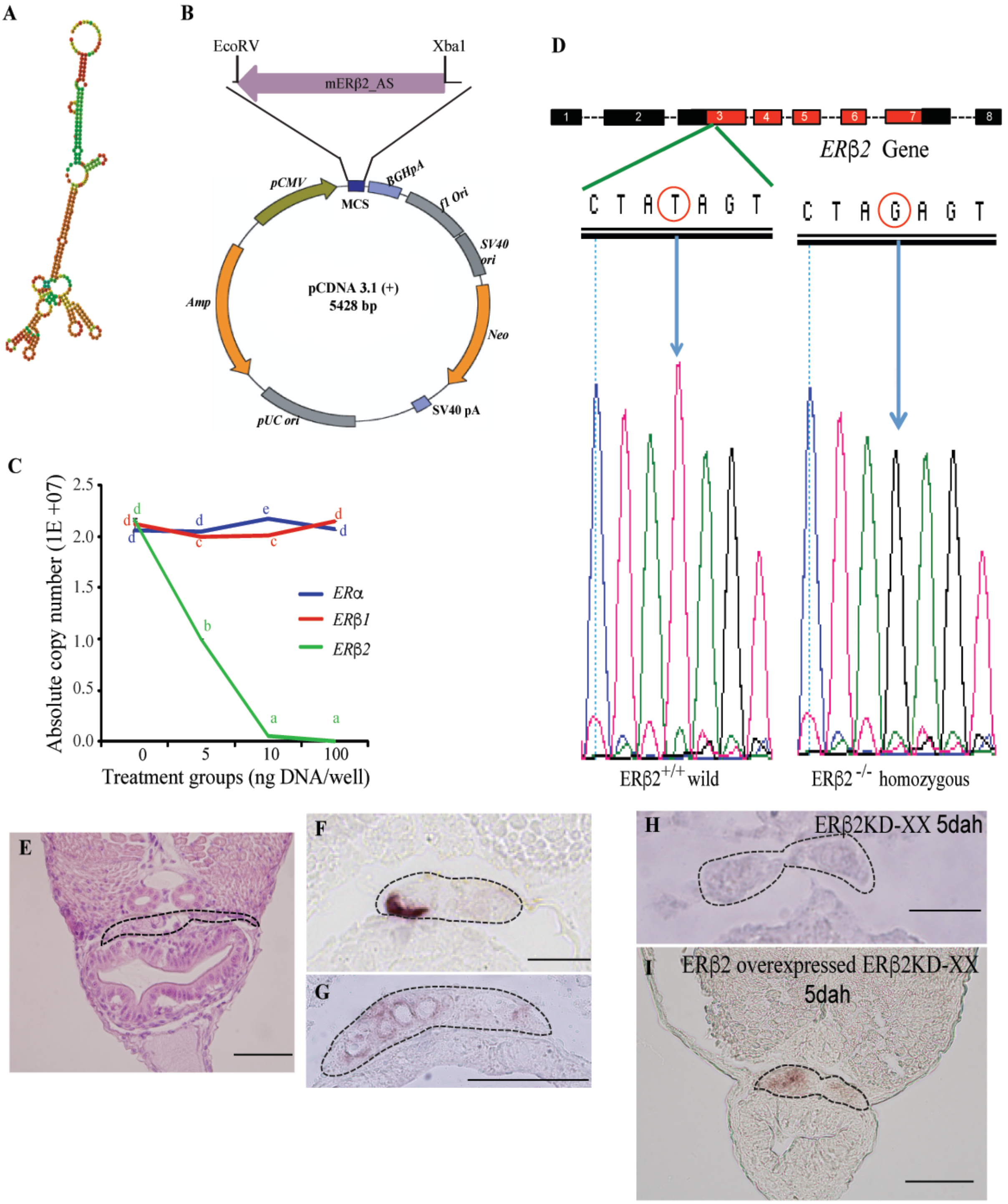

Figure S2. Construction and validation of ERβ2 knockdown/ Knockout. **A-B. Related to Figure 1.** The antisense fragment/probe, predicted to have best secondary AS-RNA structure (A), was inserted in a reverse orientation into the pcDNA 3.1 vector to generate the knockdown plasmid (B). **C.** The efficacy of knockdown construct was determined *in vitro* by co-transfection of *ERα/ERβ1/ERβ2* overexpression plasmids and different concentration of ERβ2-AS plasmid. **D.** Schematic diagram (upper panel) represents the ERβ2 exon (filled box), intron (dotted lines), untranslated regions (black box) and open reading frame (red box) assembly at chromosome 22:23,116,691-23,156,480. The representative sequence chromatograms (lower panel) showing the single nucleotide difference (A > G, marked with red circle and blue arrow) between wild and homozygous ERβ2-KO medaka. **E-G.** Histologically, at 10dah, the $ER\beta2^{-/-}$ XX gonad (marked with dotted black boundary) showed fewer occurrences of germ cells and male type gonadal development (E). *In situ* hybridization **(***ISH***)** analysis using *GSDF* (F) and *ERβ2* (G) also indicated gonadal masculinity **H-I.** *ISH* analysis of ERβ2-

KD-XX (H) and *ERβ2* overexpressed ERβ2-KD-XX (I) fish using *SPO11* (meiotic marker) further confirmed the rescuing effect of gonadal feminity. Scale bars, 100µm.

Figure S3

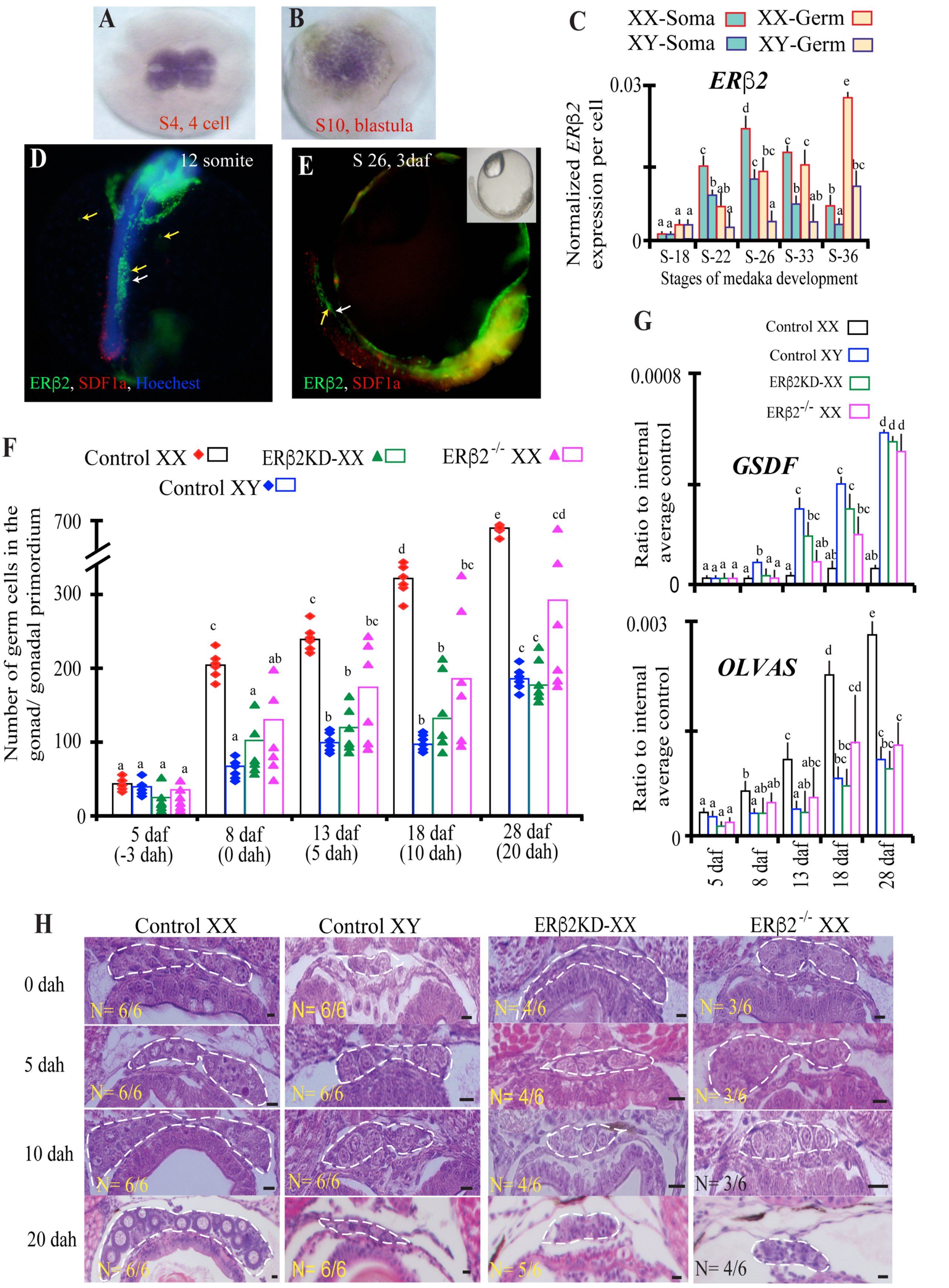

Figure S3: Effect of ERβ2 on early gonadal development and primary sex reversal in medaka. A-B. Related to Figure 1. Wholemount *ISH* (*WISH*) showing the cellular localization of *ERβ2* mRNA in 4 cell (A) and blastula (B) stage

medaka embryo. **C**. *ERβ2* mRNA profiles were analysed using germ and somatic cells sorted from DMY genotyped individual NANOS-dsRED embryos (N= 6 (XY), 8 (XX)), and plotted to determine sex biased *ERβ2* expression profile. **D-E.** Two color fluorescent *WISH* (*FWISH*) using *ERβ2* (green) and *SDF1a* (red) demonstrating their distribution in developing medaka embryos at 12 somite (D) and 3daf (E). The yellow and white arrows respectively denote the representative germ cells and putative gonadal analgen. **F-H.** Assessment of gonadal sexuality and primary sex reversal in ERβ2-KD-XX and ERβ2$^{-/-}$-XX fish. Total germ cell numbers (F) were counted using HE stained serially sectioned samples at 5, 8, 13, 18, and 28daf and individual fish data (marked with triangle, etc) were plotted to ascertain the reversal of gonadal sexuality in both ERβ2-KD and ERβ2$^{-/-}$ XX fish. Simultaneously, the *OLVAS* and *GSDF* mRNA profiles (G) were analysed (N=6 individual) using QPCR to validate the initiation of sex reversal in various experimental groups. Candidate HE stained photomicrographs (H) also demonstrates the varying degree of sex reversal (ERβ2-KD-XX (66.7-83.3%) > ERβ2$^{-/-}$-XX (50-66.7%)) initiated in ERβ2-reduced juvenile gonads before 28daf. Note: In graphs, different letters in small case (a, b, etc.) indicate significant differences at $p<0.05$; Scale bars, 10μm.

Figure S4

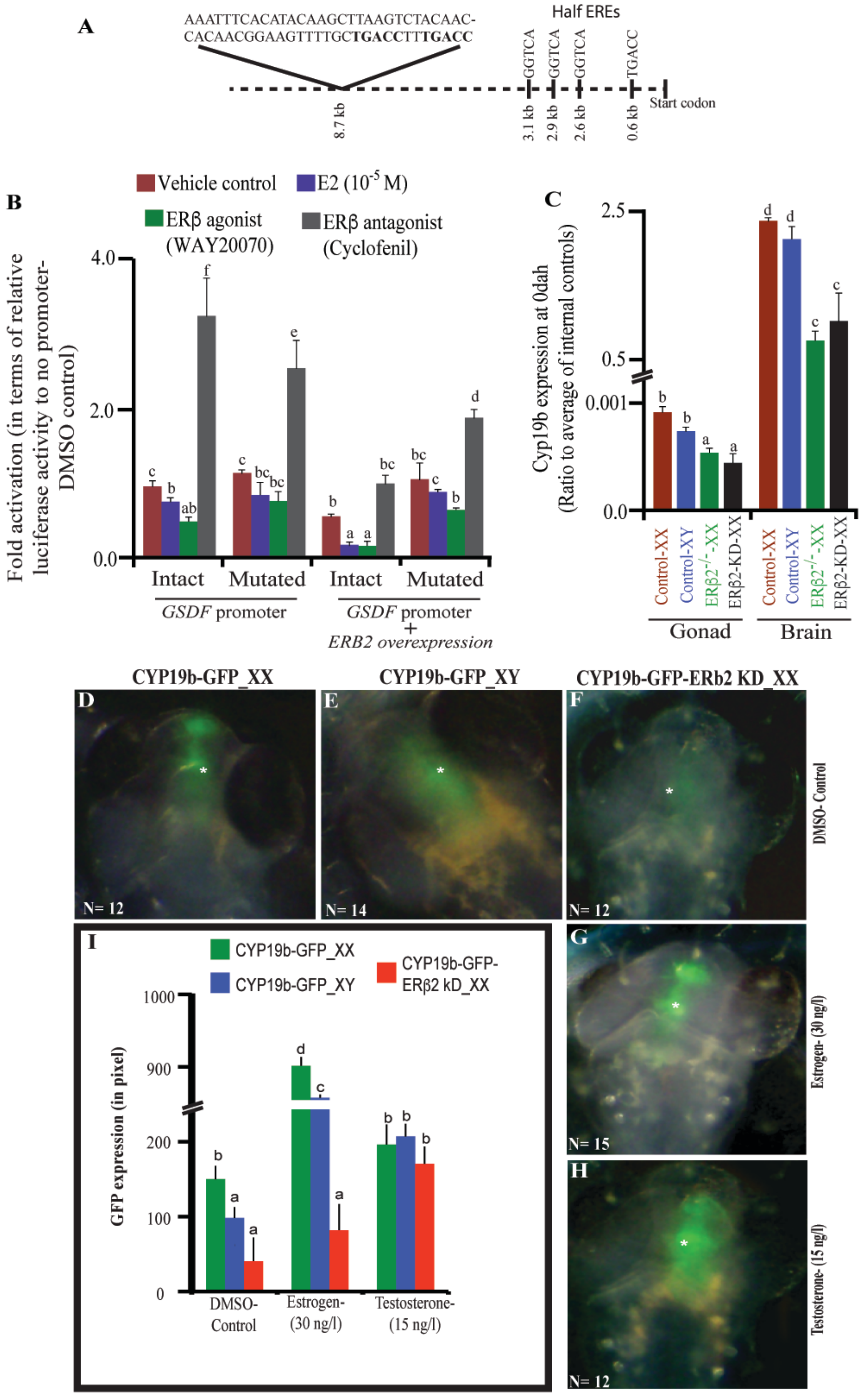

Figure S4. Evaluation of *ERβ2* association with sex biased gene expression. Related to Figure 1 and 3. A-B. 10kb upstream genomic sequences of GSDF start codon (obtained from Ensemble 95, https://asia.ensembl.org/Oryzias_latipes/Transcript/Exons?db=core;g=ENSORLG00000022492;r=12:10454505-10458561;t=ENSORLT00000030736) houses several potential half-EREs (A). Further *in vitro* dual luciferase analysis using both intact and mutated (deleted 60 bp sequence, marked in Figure S4A) and ER agonist, antagonist (with or without ERβ2 overexpression) confirmed ERβ2 associated GSDF promoter suppression. The experiments were repeated for 7

times. **C**. QPCR analysis demonstrating the variable *CYP19b* transcription profiles in brain and gonads of control-XX, control-XY, ERβ2-KD-XX and ERβ2-/- -XX embryos at 0dah. Triplicates, each containing 10 embryonic gonad and brain were used for QPCR analysis. **D-I.** The crossbreeds of ERβ2-KD-CYP19b-GFP-XX females and either of CYP19b-GFP-XY males or ERβ2-KD-XX males were treated with DMSO (vehicle control, C-E), Estrogen (F) and testosterone (G) for 3 hours. The fluorescence intensity of each GFP positive whole brain were captured using Z stack function of confocal microscope (LSM 710, Zeiss, Germany) and subsequently analysed with IMAGE J64 software. The GFP expression (N=11) was plotted on Y-axis and different treatments on X-axis (H). Note: In graphs, data are plotted as means ± SEM; different letters denote significant differences at $p<0.05$.

Figure S5

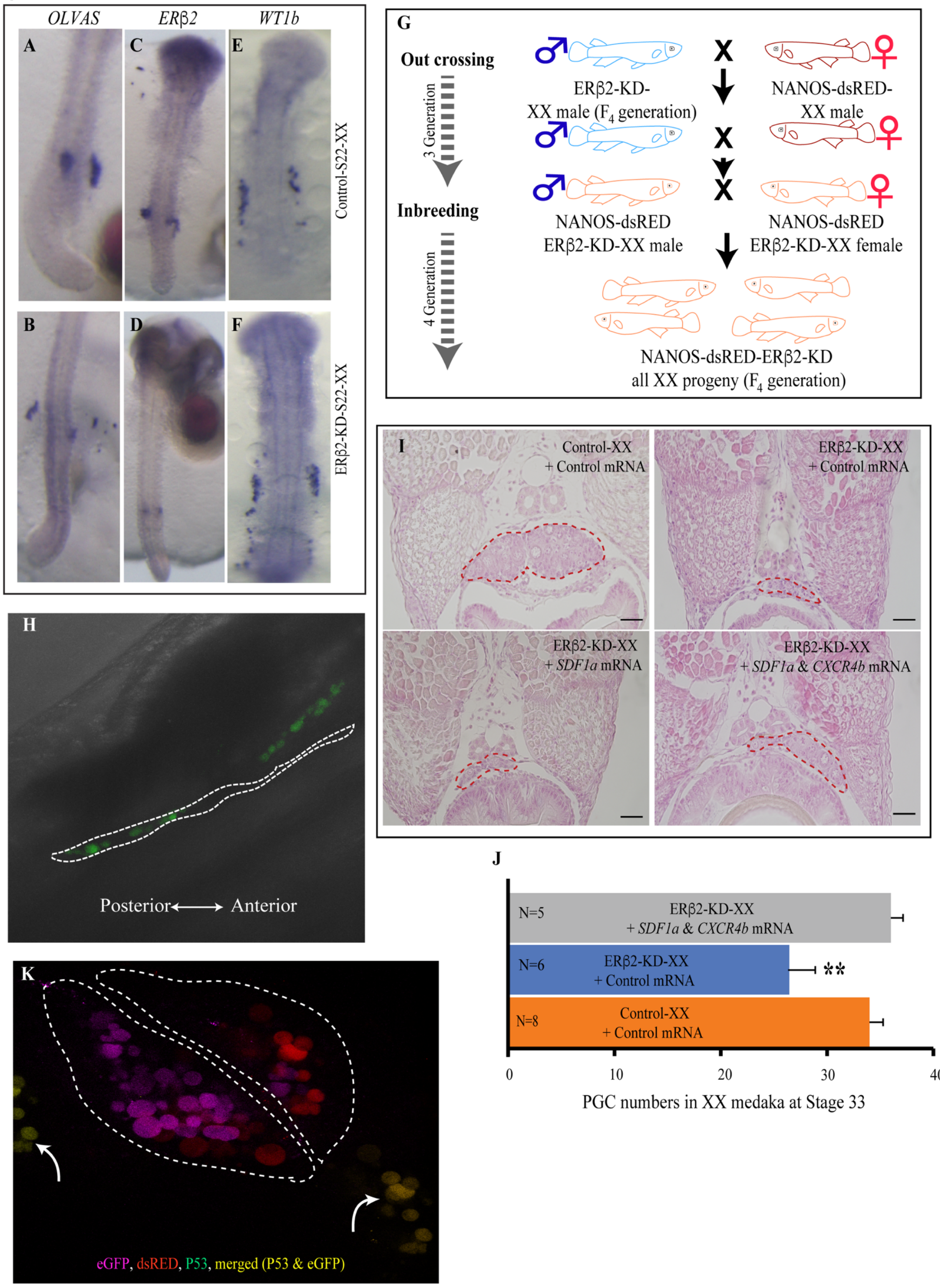

Figure S5. Characteristics of PGCs in ERβ2-KD-XX and ERβ2$^{-/-}$ XX fish. Related to Figure 3. A-F. *WISH* analysis showed abnormal migration of germ cells in ERβ2-KD-XX stage22 medaka embryos (B, D and F) compared to their respective controls (A, C and E). *OLVAS* acted as the germ cell marker, while *WT1b* was used as somatic cells marker in the gonadal primordium. **G.** NANOS-dsRED-ERβ2-KD lines were generated by series of out crossing and inbreeding techniques. Similar methodology was employed to develop OLVAS-eGFP-ERβ2-KD and OLVAS-eGFP-ERβ2-KO (first ERβ2$^{+/-}$ and then ERβ2$^{-/-}$) line. **H.** Similar to ERβ2-KD medaka (see also Figure 3, Table S1), germ cells in homozygous ERβ2-KO medaka failed to localize in the gonadal anlagen (marked with dotted white boundary) at stage22. So, for subsequent analysis we focused on ERβ2-KD medaka. **I-J.** Althogh singular overexpression of either SDF1a or CXCR4b failed to rescue the PGC mismigration, the co-overexpression of *SDF1a* and *CXCR4b* resulted in significantly increased PGC numbers in comparison to the ERβ2-KD-XX fish [See also, Fig. 3G-I and Table S1]. The representative gonadal structures of different overexpressed groups clearly showed that, despite alterations in mitotic germ cell numbers (J), the gonadal size and germ cell count of ERβ2-KD-SDF1a/CXCR4b-overexpressed-XX fish were still less than the Control-XX counterparts (I). Note: In graph, asterisk "*" indicates significant difference at p<0.01. Red dotted line indicates the gonadal boundary. Scale bar, 50µm. **K.** NANOS-dsRED-ERβ2-KD PGCs were transplanted into OLVAS-eGFP host embryos to analyse the *ERβ2* responsive PGC mismigration and death at 10dah (N=10). Note: White dotted line indicates the gonadal boundary and white arrows points out the mismigrated and dying germ cell population.

Figure S6

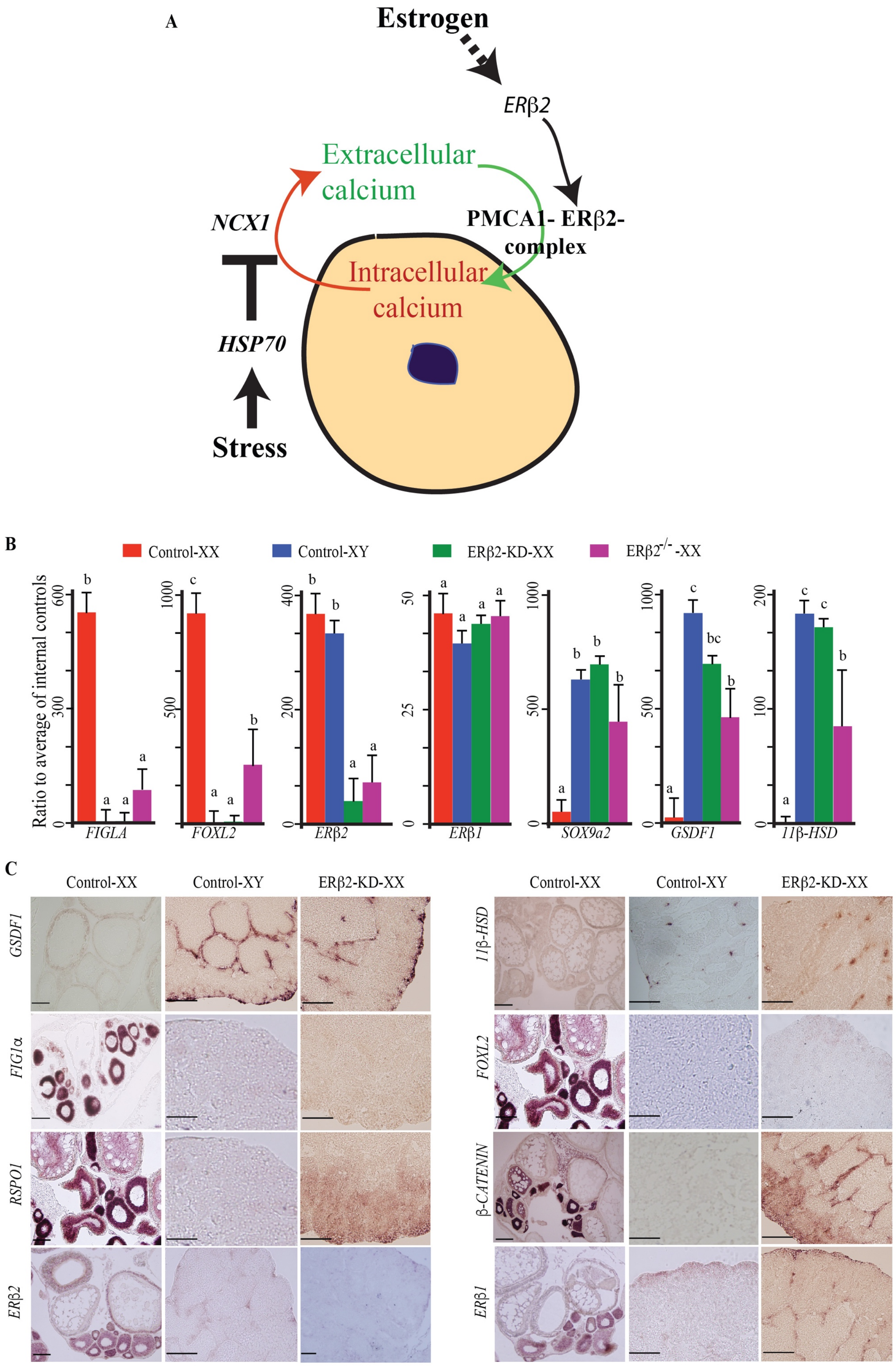

Figure S6. Effect of ERβ2 on calcium ion balance and functional sex reversal. Related to Figure 4 and Figure 5. A. Schematic diagram representing the ERβ2 and calcium ion balance. Estrogen, via ERβ2, regulates the PMCA genes, and

advertently affects the calcium ion transport from the germ cells extracellular to the intracellular region. This change in Ca^{+2} balance in germ cell probably influences the degeneration process of the cell and thereby fuelled functional sex reversal. **B-C.** The transcriptional changes (B) and cellular localization (C) of various sex-biased genes were assessed in the adult Control-XX, Control-XY and ERβ2-KD-XX, and ERβ2$^{-/-}$ -XX fish to determine the gonadal sexuality and the extent of sex reversal. Note: QPCR analysis were performed using 10 individual samples from each group. Phenotypically male like individuals of ERβ2-KD-XX, and ERβ2$^{-/-}$ -XX groups were only included in the QPCR and *ISH* analysis. Scale bar, 50μm.

Supplemental Tables

Table S1. Summary of rescuing of PGC migration in ERβ2-KD XX and ERβ2$^{-/-}$-XX medaka. Related to Figure 3.

**Fish group**	**Control-XX-control mRNA over expression**	**ERβ2-KD-XX-control mRNA over expression**	**ERβ2$^{-/-}$-XX-control mRNA over expression**	**ERβ2-KD-XX-*SDF1a* mRNA-over expression**	**ERβ2-KD-XX-*CXCR4b* mRNA-over expression**	**ERβ2-KD-XX-*SDF1a* & *CXCR4b* mRNA-co-over expression**	**ERβ2$^{-/-}$-XX-*SDF1a* & *CXCR4b* mRNA-co-over expression**	**Conrol-XX-*SDF1a* & *CXCR4b* mRNA-co-over expression**
Type of mRNA used	Capped mCherry/Cyan	Capped mCherry/Cyan	Capped mCherry/Cyan	Capped *SDF1a* 5`utr-orf-mCherry-3`utr	Capped *CXCR4b* 5`utr-orf-mCherry-3`utr	Capped *SDF1a* 5`utr-orf-mCherry-3`utr and Capped *CXCR4b* 5`utr-orf-mCherry-3`utr	Capped *SDF1a* 5`utr- orf-mCherry-3`utr and Capped *CXCR4b* 5`utr-orf-mCherry-3`utr	Capped *SDF1a* 5`utr- orf-mCherry-3`utr and Capped *CXCR4b* 5`utr-orf-mCherry-3`utr
Amount of mRNA used	5 ng each	5 ng each	5 ng each	5 ng each	5 ng each	5 ng each	5 ng each	5 ng each
Number of fish injected	15*	10	7	10	10	15	15	15*
Number of XX fish examined for live germ cell counting at Stage (S)22	7	9	6	10	8	12	11	6
Number of fish examined for live germ cell counting at S33	7	9	6	10	7	12	10	6
Total number of eGFP positive germ cells at S22 (± Standard deviation)	19.7 ± 1.6	18.4 ± 1.4	18.3 ± 1.1	18.0 ± 2.1	18.5 ± 1.5	18.6 ± 1.4	18.9 ± 1.1	19.3 ± 1.4
Average number of germ cell mismigrated at S22 (± Standard deviation)	0.3 ± 0.6	7.3 ± 2.2	3.3 ± 6.2	6.5 ± 1.2	5.4 ± 0.7	1.1 ± 0.5	0.5 ± 0.7	0.3 ± 0.8
Average number of germ cell reached at gonadal primordium at	19.4 ± 1.7	6.7 ± 5.6	9.2 ± 9.8	7.7 ± 5.8	7.0 ± 6.7	14.1 ± 7.1	15.1 ± 7.4	19.0 ± 1.2

S22 (± Standard deviation)								
Total number of eGFP positive germ cells at S33 (± Standard deviation)	38.9 ± 1.7	25.1 ± 1.9	32.1 ± 7.5	30.0 ± 1.3	31.6 ± 1.0	39.7 ± 1.3	39.6 ± 1.2	39.5 ± 1.4
Average number of germ cell mismigrated at S33 (± Standard deviation)	0.7 ± 0.7	11.0 ± 4.2	6.6 ± 6.8	9.0 ± 1.6	9.6 ± 1.4	1.7 ± 1.1	0.9 ± 0.8	0.8 ± 0.6
Average number of germ cell reached at gonadal primordium at S33 (± Standard deviation)	38.1 ± 1.7	8.5 + 8.1	19.8 + 18.2	14.0 ± 10.0	10.3 ± 11.0	30.4 ± 15.3	34.1 ± 11.4	38.7 ± 1.1
Number of fish with meiotic cells in gonad at 10dah (days after hatching)	4 (N=4)	0 (N=6)	2 (N=6)	0 (N=7)	0 (N=4)	8 (N=8)	9 (N=9)	3 (N=3)
Average number of germ cells at 10dah (± Standard deviation)	470.5 ± 17.6 (N=4)	111.5 ± 5.0 (N=6)	179.8 ± 120.5 (N=6)	142.9 ± 8.6 (N=7)	146.8 ± 6.7 (N=4)	252.8 ± 22.2 (N=8)	362.8 ± 20.1 (N=8)	482.5 ± 7.3 (N=3)
ERα mRNA content (N=3) (± Standard deviation)	8.1E-06 ± 3.3E-06	4.2E-06 ± 5.0E-06	4.1E-06 ± 2.5E-06	6.0E-06 ± 8.6E-06	4.0E-06 ± 1.2E-06	7.1E-06 ± 2.5E-06	7.2E-06 ± 1.3E-06	7.1E-06 ± 1.7E-06
ERβ1 mRNA content (N=3) (± Standard deviation)	2.9E-05 ± 3.2E-05	2.4E-05 ± 1.6E-05	2.3E-05 ± 1.8E-05	3.2E-05 ± 9.5E-05	3.2E-05 ± 6.0E-05	2.5E-05 ± 9.1E-06	2.5E-05 ± 4.1E-06	2.5E-05 ± 6.3E-05
ERβ2 mRNA content (N=3) (± Standard deviation)	2.1E-05 ± 5.0E-05	7.6E-06 ± 2.3E-06	7.3E-06 ± 2.1E-06	1.2E-06 ± 6.8E-06	2.4E-06 ± 1.5E-06	8.6E-06 ± 9.0E-06	8.4E-06 ± 3.4E-06	4.7E-05 ± 2.9E -05

Note: Asterisk (*) indicates that control fish were later segregated based on genetic sex.

Table S2. Summary of ERβ2 knockdown in medaka. Related to Figure 1 and Figure 5.

Stage of Sampling (dah; days after hatching)	Analysis method	Number of sample (N)	Number of fish with changes[9]
	**F_0 generation**		
**0dah**	QPCR[1]	10	9
	Histology[2]	30	10
**10dah**	QPCR[1]	10	10
	Histology[2]	35	11
	ISH[3]	10	4
**50dah**	Histology[2]	10	3
**Adult**	Secondary sexual character[4]	12	5
	Breeding behaviour[5]	5	5
	Gene integration[6]	12	3
	**F_1 generation**		
**10dah**	Histology[2]		
	ISH[3]		
**Adult**	Secondary Sexual Characters[4]	50	16
	Genome integration[6]	14	14
	Breeding behaviour[5]	5	3
	**F_2 generation**		
**10dah**	Histology[2]	50	23
	ISH[3]	10	7
**Adult**	Secondary sexual character[4]	80	21
	Breeding behaviour[5]	5	3
	**F_3 generation**		
**2daf**	Germ cell migration[7]	9	9
**10dah**	Histology[2]		
	Rescue with E_2[1,2,3]	20	0
	Rescue with *ERβ* agonist[1,2,3]	20	2
	Rescue with *ERβ2* overexpression[1,2,3]	10	10
	Rescue with Flutamide[1,2,8]	20	20
**Adult**	Secondary sexual character[4]	42	41
	Breeding behaviour[5]	5	4

Note: Asterisk indicates the biological end points analysed using the samples. 1 - Change in gene expression; 2 - Change in germ cell number and occurrence of meiosis; 3 - Changes in cellular localization of different gene; 4 - Occurrence of Fan like anal fin and Forked Dorsal fin; 5 - Mating characteristics, i.e., chasing, dancing, coiling and pressing; 6 - Positive PCR amplification of antisense probe using a vector and gene specific primer pair; 7 - PGC migration and settlement in gonadal primordium; 8 - Changes in eGFP expression; 9 - Deviation from average (± standard deviation, if applicable) control samples, i.e., reduction in gene expression for QPCR, gonad size and germ cell number for histology, intensity (visual observation) and localization of *ISH* staining, variation from normal XX secondary sexual, changes in breeding behaviour, external gene integration, mis-migration (percentile ratio of total germ cell and mis migrated cells >5%) of germ cells, etc.

121 **Table S3. List of major primers used in this study. Related to Supplemental Experimental Procedure**

Primer name	Sequence	Peak Standard disscociation (^{0}C)	Peak amplicon dissociation (^{0}C)	Geometric amplification efficiency (%)	Purpose
GSDF Realtime F	GGGCTGGACACTATTCGAGA	82.5	82.5	99.11	Measuring Gene expression by QPCR, ORF amplification, *in situ* probe preparation, synthetic RNA preparation, ORF amplification, *in situ* probe preparation
GSDF Realtime R	CATGACACAGAGGAGCTGGA				
SF1 Realtime F	AGCTGCTACTCTGGAAACGA	81.5	81.5	100	
SF1 Realtime R	ACTGGCAATCTTCTTGGCAGC				
GFP Realtime F	CGACAACCACTACCTGAGCA	81.5	81.5	99.23	
GFP Realtime R	GAACTCCAGCAGGACCATGT				
Vasa Realtime F	CCCAAAGTGACCTACATC	81.0	81.0	99.76	
Vasa Realtime R	AAGTTGATGCCCATCTTG				
Foxl2 Realtime F	AAACCTGCTACTCTGGACGC	82.0	82.0	99.45	
Foxl2 Realtime R	AGTCAAATCTTCTTGATTC				
Fig1a Realtime F	TGTACTGCTGCATCGAGAAGTA	82.5	82.5	99.74	
Fig1a Realtime R	ATGCTGCAACACCAGTCTAGT				
Spo11 Realtime F	TCGATTCTGGTGCCGTCTTCT	82.0	82.0	98.96	
Spo11 Realtime R	ATGCTGAAGGTTTCTCGCAGG				
RSPO 1 Realtime F	TGCAACACCAGTCTAATG	81.5	81.5	99.35	
RSPO 1 Realtime R	TTCTGGTGCCGTCTTCTAGG				
Cyp19a1 RT F	AGCTTATTTTTGCCCAAGGCC				
Cyp19a1RT R	TTGAGCAGCAGGAGCATGAAA				
GSDF ORF F	ATGTCTTTGGCACTCATT				
GSDF ORF R	CTACTTTTTGCAGGGCTGCT				
SF1 ORF F	AGCAAGGGTGTGAGGAG				
SF1 ORF R	TTTTTGCAGGGCTGCT				
DMY ORF F	CCGCGGGAGCTCATGAGCAAGGAGAAGCAGTGC				
DMY ORF R	GGATCCGAATTCTGGAGTTGGCCGGGAAGACG				
SPO11 F	GATGCAAGGAGAGAGTT				
SPO11 R	ATACTCAGCTGTTTGGGTCACA				
FOXL2 F	TGCACCTGACACCAGTCT				
FOXl2 R	TAGACAACACCGAGTCTG				
Vasa F	AAGAGCTCCCAGCAAGGC				
Vasa R	TCGGAGCTCATGAGCAAGG				
RSPO F	TGCAACACCAGTCTAATG				
RSPO R	TAGACTGCCCGTCATG				
Fig1a F	ATGAAGGTGCCAGAGGCGGAAT				
Fig1a R	TTAATCCCTCGAAGCTTGATCG				
ERβ2 as Xba1-F1	TCTAGAAGACGAAGGCCAGATCCT				Amplification of *ERβ2* antisense region1, integration check
ERβ2 as Eco RV R1	GATATCACCCCTGTAAGGTTGTT				
M13 F	GATATCCGAGCATCTCCAGTAGGAGG				Colony PCR, Sequencing, Genomic PCR for sorting of sex
M13 R	TCTAGATCTACAGCATGAAGTGCAA				
T7	GATATCGACCCTCCATACTGAAGGA				
T3	TCTAGACTCTTTGTTCTGGCAAAGCC				
Sp6	GATATCGGTTGCAGGGCAGATGTAGT				

Bgh R	TCTAGAATGGGAACCACTTTGGACTC
pcDNA 3.1 807 F	GATATCAGCTGAAGATGGTTGGGTTG
PCDNA 3.1 1127 R	ATGAAGGTGCCAGAGGCGGAAT
DMY genomic F	TTAATCCCTCGAAGCTTGATCG
DMY genomic R	TCTAGAAGGAGGAGCTTGGGATTTGT

Supplemental Experimental Procedures

Plasmid constructs

The anti-sense RNA (AS-RNA) expression constructs were designed and evaluated using the online tool E-RNAi (Chakraborty et al., 2016). The sequence was also analyzed for secondary structures *in silico*, using RNA*fold* online software (http://rna.tbi.univie.ac.at/cgi-bin/RNAfold.). A selected primer pair carrying *Xba*I and *EcoR*V sites (Table Supplemental experimental procedure) was used to amplify a 220-bp region of the medaka (m) *ERβ2* gene (Figure S2) from a plasmid containing the complete *ERβ2* ORF (Chakraborty et al., 2011). The amplicon was cloned under the control of the CMV early promoter in pcDNA3.1 (+) (Invitrogen, USA) vector in the antisense orientation. The constructs were transformed into *E. coli*-XL1 blue and the positive clones were confirmed by sequencing (hereafter named as pmERβ2-AS). Similarly, the tet-on vector (Clontech, USA) was used to prepare the conditional ERβ2 knockdown (ERβ2-KD tet-on) construct. Plasmid DNA, used for downstream experiments, was purified using the plasmid purification kit (Qiagen, Germany).

Expression plasmids were constructed using pcDNA3.1(+) vector backbone using complete ORFs of required genes, if not specifically mentioned. pGEMt-easy plasmid of different genes was used for *in situ hybridization* (*ISH*) probe synthesis and QPCR standard preparation, whenever necessary. 4-6 kb long promoter fragment of related genes were isolated using specific primer set, and cloned in pGL3 basic vector (Promega, USA using Infusion-cloning kit (Clonetech, USA). *ERβ2*, *CXCR4b* and *SDF1a* fragments (containing 5'UTR and stop codon less ORFs) were in-framed with mCherry (source: pmCherryN1 vector) and Cyan (source: pamCyanN1 vector) ORF followed by 3` UTR of respective genes and cloned into a pCS2 vector, using Infusion cloning kit (Clontech, USA).

Gene knockdown in HEK-293 cells

Knockdown of *ERβ2* expression by the pmERβ2-AS construct was tested in HEK-293 cells with no endogenous expression of fish *ER*s. HEK-293 cells were seeded in 24-well plates at 5 X 10^5 cells/well in Dulbecco's modified Eagle's Medium (Sigma, USA) supplemented with 10% charcoal/dextran treated fetal bovine serum (Hyclone, USA). The cells were incubated for 24h at 37^0C with 5% CO_2 and co-transfected with either pCMV-mERα/ mERβ1/mERβ2 plasmid (100ng) and 5, 10 and 100ng of the pmERβ2-AS construct using Fugene-6 transfection reagent (Roche Diagnostics, Switzerland) following the manufacturer's instructions. Cells transfected with only pCMV-mER(s) served as positive controls, while the negative controls were treated with only the transfection reagent. The cells were grown for 96h after which total RNA was isolated and target transcripts were quantified by QPCR, as detailed later. The transfections were performed in triplicate at one time point and then repeated twice later. The transcript copy numbers are shown as the mean ± SEM and the dose-response data were computed using Statview v. 5 (SAS institute Inc, Cary NC).

Experimental animals

The QurtE strain of medaka was used for this study, if otherwise not mentioned. This strain expresses a male-specific leucophore, which allows easy sexing of fish. Other strains, such as OLVAS-eGFP, NANOS-dsRED, transgenic medaka were also used. These strains carry the *eGFP/dsRED* gene under *OLVAS* promoter or *NANOS 3'* utr and were reported to express respective fluorescence in germ cells of both males and females (Tanaka et al., 2001; Kurokawa et al., 2006). All the fish were maintained at 26 ± 2^0C under a 14h light and 10h dark cycle. Eggs were collected within 30 min of fertilization and incubated in distilled water (milli-Q), containing antifungal solution (Methylene blue, 0.0001%) at 26 ± 2^0C. Brooders and juveniles were fed with fresh artemia, while larvae were given artificial food. All *in vivo* experiments and fish samplings were conducted following protocols and procedures approved by Institutional Animal care and use committee at the National Institute of Basic Biology, Japan, and Ehime University Animal Use and Ethics Committee,

Japan. Additionally, genetic sex of each individual was examined from fin clips, tail bud, etc., using DMY-genomic PCR (Matsuda et al., 2002), either before pooling samples for subsequent analysis or after live analysis, as per convenience.

Chemicals/steroid treatment

One/two cell staged fertilized medaka embryos (100 embroys/50ml water) were treated till 18 days after fertilization (daf) (if not otherwise specifically mentioned) with Vehicle DMSO/Ethanol (Nacalai Tesque, Japan), ERβ agonist (WAY20070, Tocris bioscience, USA), ERβ antagonist (cyclofenil, Tocris bioscience, USA), 17β-estradiol (E_2) (Sigma, USA), Calcium chloride ($CaCl_2$) (Nacalai Tesque, Japan), extracellular calcium chelator (EGTA, Sigma, USA), intracellular calcium chelator (BAPTA_AM, Sigma, USA), and Doxycycline (Gibco, USA). The embryos were reared as mentioned above with daily water exchange.

Histology, *in situ hybridization* (*ISH*), wholemount *ISH* (*WISH*), fluorescent *WISH* (*FWISH*), and fluorescent immunohistochemistry (FIHC)

Bouin fixed, paraffin embedded samples were used for standard Hematoxylin & Eosin (HE) staining, while, 4% paraformaldehyde fixed samples were used for *ISH, WISH,* and *FWISH.* All the histological analyses were performed using 5μm sections. For *ISH*, sense and anti-sense digoxigenin-labelled RNA probes were transcribed *in vitro*, using RNA labelling kit (Roche Diagnostics GmbH, Germany), from plasmid DNA containing the respective genes. Sections were deparaffinised, hydrated, treated with proteinase K at 10µg/ml (Roche), and hybridized with the sense or anti-sense labelled RNA probes at 58^0C for 18-24 h. The hybridization signals were detected following previously published protocols (Chakraborty et al., 2011). *WISH* was performed using previously published protocols (Mohapatra et al., 2015). The FIHC procedures are same as previously described (Inoue and Wittbrodt, 2011). Briefly, PFA fixed samples were treated for antigen retrieval, peroxidase activity and blocked in 5% FBS-PBS, incubated with primary (1:10000 dilution) and Alexa tagged secondary (1:20000 dilution) antibody, and micro graphed using confocal microscope (LSM 710, Zeiss, Germany). LC3 (GeneTex, GTX127375) and OLVAS (Genetex, GTX128306) antibodies were used in our study.

Quantification of changes in gene expression by QPCR

Changes in gene expression were quantified using the ABI Prism 7000 sequence detection system (Applied Biosystems, USA). Total RNA was isolated from cells, embryos or gonads using RNeasy Mini kit (Qiagen, Germany). cDNA synthesis was carried out using a Quantitect RT PCR kit (Qiagen) from 100 ng of total RNA. The first strand cDNAs were diluted to 100µl for subsequent use. Gene-specific QPCR was performed using SYBR green master mix (Applied Biosystem) and 5 ng of cDNA, according to the manufacturer's instructions. The PCR conditions included an initial denaturation at 94^0C (2 min) followed by 40 cycles at 94^0C (30 s) and 60^0C (1 min). *Ef1α* and *rps18* were used as the internal controls. The absolute transcript copy number of each gene was determined with the help of appropriate standard curves and normalized with the average of *ef1α* and *rps18* copy numbers in each sample. The reported values are averaged from experimental triplicates, if not otherwise mentioned. The specificity of primer sets, throughout this range of detection, was confirmed by the observation of a single amplification product of the expected size, melting curve, Tm (melting temperature) and sequences. All assays were quantified, with standard curves (mean Ct vs. log cDNA dilution) having slopes between -2.99 and -3.34, a linear correlation (R^2) between the mean Ct and the logarithm of cDNA dilution of >0.985 in each case. All test cDNAs were run in duplicates for each gene. The primers were designed according to unmutated (wild) DNA sequences, if not otherwise mentioned.

Promoter analysis

4-6 kb promoters of *WT1b*, *SDF1a*, and *CXCR4b* were isolated from medaka genomic DNA and directionally cloned in pGL3 promoterless luciferase vector. The promoter analysis was performed using previously described protocol (Chakraborty et al., 2011). Briefly, HEK-293 cells were seeded in 24-well plates at 5 X 10^5 cells/well in Dulbecco's modified Eagle's Medium (Sigma, USA). After 24h, the cells were transfected with pGL3-promoter-luciferase plasmid and pcDNA3.1-ERβ2 plasmid at different concentration (0-200ng/well) in triplicates. The luciferase assay was performed after 48 hours of transfection. The experiment was repeated thrice for reproducibility.

Knockdown of ERβ2 expression in medaka embryos

One-two cell embryos of the QurtE strain of medaka were electroporated with the pmERβ2-AS plasmid construct (1μg/ml) in 1X HBS buffer, pH 7.53 (Chakraborty et al., 2016). Electroporation was carried out using 4mm gap cuvette (BTX, USA) in a Cuy21 edit type electroporator (Bex, Tokyo) at 24V, with a 9 millisecond (ms) pulse duration, a 900 ms pulse interval and 3 pulses. Embryos electroporated with only HEPES buffered saline (HBS) served as control. No significant difference in lethality was observed between pmERβ2-AS (7.1±3.5/200 fertilized egg), empty vector (6.8±3.6/200 fertilized egg) electroporated and non-electroporated (7.2±3.1/200 fertilized egg) control. DNA was extracted from 10 randomly collected individuals and plasmid DNA was PCR amplified and sequenced to confirm the electroporation. Importantly, 9/10 pmERβ2-AS and 10/10 empty vector electroporated embryonic DNA showed PCR amplification, suggesting higher incorporation rate.

Electroporated female embryos of the QurtE strain of medaka were sampled at 0, 10, 50 and 120 (adult) days after hatching (dah) and to check the morphological changes in gonad and confirm gene knockdown at the genetic and phenotypic level. At least, 10 fish were sampled at each time point. The genetic sex of all fish was confirmed by dmy-genomic PCR (Matsuda et al., 2002).

Assessment of the transgenerational knockdown effect

The QurtE embryos injected with pmERβ2-AS were grown to adulthood. Based on integration PCR, genetic sexing, secondary sexual characters and the absence of leucophores, genetic females (sex reversed to males) were identified and outcrossed with normal females to assess their breeding behavior and mating performance (Table S2). Successfully mated XX males were used to produce F_1 progeny. Caudal fin clips from XX males of the F_0 and F_1 generations were used for genomic DNA isolation (Matsuda et al., 2002). The genome integration of the pmERβ2-AS construct was tested using a primer pair that amplified a portion of the vector and *ERβ2* antisense sequence (Table S1), followed by sequencing. Aliquots of the same genomic DNA samples were analyzed by QPCR to estimate the number of integrated copies. The breeding experiment was similarly performed for F_1 and F_2 fish and the offsprings were histologically examined at 10 and 20dah (Table S2). All other experiments were conducted using F_4 or later generation fish.

Rescue of PGC migration

To rescue the *ERβ2* knockdown effect on PGC migration, the F_4 generation fish developed from OLVAS-eGFP-ERβ2-KD-XX line were used. Briefly, *sDF1a*-Cyan and *cxcr4b*-mCherry mRNA were synthesized with mMESSAGE mMECHINE SP6 kit (Ambion), following polyA addition with Poly-A tailing kit (Ambion). The purified RNA(s) were injected in one/two cell stage embryos of OLVAS-eGFP-ERβ2-KD-XX fish @ 1ng/μl. Each embryos were monitored at stage 15, 22, 27 and 32, using confocal microscope (LSM 710, Zeiss, Germany). The Z-stage confocal sections of each live embryo were further analysed to ascertain the PGC numbers at each stage. The embryos showing reporter gene expression were then grown separately until 10dah on a 24 well dish @ 1 embryo/well and fixed/preserved for histological/QPCR analysis.

Chromatin immunoprecipitation (ChIP)

Atleast 25Kb upstream sequence (from the ORF start site) were obtained from ensemble (http://asia.ensembl.org/index.html), and the promoter regions were identified using promoter 2.0 prediction server (http://www.cbs.dtu.dk/services/Promoter/). 10Kb genomic region, including the promoter region, was further analyzed with Dragon ERE finder version 3.0 (http://datam.i2r.a-star.edu.sg/ereV3/), and two most potential ERE sites were predicted. For *in vivo* ChIP analysis, the ChIP expression-shearing kit (Active motif) was used according to the manufacturer's instructions, using 20mg of XX or XY embryonic samples (10 fish each), and ERβ antibody (3mg, Active motif, 39767). Preliminary experiments were conducted with 20mg of embryonic samples from ERβ2-eGFP mRNA injected fish and eGFP monoclonal antibody (3mg, AbCAM, ab184601), to validate the ER specificity. The ERβ2-eGFP plasmids was constructed by sequentially fusing *ERβ2*-5`UTR, *ERβ2*-ORF (stop codon less), *eGFP* and *ERβ2*-3`UTR into a pCS2 vector, using infusing cloning kit (Clonetech). RNA was synthesized with mMESSAGE mMECHINE SP6 kit (Ambion, USA) following polyA addition using Poly A tailing kit (Ambion). After tissue disaggregation and cell re-suspension, DNA was sheared, according to the manufacturer's protocols. The ChIP procedure using ERβ2-eGFP mRNA was validated as described earlier (Chakraborty et al., 2016).

Germ cell transplantation

NANOS-dsRED-ERβ2-KD-XX stage 22 embryos were dissociated using Ringer's solution containing 1% citric acid trisodium and 0.1% trypsin (Defco) for 30 minutes and the single cell suspensions were filtered using 40mm cell strainer. The single cell suspensions were transferred onto a 120 mm glass dish filled with Ringer solution, supplemented with 5% FBS, 0.01% penicillin and 0.01% streptomycin. dsRED-PGCs were aspirated into a glass microneedle under a fluorescence stereomicroscope and 5-6 PGCs were transplanted into the marginal region of the blastodisc of Stage 10 dechorionated OLVAS-eGFP embryo. Single embryos were incubated in 96 well flat bottom culture dish with daily water exchange and hatched embryos were individually cultured till 10dah. Thereafter, the embryos were fixed in 4% paraformaldehyde and multicolor IHC was performed, using eGFP, dsRED and P53 antibodies.

Flow cytometry and cell sorting

Each embryo (from different groups), following sexing, were incubated in L15 media containing 0.25% Trypsin for atleast 30 min, with periodical shaking, to prepare a single cell suspension. The enzymatic digestion was stopped using 2% FBS and the cell suspension was immediately fixed with 2% formaldehyde-PBS for 15 min at 4^0C, washed 3 times with PBS and re suspended in blocking solution (PBS containing 2% FBS and 0.001% Triton X). One hour after incubation the cells were pelleted, incubated with Alexa-tagged antibodies for 1h, washed several times with PBST (PBS + 0.01% tween 20), and subjected to cell sorting using Cell Sorter SH800 (Sony, Japan), following pre-adjusted protocols of germ cell sorting. The required group of sorted cells (cells from atleast 1000 embryos) were collected (if not otherwise mentioned), and RNA was isolated using RNeasy FFPE kit (Qiagen, Japan), following manufacturer's protocol (if not specifically mentioned otherwise). The cDNA synthesis and QPCR was performed as described above. The GFP (MBL, D153-A48) and LC3 (GeneTex, GTX127375) antibodies used in our experiments.

Data analysis

All experiments were conducted for a minimum of three times (biological replicates) and statistical differences were assessed based on biological replicates, if not otherwise mentioned. Statistical differences in relative mRNA expression between various experimental groups were assessed by One or two-way ANOVA of normalized data, followed by

Tukey's test, or Student's t-test. All statistical analyses were performed using SPSS, version 22. All experimental data are shown as mean ± SEM. Differences were considered statistically significant at $p<0.05$, if not otherwise mentioned. The correlations were calculated using Pearson correlation coefficient method.

Supplemental References

Chakraborty, T., Shibata, Y., Zhou, L.Y., Katsu, Y., Iguchi, T., and Nagahama, Y. (2011). Differential expression of three estrogen receptor subtype mRNAs in gonads and liver from embryos to adults of the medaka, *Oryzias latipes*. Mol. Cell. Endocrinol. *333*, 47-54.

Chakraborty, T., Zhou, L.Y., Chaudhari, A., Iguchi, T., and Nagahama, Y. (2016). *Dmy* initiates masculinity by altering *Gsdf*/*Sox9a2*/*Rspo1* expression in medaka (*Oryzias latipes*). Sci. Rep. *6*, 19480.

Inoue, D., and Wittbrodt, J. (2011). One for all-a highly efficient and versatile method for fluorescent immunostaining in fish embryos. PLoS One 6:e19713.

Kurokawa, H., Aoki, Y., Nakamura, S., Ebe, Y., Kobayahi, D., and Tanaka, M. (2006). Time-lapse analysis reveals different modes of primordial germ cell migration in the medaka *Oryzias latipes.* Dev. Growth Differ. *48*, 209- 221.

Matsuda, M., Nagahama, Y., Shinomiya, A., Sato, T., Matsuda, C., Kobayashi, T., Morrey, C.E., Shibata, N., Asakawa, S., Shimizu, N., et al. (2002). DMY is a Y-specific DM-domain gene required for male development in the medaka fish. Nature *417*, 559-563.

Mohapatra, S., Chakraborty, T., Miyagawa, S., Zhou, L.Y., Ohta, K., Iguchi, T., and Nagahama, Y. (2015). Steroid responsive regulation of IFNg alternative splicing and its possible role in germ cell proliferation in medaka. Mol. Cell. Endocrinol. 400, 61-70.

Tanaka, M., Kinoshita, M., Kobayashi, D., and Nagahama, Y., (2001). Establishment of medaka (*Oryzias latipes*) transgenic lines with the expression of green fluorescent protein fluorescence exclusively in germ cells: A useful model to monitor germ cells in a live vertebrate. Proc. Natl. Acad. Sci. USA *98*, 2544-2549.
